# Fibroblast-mediated KRAS activation in double-negative prostate cancer

**DOI:** 10.1038/s41419-026-08800-3

**Published:** 2026-05-02

**Authors:** Taiki Kamijima, Kouji Izumi, Kaoru Hiratsuka, Takahiro Inaba, Yoshiki Koketsu, Ryunosuke Nakagawa, Ren Toriumi, Shuhei Aoyama, Hiroshi Kano, Tomoyuki Makino, Renato Naito, Suguru Kadomoto, Hiroaki Iwamoto, Hiroshi Yaegashi, Shohei Kawaguchi, Takahiro Nohara, Kazuyoshi Shigehara, Hiroki Nakata, Yohei Saito, Kyoko Nakagawa-Goto, Wen-Jye Lin, Atsushi Mizokami

**Affiliations:** 1 Department of Integrative Cancer Therapy and Urology, Kanazawa University Graduate School of Medical Science, Kanazawa, Japan; 2 Department of Clinical Engineering, Faculty of Health Sciences, Komatsu University, Komatsu, Japan; 3 School of Pharmaceutical Sciences, College of Medical, Pharmaceutical and Health Science, Kanazawa University, Kanazawa, Japan; 4 Immunology Research Center, National Health Research Institutes, Zhunan, Taiwan

**Keywords:** Prostate cancer, Growth factor signalling

## Abstract

When androgen receptor (AR) signaling is suppressed, prostate cancer progression is inhibited; however, many patients eventually relapse, developing castration-resistant prostate cancer (CRPC). Recently, the incidence of double-negative CRPC (DNPC)—which lacks AR and neuroendocrine activity—has increased, yet effective treatments remain unavailable. Our research demonstrated that KRAS has minimal influence during the AR-dependent stages of prostate cancer but significantly activates cancer cells when AR signaling is suppressed. Further investigation revealed that AR inhibition modifies fibroblast growth factor receptor expression in prostate cancer cells. Additionally, CCL2, secreted by AR-inhibited prostate cancer cells, induces FGF8b secretion from stromal cells within the tumor microenvironment, which in turn enhances KRAS activation. A pan-KRAS inhibitor effectively suppressed AR-independent prostate cancer cells by disrupting KRAS-mediated cell survival signaling. This inhibition led to the significant induction of programmed cell death, characterized by the downregulation of the anti-apoptotic protein BCL-xL and the promotion of apoptosis as evidenced by increased cleaved caspase-3 in vivo. These findings highlight KRAS activation, driven by the CRPC microenvironment, as a critical factor in DNPC progression and identify the induction of KRAS-targeted cell death as a promising therapeutic strategy for DNPC.

## Introduction

Prostate cancer is the most common cancer among men and ranks as the second leading cause of cancer-related deaths [1]. Prostate cancer cells are highly reliant on androgens and are treated by suppressing androgen receptor (AR) signaling [2]. Nevertheless, many patients eventually relapse, resulting in the development of castration-resistant prostate cancer (CRPC). In CRPC, AR remains active through alternative activation pathways, necessitating the use of more potent secondary therapies to inhibit AR signaling. Recently, second-generation AR signaling inhibitors (ARSIs) have been introduced, which have significantly enhanced the prognosis for prostate cancer patients [3–7]. Despite these advancements, CRPC ultimately develops resistance to ARSIs and, in most cases, transitions to a state entirely independent of AR [8]. The mechanisms that sustain prostate cancer cell growth in the absence of AR remain poorly understood. Double-negative CRPC (DNPC), defined by the absence of AR-driven prostate-specific antigen (PSA) expression and neuroendocrine differentiation (NED), is characterized by local prostate regrowth [9–11] and has no established treatments, leading to a poor prognosis. FGF [12, 13], HGF [14], and CCL2 [15] have been associated with DNPC, though the precise cause remains uncertain. Comprehensive cancer genomic profiling identified KRAS gene aberrations in DNPC patients. However, unlike lung and colon cancers, KRAS alterations are rare in prostate cancer. We hypothesized that canonical KRAS activation could be associated with DNPC, given that fibroblast growth factor receptor (FGFR) serves as a critical upstream regulator of KRAS. Additionally, prostate cancer-associated stromal cells are known to secrete various growth factors [16]. We proposed that the paracrine-like release of activation factors could amplify KRAS activation within the local tumor microenvironment, thereby driving DNPC progression. This study is the first to propose a potential mechanism for DNPC development that involves interactions between AR-independent prostate cancer cells and stromal cells.

## Materials and methods

### Sex as a biological variable

Our study exclusively examined male patients and mice because the disease modeled is only relevant in males.

### Antibodies, recombinant proteins, and chemicals

All antibodies, recombinant proteins, and chemicals used in this study are listed in Table S1.

### Cell culture

The human prostate cancer cell lines LNCaP, C4-2B, PC-3, and DU145, as well as the human prostate stromal cell line PrSC, were obtained from the American Type Culture Collection (Manassas, VA, USA). LNCaP cells (androgen-sensitive), C4-2B cells (androgen-independent), and PC-3 cells (androgen-independent) were cultured in RPMI-1640 medium (30264-56; Nacalai Tesque) supplemented with 5% fetal bovine serum (FBS) and 1% penicillin/streptomycin. DU145 cells (androgen-independent) were maintained in Dulbecco’s modified Eagle’s medium (DMEM, 08459-64; Nacalai Tesque) with 5% FBS and 1% penicillin/streptomycin. The LNCaP-SF cells (androgen-independent prostate cancer cell line) were developed in our laboratory by long-term subculturing of parental LNCaP cells in RPMI-1640 medium supplemented with 5% charcoal-stripped FBS (CCS; HyClone, Logan, UT, USA) and 1% penicillin/streptomycin (Cytiva, Tokyo, Japan) [17]. The human prostate cancer-associated stromal cell lines PCaSCv2-4, PCaSCv2-5, PCaSCv2-6, and PCaSCv2-9 were established from consented prostate cancer patients. The method used for establishing these cell lines followed previously published protocols [18, 19]. In short, prostate tissue was collected by biopsy from areas confirmed to contain cancer by imaging, then minced into small pieces with scissors and washed twice with PBS. The fragments were then digested in 0.25% trypsin–EDTA (Invitrogen) for 30 min at 37 °C. After digestion, the dispersed stromal cells were cultured in RPMI supplemented with 1% penicillin/streptomycin and 10% FBS on 6 cm dishes. These cell lines were maintained in RPMI-1640 medium with 10% FBS and 1% penicillin/streptomycin. All cells were cultured in a humidified 5% CO_2_ environment at 37 °C. All cell lines were confirmed to be free of mycoplasma infection before the start of the study.

### Knockdown of KRAS and AR

KRAS-specific small interfering RNAs (siRNAs) (siKRAS1 [HSS105871], siKRAS2 [HSS105872], and siKRAS3 [HSS180200]) and androgen receptor-specific siRNA (siAR1 [HSS100619], siAR2 [HSS179973], and siAR3 [HSS100972]), along with the validated nontargeting siRNA (Stealth RNAi siRNA Negative Control Med GC Duplex #2), were purchased from Thermo Fisher Scientific (Waltham, MA, USA).

For KRAS knockdown, LNCaP and DU145 cells were seeded in six-well plates and transfected with 20 nM siRNA for 24 h. For AR knockdown, LNCaP cells were seeded in six-well plates and transfected with 10 nM siRNA for 24 h. Total RNA and protein were extracted 24 h after transfection.

### Proliferation assay

The proliferation assay was conducted in six-well culture plates. LNCaP, LNCaP-SF, and DU145 were seeded in six-well culture plates at 10 × 10^4^, 10 × 10^4^, and 5 × 10^4^, respectively. The following day, rhFGF8b, rhEGF, or BI-3406 was added to the medium, depending on the specific experiment. At the end of the culture period, the cells were trypsinized, harvested, and counted using a CellDrop BF (DeNovix, Wilmington, DE, USA).

### Cell migration and invasion assay

The cell migration assay was conducted using 8.0-μm pore transwell inserts, and the cell invasion assay was performed with 8.0-μm pore transwell inserts coated with 100 μl of Matrigel (Corning, Corning, NY, USA) in 24-well culture plates. The Matrigel matrix was diluted to 2 mg/ml using FBS-free medium. Prostate cancer cells were cultured to 80% confluency in the appropriate medium. The upper chamber contained 8 × 10^4^ cells for LNCaP and LNCaP-SF in 300 μl RPMI-1640 medium supplemented with 2% FBS for LNCaP and 2% CCS for LNCaP-SF, and 3 × 10^4^ cells for DU145 in 300 μl (200 μl in the invasion assay) of 5% FBS in DMEM medium. The lower chamber was filled with 700 μl of RPMI-1640 medium supplemented with 5% FBS for LNCaP, 5% CCS in RPMI-1640 medium for LNCaP-SF, and 5% FBS in DMEM medium for DU145. rhFGF8b, rhEGF, or BI-3406 was added at the specified concentrations.

The cells were incubated at 37 °C with 5% CO_2_ for 24, 36, or 48 h. The cells on the upper surface of the transwell filter were gently removed using a cotton swab soaked in PBS. The cells on the lower surface were then fixed in 4% paraformaldehyde for 10 min, stained with 0.1% crystal violet for 20 min, and photographed. The migrated cells in two random fields were counted manually.

### RNA extraction and PCR

Total RNA was extracted using the Direct-zol™ RNA Miniprep Kit (Zymo Research, Irvine, CA, USA), following the manufacturer’s instructions. RNA concentration was measured with a NanoDrop spectrophotometer (Thermo Fisher Scientific). Complementary DNA (cDNA) was synthesized using the iScript cDNA Synthesis Kit (Bio-Rad, Hercules, CA, USA). Quantitative real-time PCR (qPCR) was performed using the CFX Connect™ Real-Time System (Bio-Rad) with the SsoAdvanced Universal SYBR Green Supermix (Bio-Rad) to measure gene expression. Each gene expression was normalized to GAPDH. Primers for UBE2L3-KRAS were designed based on a previous study [20]. and are listed in Table S2.

### Western blot (WB) analysis

Cell lysates were prepared using RIPA buffer (Fujifilm, Tokyo, Japan) with protease and phosphatase inhibitor cocktails (1%) (P0044 and P8340; Sigma-Aldrich, St. Louis, MO, USA). The soluble lysates were mixed with a sample buffer containing lithium dodecyl sulfate and a reducing agent (both from Thermo Fisher Scientific) and were separated by SDS-PAGE. Proteins were transferred to nitrocellulose membranes, which were blocked with 1% gelatin in 0.05% Tween in Tris-buffered saline (TBST) for 1 h at room temperature. For FGF8b, membranes were blocked with 5% skimmed milk in TBST. The membranes were incubated overnight at 4°C with primary antibodies diluted in 3% BSA and 0.2% gelatin in TBST, while FGF8b was diluted in 5% skimmed milk in TBST. Primary antibodies were used at 1:1000 dilution, and GAPDH at 1:20,000. After three washes, membranes were incubated with HRP-conjugated anti-rabbit (1:1000) or anti-mouse secondary antibodies (1:20000) for 1 h at room temperature. Protein bands were detected using Clarity™ Western ECL Substrate (Bio-Rad). Chemiluminescence signals were captured using the ChemiDoc™ XRS+ System (Bio-Rad), and the resulting images were analyzed using Image Lab™ software (Bio-Rad).

### Enzyme-linked immunosorbent assay (ELISA)

ELISA was conducted using six-well culture plates. The medium of LNCaP and DU145 cells was replaced with 5% FBS in RPMI-1640 or DMEM medium, respectively, while LNCaP-SF cells were cultured in 5% CCS in RPMI-1640 medium. The medium of the prostate cancer-associated stromal cell line was replaced with 10% FBS or 5% CCS in RPMI-1640 medium. After 96 h, the supernatant was collected for analysis, and cells were counted at the time of collection. Human FGF8b or EGF secretion was quantified using Human FGF8b ELISA Kit (GR111226-1; Genorise, Glen Mills, PA, USA), or Human EGF ELISA Kit (DEG00; R&D Systems), following the manufacturer’s instructions. Absorbance was measured at 450 nm and corrected at 540 nm using a microplate reader.

### Immunohistochemistry (IHC)

For each specimen, slides were prepared from paraffin blocks containing the most abundant tumor tissue. Sections (5 μm thick) were cut and subsequently deparaffinized. Antigen retrieval was performed by immersing the slides in 20 mM Tris–HCl buffer (pH 9.0), heating at 95 °C for 20 min (sub-boiling), and allowing them to cool at room temperature for 1 h. Endogenous peroxidase activity was quenched with 0.3% hydrogen peroxide in methanol for 10 min. To block nonspecific binding, slides were incubated with 3% bovine serum albumin (BSA) in PBS for 1 h at room temperature. Slides were then incubated with primary antibodies overnight at 4 °C. After three washes with PBS, HRP-conjugated secondary antibodies were applied and incubated for 1 h. The primary antibodies were used at the following dilutions: AR (1:300), PSA (1:1000), synaptophysin (1:2000), chromogranin A (1:1000), p-ERK (1:100), p-p38 (1:100), p-AKT (1:100), Ki-67 (1:100), cleaved-caspase3 (1:50), BCL-xL (1:300) and CD56 (pre-diluted). The secondary antibodies were used at the following dilutions: HRP-conjugated anti-rabbit IgG antibody (1:300) and HRP-conjugated anti-mouse IgG antibody (1:300). This experiment was performed only once due to the limited availability of tissue samples.

Signal development was carried out using the ImmPACT DAB EqV peroxidase substrate (Vector Laboratories, CA, USA), followed by counterstaining with hematoxylin. Slides were examined under 40× magnification, and the percentage of positively stained cells was calculated.

### Cell co-culture experiments

Cell co-culture experiments were conducted using a 1.0-μm pore transwell in six-well culture plates. Prostate cancer-associated stromal cells were seeded in the upper chamber, and prostate cancer cells were seeded in the lower chamber, with both cell types plated to achieve 80–100% confluence by the time of collection. The reverse co-culture, with prostate cells in the upper chamber and prostate cancer-associated stromal cells in the lower chamber, was also set up in the same manner. Co-culture was initiated the following day, and WB analysis and ELISA were performed according to the experimental protocol.

### Neutralization assay

In the co-culture experiment, culture supernatants were supplemented with an anti-FGF8 neutralizing antibody at a final concentration of 1 ng/mL. After 24 h of incubation, the cell lysates were subjected to WB analysis.

### Xenograft study in mice

A total of 13 male severe combined immunodeficiency (SCID) mice, 6 weeks old, were obtained from Clea Japan (Tokyo, Japan). After a 1-week acclimatization period, 3 × 10^6^ DU145 cells mixed with 50% Matrigel (Corning) were implanted subcutaneously into the mice. Once the tumors reached a visible size, the mice were divided into two groups: a control group and a treatment group. Mice in the treatment group were given BI-3406 at 30 mg/kg, while the control group received a vehicle (20 μL DMSO + 80 μL corn oil) orally once daily. The 30 mg/kg dose was prepared by dissolving BI-3406 in 20 μL of DMSO and adding 80 μL of corn oil to make a total volume of 100 μL. Tumor size and body weight were measured every 2–3 days using the formula (length × width × width)×0.5. After 24 days of treatment, the tumors were excised and immunostained. This experiment was performed only once due to ethical considerations. No randomization or blinding was performed.

### Cancer genomic profiling

Out of 30 CRPC patients, 25 were profiled using the FoundationOne®CDx Cancer Genomic Profile (Foundation Medicine, Cambridge, MA, USA), and 5 were profiled using the OncoGuide NCC Oncopanel System (Sysmex, Kobe, Japan), developed in Japan. The research using these comprehensive cancer genomic profiling data from prostate cancer patients was approved by the Medical Ethics Committee of Kanazawa University (Approval Number: 2020-286).

### Statistical analysis

Data are presented as the mean ± standard error of the mean (SEM). Statistical analysis was conducted using GraphPad Prism 9 (GraphPad Software, Boston, MA, USA). Sample sizes for in vitro experiments (*n* = 3 or 4 per group) and in vivo experiments (Control: *n* = 7, Treatment: *n* = 6) were based on standard practice and previous studies. No formal statistical power calculation was performed. Differences between the two groups were analyzed using an unpaired two-sided Student’s *t*-test. All experiments were performed in triplicate unless otherwise noted. Data distribution and variance were visually inspected to confirm that the assumptions of each statistical test were reasonably met. Statistical significance was defined as *p < 0.05, **p < 0.01, ***p < 0.001, and ****p < 0.0001.

## Results

### Contribution of KRAS signaling to AR-independent prostate cancer progression

**Fig. 1:**
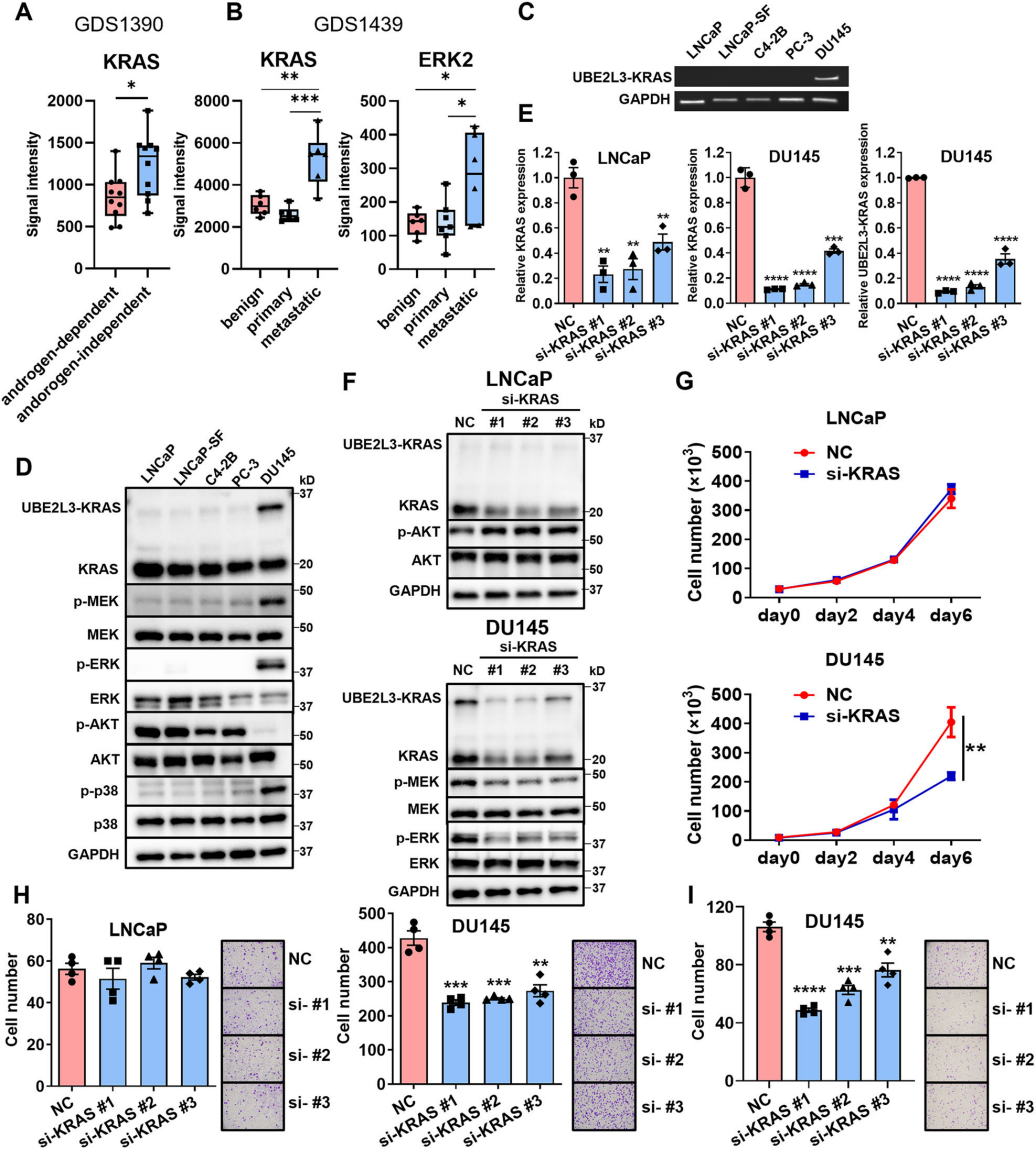
KRAS signaling promotes AR-independent CRPC progression. **A** GDS1390 data indicated increased KRAS expression in androgen-independent prostate cancer. **B** GDS1439 data showed higher expression of both KRAS and ERK2 (a downstream target of KRAS) at metastatic sites. **C** RT-PCR analysis of UBE2L3-KRAS expression in various prostate cancer cell lines. Primers were designed using a combination of KRAS_q2 and UBE2L3_q1 (refer to Fig. S2). **D** WB analysis of KRAS downstream signaling in prostate cancer cell lines. **E** KRAS expression levels in LNCaP cells and both KRAS and UBE2L3-KRAS expression levels in DU145 cells after KRAS knockdown. Cells were transfected with KRAS-specific siRNA (si-KRAS#1, si-KRAS#2, si-KRAS#3) or a negative control from three independent experiments. **F** WB analysis of KRAS downstream signaling in LNCaP and DU145 cells following KRAS knockdown. **G** Proliferation assay of LNCaP and DU145 cells after KRAS knockdown using si-KRAS#2 (*n* = 4/group). **H** Migration assay of LNCaP and DU145 cells after KRAS knockdown (*n* = 4/group). **I** Invasion assay of DU145 cells after KRAS knockdown (*n* = 4/group). *p < 0.05; **p < 0.01; ***p < 0.001; ****p < 0.0001.

The clinical database revealed activation of KRAS signaling in both AR-independent and metastatic prostate cancer (Fig. 1A and B). Because FGF has previously been reported to be associated with DNPC [12]. We predicted that KRAS is downstream of FGF and that the activation of KRAS would be involved in the progression of DNPC.

### Promotion of AR-independent CRPC progression by KRAS signaling

It has been previously reported that DU145, a human prostate cancer cell line, has a KRAS gene fusion with the ubiquitin-conjugating enzyme UBE2L3 (UBE2L3-KRAS) [20]. Additionally, DU145 exhibits complete AR independence without any NED characteristics. Thus, we considered DU145 to be a DNPC-like cell line. Using reverse transcription (RT)-PCR, we confirmed that UBE2L3-KRAS was present only in DU145 among the examined human prostate cancer cell lines (Fig. 1C). LNCaP-SF is an androgen-insensitive cell line derived from LNCaP [17]. Among the six primer pairs we prepared for UBE2L3-KRAS, the combination of UBE2L3_q1 and KRAS_q2 was the most effective (Fig. S1). WB analysis revealed increased KRAS downstream signaling—MEK, ERK, and p38—only in DU145, but not in other cell lines without KRAS mutations, despite high KRAS levels. In contrast, prostate cancer cell lines other than DU145 showed elevated AKT signaling (Fig. 1D). To investigate the role of KRAS in AR-dependent and AR-independent prostate cancer cells, KRAS expression was knocked down in LNCaP and DU145 cells using three different small siRNAs. KRAS messenger RNA (mRNA) and protein levels in siKRAS1, siKRAS2, and siKRAS3 cells were measured by qPCR and WB analysis. We confirmed that both UBE2L3-KRAS mRNA and KRAS expression could be suppressed in DU145 (Fig. 1E). KRAS knockdown did not affect AKT signaling in LNCaP cells, indicating that AKT is not regulated by KRAS. In DU145 cells, KRAS and UBE2L3-KRAS protein expression were reduced by KRAS knockdown, leading to suppressed MEK and ERK signaling (Fig. 1F). KRAS knockdown suppressed epithelial–mesenchymal transition (EMT) and promoted apoptosis in DU145 cells, as shown by WB analysis, but no changes were observed in LNCaP cells (Fig. S2A). KRAS knockdown significantly inhibited proliferation and migration in DU145 cells but had no effect on LNCaP cells (Figs. 1G, H and S2B). Additionally, KRAS knockdown strongly suppressed the invasion of DU145 cells (Fig. 1I). These findings suggest that KRAS signaling is not active in AR-dependent prostate cancer cells but plays a role in DNPC-like DU145 cells.

### FGF- and EGF-induced cancer progression in AR-independent CRPC via KRAS activation

**Fig. 2:**
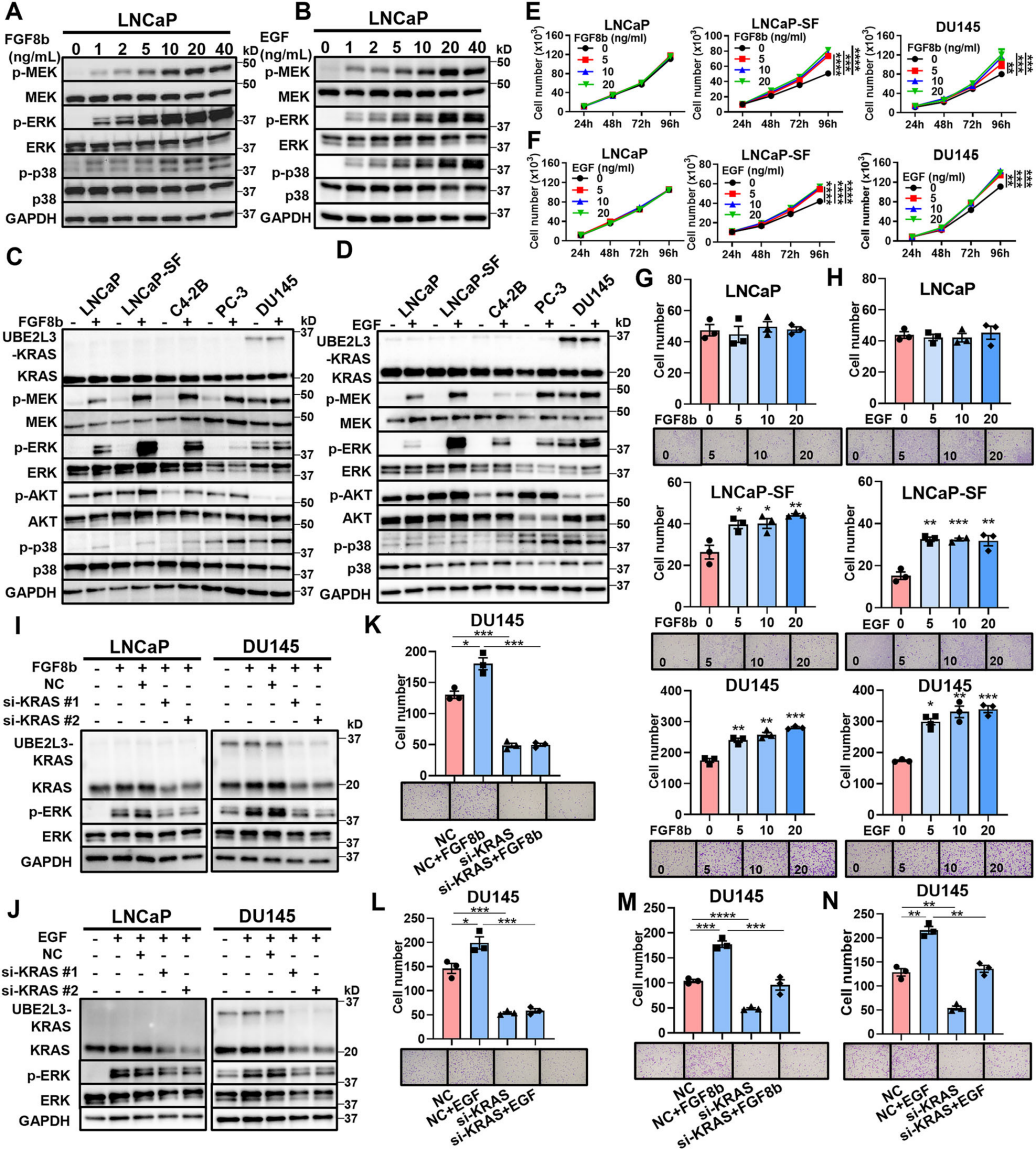
FGF and EGF promote cancer progression in AR-independent CRPC by activating KRAS signaling. WB analysis of KRAS downstream signaling in LNCaP cells treated with various concentrations of FGF8b (**A**) and EGF (**B**). WB analysis of KRAS downstream signaling in different prostate cancer cell line cells with or without FGF8b (5 ng/mL) (**C**) and EGF (5 ng/mL) (**D**). Proliferation assay of LNCaP, LNCaP-SF, and DU145 cells treated with different concentrations of FGF8b (**E**) and EGF (**F**) (*n* = 4/group). Migration assay of LNCaP, LNCaP-SF, and DU145 cells treated with varying concentrations (ng/mL) of FGF8b (**G**) and EGF (**H**) (*n* = 3/group). WB analysis of KRAS downstream signaling in LNCaP and DU145 cells with KRAS knockdown and with FGF8b (5 ng/mL) (**I**) and EGF (5 ng/mL) (**J**) (*n* = 3/group). Migration assay of DU145 cells with KRAS knockdown and FGF8b (5 ng/mL) (**K**) and EGF (5 ng/mL) (**L**) (*n* = 3/group). Invasion assay of DU145 cells with KRAS knockdown and FGF8b (5 ng/mL) (**M**) and EGF (5 ng/mL) (**N**) (*n* = 3/group). *p < 0.05; **p < 0.01; ***p < 0.001; ****p < 0.0001.

Tyrosine kinase receptors, such as FGFR and EGFR, are known to be key upstream activators of KRAS. FGF8b, a ligand for FGFR, has been implicated in DNPC progression [12]. Therefore, FGF8b was considered to be the likely ligand responsible for KRAS activation. Similarly, EGF is also a factor that activates KRAS. FGF8b and EGF increased KRAS downstream signaling in a dose-dependent manner in LNCaP cells (Fig. 2A and B) and also enhanced KRAS signaling in other prostate cancer cells, excluding DU145 (Fig. 2C and D). FGF8b and EGF promoted proliferation and migration in AR-independent LNCaP-SF and DU145 cells but had no effect on AR-dependent LNCaP cells (Fig. 2E–H). FGF8b and EGF also increased invasion in DU145 cells, as confirmed by additional experiments (Fig. S3A and S3B). WB analysis revealed that FGF8b and EGF did not activate ERK, a downstream target of KRAS, in LNCaP and DU145 cells with KRAS knockdown (Fig. 2I and J). KRAS knockdown inhibited the increased migration and invasion of DU145 cells induced by FGF8b and EGF (Fig. 2K–N). These findings suggest that FGF8b and EGF promote AR-independent prostate cancer progression by activating KRAS signaling, but they did not promote functional activation of AR-dependent LNCaP cells despite inducing KRAS downstream signaling.

### Inhibition of CRPC progression by Pan-KRAS inhibitor BI-3406

**Fig. 3:**
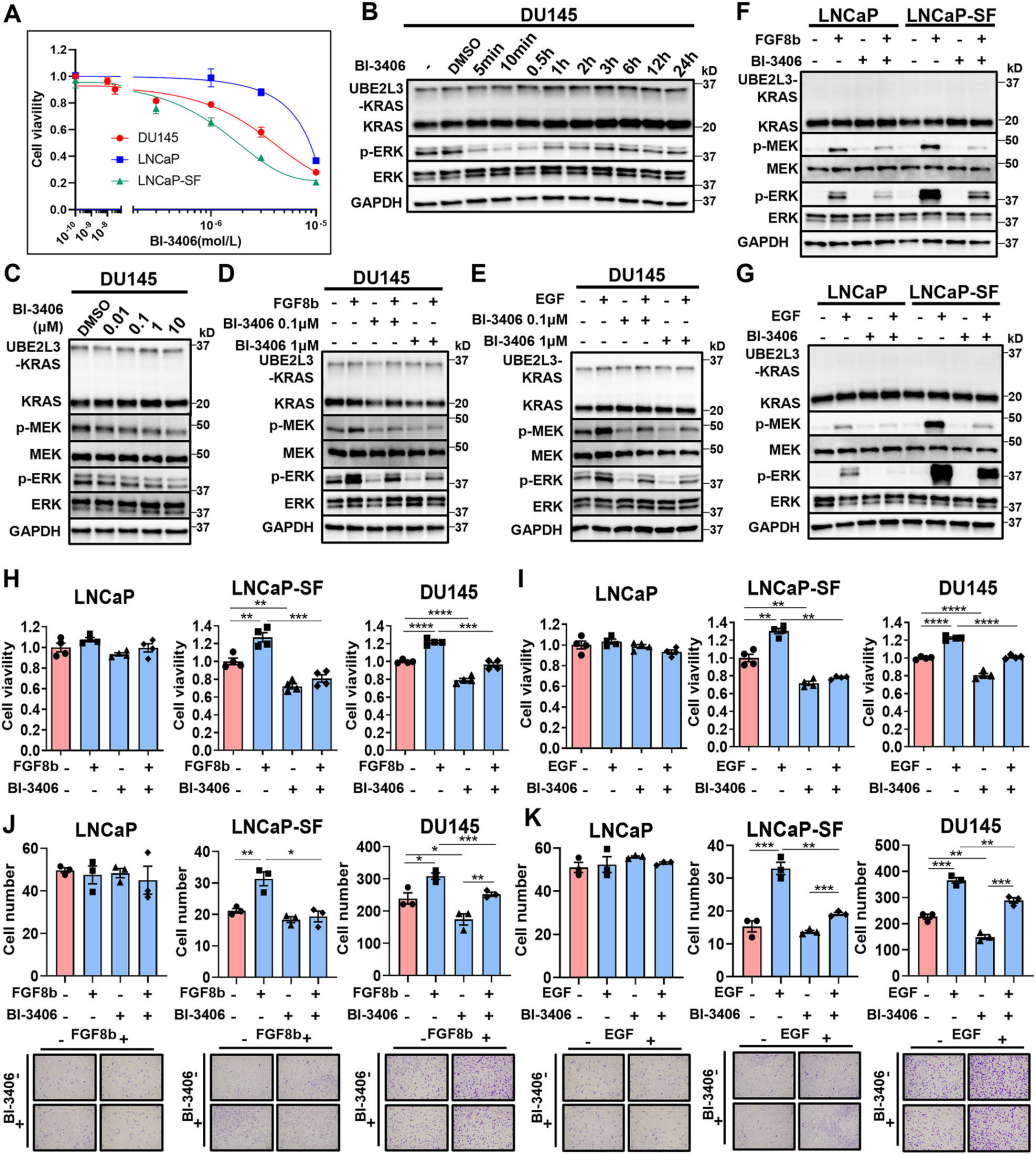
Pan-KRAS inhibitor (BI-3406) inhibits CRPC progression induced by FGFR activation. **A** Cell inhibition curves for BI-3406 in LNCaP, LNCaP-SF, and DU145 cells. The IC_50_ values were 3.8 μM for LNCaP, 1.1 μM for LNCaP-SF, and 1.5 μM for DU145 cells. **B** WB analysis of KRAS downstream signaling in DU145 cells treated with BI-3406 (0.1 μM) over time. **C** WB analysis of KRAS downstream signaling in DU145 cells treated with various concentrations of BI-3406 for 12 h. WB analysis of KRAS downstream signaling in DU145 cells treated with FGF8b (10 ng/mL) and BI-3406 (0.1 μM or 1 μM) (**D**), and EGF (10 ng/mL) and BI-3406 (0.1 μM or 1 μM) (**E**). WB analysis of KRAS downstream signaling in LNCaP and LNCaP-SF cells treated with FGF8b (10 ng/mL) and BI-3406 (0.1 μM) (**F**), and EGF (10 ng/mL) and BI-3406 (0.1 μM) (**G**). Proliferation assay of LNCaP, LNCaP-SF, and DU145 cells treated with FGF8b (10 ng/mL) and BI-3406 (0.1 μM) (**H**), and EGF (10 ng/mL) and BI-3406 (0.1 μM) (**I**) (*n* = 4/group). Migration assay of LNCaP, LNCaP-SF, and DU145 cells treated with FGF8b (10 ng/mL) and BI-3406 (0.1 μM) (**J**), and EGF (10 ng/mL) and BI-3406 (0.1 μM) (**K**) (*n* = 3/group). *p < 0.05; **p < 0.01; ***p < 0.001; ****p < 0.0001.

BI-3406 is a selective inhibitor of the Son of Sevenless 1 (SOS1)-KRAS interaction that effectively blocks KRAS downstream signaling [21]. The proliferation of LNCaP-SF and DU145 cells was more strongly inhibited by BI-3406 compared to LNCaP cells (Fig. 3A). WB analysis showed that BI-3406 suppressed ERK signaling in DU145 cells, with a rapid reduction observed immediately after treatment, though approximately half of ERK signaling was restored after 24 h (Fig. 3B). BI-3406 inhibited MEK-ERK signaling in DU145 cells in a dose-dependent manner (Fig. 3C). BI-3406 also suppressed FGF8b and EGF-induced downstream KRAS signaling in DU145 cells (Fig. 3D and E), with similar results observed in both LNCaP and LNCaP-SF cells (Fig. 3F and G). While BI-3406 suppressed proliferation and migration induced both by FGF8b and EGF in LNCaP-SF and DU145 cells, no changes were seen in LNCaP cells (Fig. 3H–K). Invasion in DU145 cells was also inhibited by BI-3406 (Fig. S4A and S4B). These findings suggest that the pan-KRAS inhibitor BI-3406 may inhibit AR-independent prostate cancer progression by blocking both canonical and abnormal KRAS signaling.

### Altered FGFR expression and enhanced FGF-dependent KRAS signaling in AR-independent prostate cancer

**Fig. 4:**
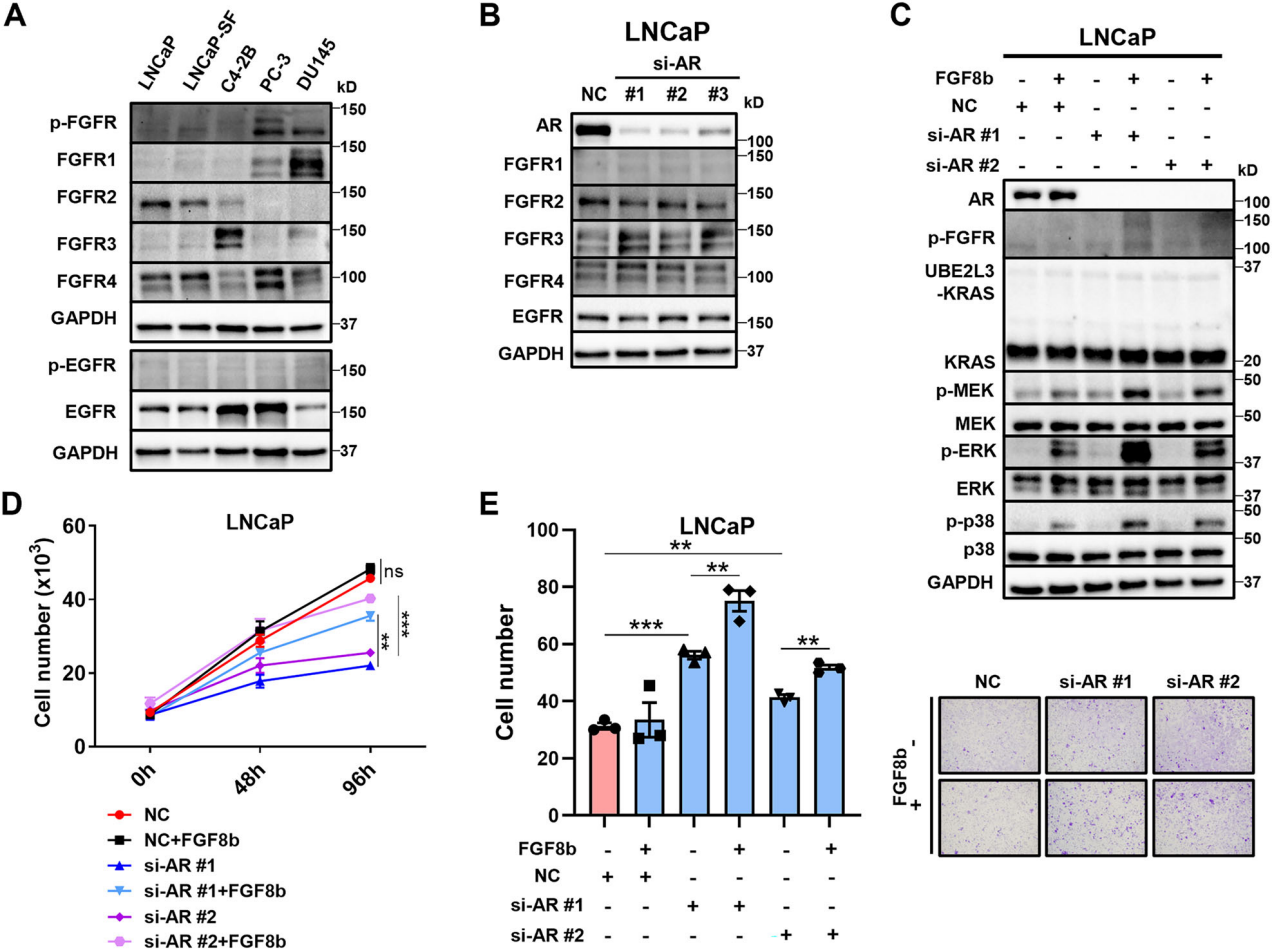
As prostate cancer becomes AR-independent, FGFR expression is altered, and FGF-KRAS signaling is enhanced. **A** WB analysis of FGFR subtypes in various prostate cancer cell line cells. **B** WB analysis of FGFR subtypes in LNCaP cells with AR knockdown. AR-specific siRNAs (si-AR#1, si-AR#2, si-AR#3) and a negative control were transfected into the cells. **C** WB analysis of KRAS downstream signaling in LNCaP cells with AR knockdown and treated with FGF8b (10 ng/mL). **D** Proliferation assay of LNCaP cells with AR knockdown and treated with FGF8b (10 ng/mL) over time (*n* = 3/group). **E** Migration assay of LNCaP cells with AR knockdown and treated with FGF8b (10 ng/mL) (*n* = 3/group). **p < 0.01; ***p < 0.001; ****p < 0.0001.

FGFR consists of four subtypes: FGFR1, FGFR2, FGFR3, and FGFR4, and FGF8b binds to FGFR3 and FGFR4 to transmit its signal, while EGFR has only one subtype [22, 23]. Different prostate cancer cell lines exhibit varying patterns of FGFR and EGFR expression. In LNCaP cells, FGFR2 expression was high, while FGFR3 expression was almost absent (Fig. 4A). AR knockdown in LNCaP cells reduced FGFR2 expression and increased FGFR3 and FGFR4 expression, with no change in EGFR expression (Fig. 4B). This suggests that FGFR2 is positively correlated with AR signaling, while FGFR3 and FGFR4 are negatively correlated. As a result, AR independence activates KRAS downstream signaling through FGF8b–FGFR activation (Fig. 4C). As expected, AR knockdown suppressed the proliferation of LNCaP cells, but FGF8b restored the suppressed proliferation (Fig. 4D). Additionally, FGF8b stimulation under AR knockdown further enhanced migration (Fig. 4E). These results strongly indicate that AR-independent prostate cancer progression is driven by KRAS signaling activation through FGF8b–FGFR signaling.

### Role of FGF8b from prostate cancer-associated stromal cells in AR-independent cancer progression

**Fig. 5:**
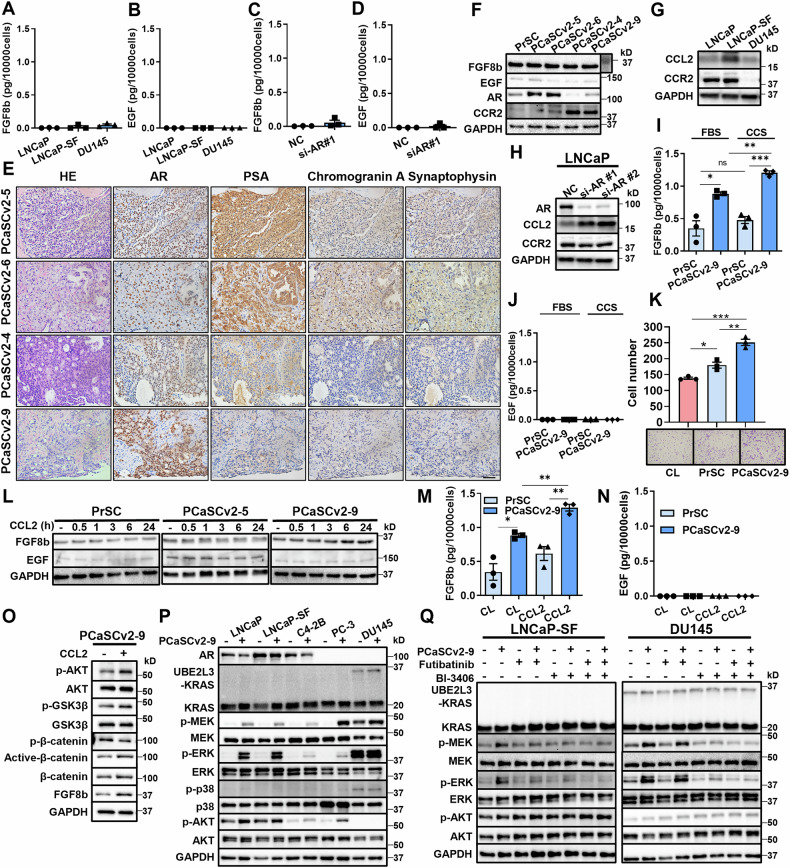
FGF8b secreted by prostate cancer-associated cells promotes AR-independent prostate cancer progression. Measurement of FGF8b (**A**) and EGF (**B**) secreted by LNCaP, LNCaP-SF, and DU145 cells from three different experiments. Cells were cultured in media with 5% FBS (LNCaP and DU145) or 5% CCS (LNCaP-SF) for 96 h, after which the media were collected and analyzed by ELISA. Measurement of FGF8b (**C**) and EGF (**D**) levels secreted by LNCaP cells with AR knockdown from three different experiments. Cells were cultured in media with 5% FBS for 96 h, and the media were collected and analyzed by ELISA. **E** IHC staining of prostate tissue was used to establish prostate cancer-associated cell lines. Scale bar = 50 µm. **F** WB analysis of FGF8b, EGF, AR, and CCR2 in various prostate cancer-associated stromal cells. **G** WB analysis of CCL2 and CCR2 in prostate cancer cells. **H** WB analysis of CCL2 and CCR2 in LNCaP cells with AR knockdown. Quantification of FGF8b (**I**) and EGF (**J**) secreted by PrSC and PCaSCv2-9 cells. Cells were cultured in medium containing 10% FBS or 5% CCS for 96 h, and the media were collected for ELISA (*n* = 3/group). **K** Migration assay of DU145 cells co-cultured with PrSC or PCaSCv2-9 cells (*n* = 3/group). **L** WB analysis of FGF8b and EGF expression in the presence of CCL2 (10 ng/mL) over time. Quantification of FGF8b (**M**) and EGF (**N**) secretion by PrSC and PCaSCv2-9 cells from three different experiments. Cells were cultured in medium containing 10% FBS and CCL2 (10 ng/mL) for 96 h, and the media were collected for ELISA. **O** WB analysis of CCR2 downstream signaling in PCaSCv2-9 cells. Cells were cultured in medium containing 5% CCS and CCL2 (10 ng/mL) for 24 h. **P** WB analysis of KRAS downstream signaling in prostate cancer cell lines co-cultured with PCaSCv2-9 cells for 48 h. **Q** WB analysis of KRAS downstream signaling in LNCaP-SF and DU145 cells co-cultured with PCaSCv2-9 cells and treated with futibatinib (10 nM) or BI-3406 (0.1 μM). *p < 0.05; **p < 0.01; ***p < 0.001.

In the absence of KRAS abnormalities, prostate cancer cells rely on factors such as FGFs and EGF to activate KRAS in the tumor microenvironment. ELISA measurements of autocrine FGF8b secretion from LNCaP, LNCaP-SF, and DU145 cells revealed only low levels of secretion (Fig. 5A). No EGF secretion was detected in prostate cancer cells (Fig. 5B). AR knockdown did not increase the secretion of either FGF8b or EGF from LNCaP cells (Fig. 5C and D).

Due to the lack of FGF8b and EGF secretion from prostate cancer cells, we hypothesized that KRAS-activating factors might be secreted by prostate cancer-associated stromal cells in the primary prostate cancer microenvironment. To test this, we used the human prostate stromal cell line PrSC and patient-derived prostate cancer-associated stromal cells from prostate biopsy samples, referred to as PCaSCv2s. PCaSCv2-5 and PCaSCv2-6 were derived from castration-sensitive prostate cancer (CSPC) patients with metastases, while PCaSCv2-4 and PCaSCv2-9 were derived from CRPC patients. The patient who provided PCaSCv2-9 showed DNPC characteristics, including low PSA, neuron-specific enolase (NSE), and pro-gastrin-releasing peptide (ProGRP) levels at the time of local recurrence, with IHC staining also negative for PSA, chromogranin A, and synaptophysin (Fig. 5E). A detailed description of the PCaSCv2 lines is provided in Fig. S5. All prostate cancer-associated stromal cells showed high FGF8b and low EGF levels in WB analysis. PCaSCv2-5 and PCaSCv2-6, derived from CSPC patients, had high AR levels, whereas PCaSCv2-4 and PCaSCv2-9, from CRPC patients, had low AR levels (Fig. 5F). WB analysis showed increased CCL2 expression in LNCaP-SF cells compared with LNCaP cells (Fig. 5G), and AR knockdown in LNCaP cells further enhanced CCL2 expression (Fig. 5H). Previous studies also showed that increased CCL2 secretion from AR knockdown prostate cancer cells induces treatment resistance in an autocrine manner [24–26]. These findings suggest that high levels of CCL2 could influence both prostate cancer cells and prostate cancer-associated stromal cells in the primary tumor microenvironment. We next examined whether CCL2 influences KRAS signaling in prostate cancer cells. In contrast to FGF8b and EGF, treatment with CCL2, even in a dose-dependent manner, did not induce activation of KRAS-related signaling pathways in prostate cancer cells (Fig. S6). Subsequently, we investigated prostate cancer-associated stromal cells. Notably, the expression of CCR2, the receptor for CCL2, was elevated in PCaSCv2-4 and PCaSCv2-9 cells, whereas the expression levels of other chemokine receptors remained unchanged in these stromal cell lines (Figs. 5F and S7A). We next evaluated whether CCL2 secreted by prostate cancer cells alters CCL2 expression in cancer-associated stromal cells (PCaSCv2-9); however, no change was observed (Fig. S7B). These findings suggest that suppression of AR signaling leads to upregulation of the CCL2 receptor CCR2 in prostate cancer-associated stromal cells, thereby enhancing their responsiveness to CCL2, even when the secretion levels of CCL2 from prostate cancer cells remain unchanged.

ELISA results showed that FGF8b secretion was higher in PCaSCv2-9 cells compared to PrSC (Fig. 5I). EGF, however, was not secreted by either PrSC or PCaSCv2-9 cells (Fig. 5J). In co-culture experiments, PCaSCv2-9 cells promoted the migration and invasion of DU145 cells more than PrSC cells (Figs. 5K and S7C). Stimulation of prostate cancer cells with CCL2 did not increase FGF8b or EGF secretion in ELISA (Figs. S7D and S7E). However, CCL2 stimulation enhanced FGF8b expression in PCaSCv2-9 cells, without affecting EGF expression in WB analysis (Fig. 5L). Furthermore, the CCL2-induced increase in FGF8b expression in PCaSCv2-9 cells was attenuated by treatment with a CCR2 antagonist (RS102895, 10 μM, 24 h) in WB analysis, suggesting the involvement of CCR2 signaling in this regulatory process (Fig. S7F). CCL2 stimulation increased FGF8b secretion from PCaSCv2-9 cells, while FGF8b secretion from PrSC was only slightly elevated in FBS-containing medium (Fig. 5M). FGF8b secretion from PCaSCv2-9 cells was further enhanced in CCS-containing medium (Fig. S7G). EGF was not secreted by either PrSC or PCaSCv2-9 cells in FBS or CCS-containing medium, even under the same conditions used for FGF8b experiments (Figs. 5N and S7H). Next, we investigated the signaling pathway linking CCL2 stimulation to FGF8b expression in prostate cancer–associated stromal cells. CCL2 stimulation of PCaSCv2-9 cells promoted phosphorylation of AKT and GSK3β, accompanied by increased expression of active β-catenin in WB analysis (Fig. 5O). Moreover, WB analysis demonstrated that treatment with a β-catenin inhibitor XAV-939 (10 μM, 24 h) suppressed the CCL2-induced upregulation of FGF8b expression (Fig. S7I).

WB analysis showed that co-culturing with PCaSCv2-9 cells reduced AR expression in prostate cancer cell lines and increased downstream KRAS signaling (MEK-ERK), similar to the effect seen with recombinant FGF8b stimulation (Fig. 5P). In LNCaP-SF cells, both futibatinib [27]. An oral FGFR1–4 inhibitor and BI-3406 suppressed MEK-ERK signaling, with the combination of the two providing a stronger effect. In contrast, in DU145 cells, futibatinib did not suppress MEK-ERK signaling, but both BI-3406 and the combination of the two compounds did (Fig. 5Q). Neither futibatinib nor BI-3406 affected AKT signaling (Fig. 5Q). Furthermore, we confirmed that the activation of KRAS-related signaling pathways in prostate cancer cells, which was induced by co-culture with stromal cells (PCaSCv2-9), was attenuated by treatment with a FGFR3 inhibitor TYRA-300 (50 nM, 2 h) [28]. and a FGF8b-neutralizing antibody (1 ng/mL) (Figs. S8A and S8B). These results suggest that CCL2 secreted from AR-independent prostate cancer cells stimulates prostate cancer-associated stromal cells to release FGF8b, which then activates AR-independent prostate cancer cells.

### Suppression of prostate cancer cell growth in a xenograft mouse model by BI-3406

**Fig. 6:**
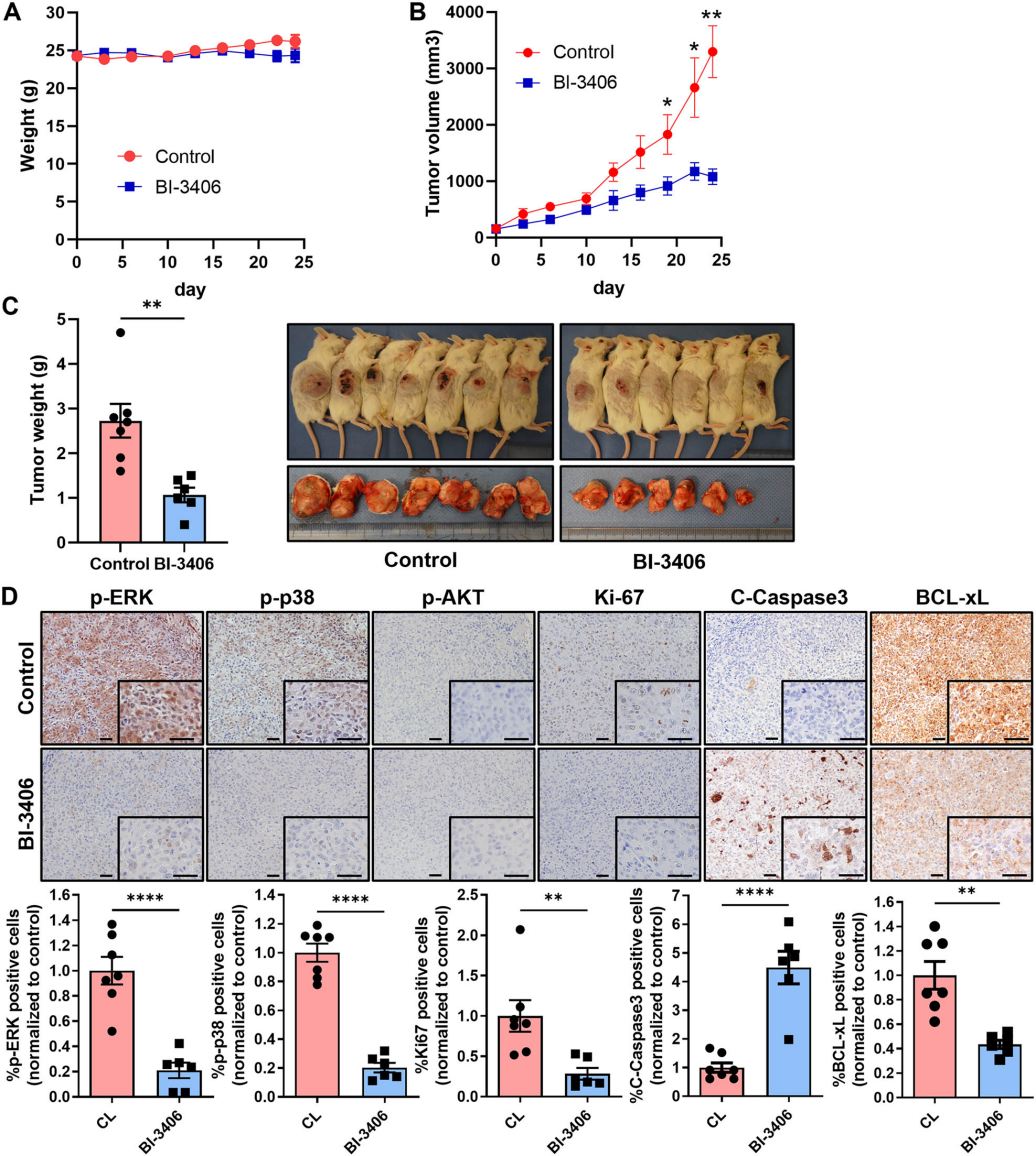
BI-3406 suppresses prostate cancer cell growth in a xenograft mouse model. DU145 cells were subcutaneously injected into SCID mice, and when the average tumor size reached 150 mm^3^, BI-3406 was administered orally once daily at a dose of 30 mg/kg. Body weights and tumor sizes were measured every 2–3 days. On day 24, the mice were sacrificed, and the tumors were excised 1 h after the final BI-3406 dose. Data are presented as mean ± SEM (Control: *n* = 7, Treatment: *n* = 6). **A** Change in body weight over time. **B** Change in tumor volume over time. **C** Tumor weight at sacrifice. **D** IHC staining of p-ERK, p-p38, ki-67, Cleaved-Caspase 3, and BCL-xL in excised tumors. Statistical analysis was performed for all markers, except p-AKT, as no expression of p-AKT was detected. Scale bars = 50 µm.

DU145 cells were injected subcutaneously into severe combined SCID mice to evaluate the effect of BI-3406. The mice were divided into control and BI-3406 treatment groups. There was no significant difference in body weight between the two groups (Fig. 6A). Tumor growth was notably reduced in the treatment group compared with the control group (Fig. 6B). On day 24 of treatment, 1 h after administering BI-3406, the mice were sacrificed, and the tumors were excised. The tumors from the treatment group were significantly smaller in weight than those from the control group (Fig. 6C). IHC of the tumors showed that treatment with BI-3406 resulted in decreased expression of p-ERK, p-p38, Ki-67, and BCL-xL, along with increased expression of cleaved caspase-3 (Fig. 6D). These findings demonstrate that BI-3406 inhibited KRAS signaling and suppressed DU145 cell growth in vivo.

### Comprehensive cancer genomic profiles show KRAS gene abnormalities in DNPC patients

**Fig. 7:**
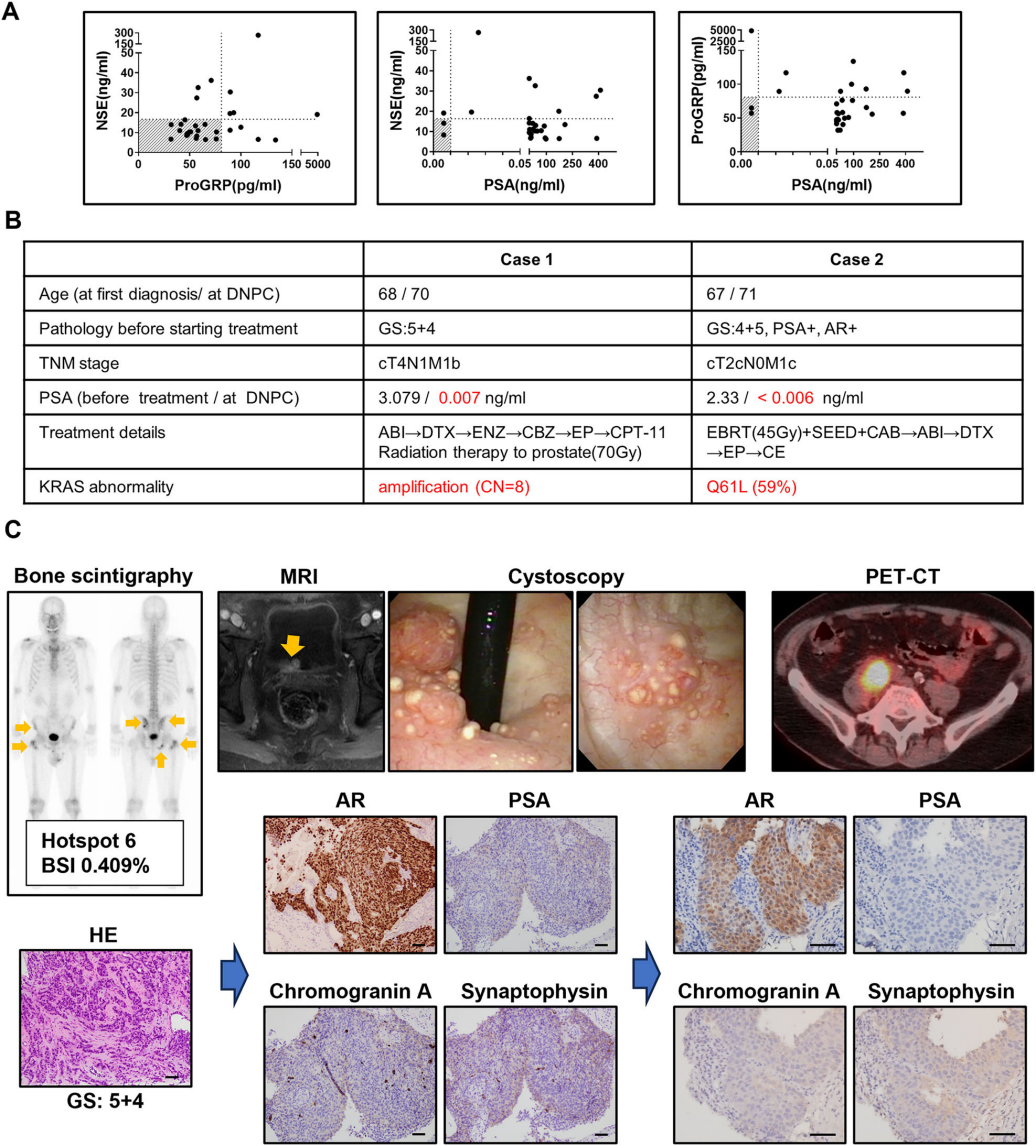
Clinicopathological features of DNPC patients. **A** PSA, NSE, and ProGRP levels at recurrence in CRPC patients who underwent comprehensive cancer genomic profiling. The shaded regions in each panel represent the negative (normal) ranges. **B** Characteristics of two DNPC patients with detected KRAS genetic abnormalities. **C** Examination findings of Case 1. Left panels: Bone scintigraphy (top) at first diagnosis and HE staining of the prostate (bottom) at the initial biopsy. Arrows indicate bone metastases. Middle panels: Magnetic resonance imaging and endoscopy (top), and IHC staining of AR, PSA, chromogranin A, and synaptophysin of the bladder neck tumor (bottom) at the first recurrence. Prostate cancer had locally invaded the bladder, with IHC staining of the recurrent tissue showing positivity for AR and negativity for PSA, chromogranin A, and synaptophysin. Arrows indicate local invasion into the bladder. Right panels: a positron emission tomography-computed tomography (top) and IHC staining of AR, PSA, chromogranin A, and synaptophysin in the iliopsoas lymph node tissue (bottom) at the second recurrence. IHC staining was positive for AR but negative for others, indicating the characteristics of DNPC. Scale bars = 50 µm. ABI abiraterone, ENZ enzalutamide, DTX docetaxel, CBZ, cabazitaxel, EP cisplatin + etoposide, CPT-11 irinotecan, CE carboplatin + etoposide, BSI bone scan index.

To identify potential drivers of DNPC progression, comprehensive cancer genomic profiling was conducted in a cohort of 30 prostate cancer patients. Of these, two patients exhibited complete DNPC, characterized by negative PSA and NED markers, including NSE and ProGRP (Fig. 7A). Both patients shared KRAS gene abnormalities, implicating KRAS as a potential signaling pathway involved in DNPC (Fig. 7B). These cases were marked by a Gleason score (GS) 5 component and relatively low pretreatment PSA levels. Case 1 had bone metastases before treatment. Despite treatment with abiraterone, the patient experienced urethral and bladder invasion from the primary prostate lesion, followed by iliopsoas lymph node metastasis. Prostate cancer with bladder invasion was surgically removed and diagnosed as DNPC, and comprehensive cancer genomic profiling was performed on the tissue samples. IHC of the recurrent tissues showed positivity for AR but negativity for PSA, chromogranin A, and synaptophysin (Fig. 7C). Despite intensive treatment with enzalutamide and cytotoxic chemotherapy, the patient died 3 years and 5 months after the onset of DNPC. In Case 2, there were no metastases at the start of treatment. IHC of the pre-treatment prostate tissue was positive for AR and PSA and negative for chromogranin A and synaptophysin (Fig. S9A). The patient later developed cerebellar metastasis during combined androgen blockade therapy following radiotherapy. The brain tumor was surgically removed and subjected to comprehensive cancer genomic profiling. IHC showed weak positivity for AR and negativity for PSA, chromogranin A, and synaptophysin. KRAS downstream signaling was positive for p-ERK, weakly positive for p-p38, and negative for p-AKT (Fig. S9B). Later, lung metastasis developed during abiraterone treatment, and biopsy confirmed DNPC. IHC of the metastatic tissue was negative for AR, PSA, chromogranin A, and synaptophysin (Fig. S9C). Despite several treatments with cytotoxic chemotherapy agents, the patient died 4 years and 9 months after the onset of DNPC. Both patients developed multiple metastases, including liver metastases. These findings suggest KRAS activation as a potential driver of DNPC. Details of the remaining 28 cases are provided in Tables S3 and S4 and Figs. S10 and S11.

## Discussion

Prostate cancer recurrence after androgen deprivation therapy is typically caused by the activation of AR signaling through mutations, amplifications, or increased sensitivity to androgens, leading to a rise in the tumor marker PSA. In some cases, treatment-induced transformation to NED occurs [29]. with an increase in NED markers such as NSE and ProGRP. These elevated markers aid in the early detection of recurrence as NED. Recently, however, some recurrences have been identified through radiographic imaging, such as computed tomography, even when PSA or NED markers remain unchanged. These cases are referred to as DNPC [8]. DNPC usually has a very poor prognosis [10, 12, 30] and no effective treatment is currently available. While the recent use of novel ARSIs has improved survival outcomes in CRPC, it has inadvertently led to the emergence of another problematic condition, DNPC [12]. Although FGFR [12, 13] and CCL2 [15] have been implicated in DNPC, no clear connection between them has been established so far.

In this study, we focused on KRAS as a potential driver of DNPC based on comprehensive cancer genomic profiling of CRPC patients. RAS family proteins relay signals from growth factor receptors to various effector pathways, promoting cell growth and proliferation [31]. While KRAS mutations are rare in prostate cancer, they are well-known cancer-related genes in other cancers, such as lung, colon, and pancreatic cancers [32, 33]. KRAS mutations lead to a permanently active GTP-bound form, but even wild-type KRAS can be activated by upstream factors like FGFs and EGF, becoming GTP-bound KRAS. Studies have shown that the activation of wild-type KRAS is associated with an increased risk of bone metastasis in prostate cancer [34].

Clinically, DNPC presents heterogeneously, and there are no well-defined criteria beyond being AR-independent and lacking NED features [35]. In this study, we employed DU145 as a model of DNPC-like disease; however, it should be noted that DU145 does not fully recapitulate the DNPC phenotype. We considered DU145 to be the most suitable model for DNPC-like disease, as it is AR-negative and NED-negative, and displays aggressive malignant behavior. Based on our clinical data, we aimed to investigate the role of KRAS in DNPC cells. DU145 harbors a KRAS mutation and constitutively activates the KRAS signaling pathway, making it a relevant model for our experiments.

Among the FGFs, FGF8b has been linked to prostate cancer progression [36–38] and DNPC [12]. DU145 cells harbor a KRAS mutation, UBE2L3-KRAS [20]. which activates KRAS downstream signaling through MEK, ERK, and p38. DU145 is an AR-independent cell line with DNPC-like characteristics. We used DU145 cells to explore the role of KRAS signaling in cancer cell activation. KRAS knockdown suppressed cancer progression in DU145 cells but had no effect in LNCaP cells. This suggests that enhanced KRAS signaling does not contribute to cancer progression in LNCaP, making KRAS inhibition ineffective in the AR-dependent phase. In AR-independent cell lines LNCaP-SF and DU145, FGF8b and EGF enhanced KRAS signaling to drive cancer progression. DU145 cells with the KRAS mutation showed increased cancer progression when stimulated with FGF8b and EGF. This indicates that AR-independent prostate cancer cells rely on KRAS signaling and promote cancer progression through FGFs and EGF, regardless of KRAS mutation status.

FGFR2 expression does not contribute to prostate cancer progression [39, 40]. while FGFR3 expression is increased in CRPC [41]. FGF8b preferentially binds to FGFR3>FGFR4>FGFR2>FGFR1 [23, 42]. AR knockdown in LNCaP cells reduced FGFR2 expression and increased FGFR3 and FGFR4 expression, with FGFR3 showing the most significant increase. In LNCaP cells with AR knockdown, FGF8b enhanced proliferation and migration even in the presence of FBS, suggesting that AR suppression promotes FGFR signaling activation by FGF8b. However, AR knockdown did not affect EGFR expression. These results suggest that as prostate cancer becomes AR-independent, FGFR3 expression increases and KRAS signaling is further activated by FGF8b. Indeed, in AR-independent DNPC patients, FGFR3, the FGFR pathway, and the MEK signature are elevated in prostate cancer tissues [43]. In addition to FGFR and EGFR, other receptor tyrosine kinases such as c-MET and HER2 may also contribute to KRAS activation in advanced prostate cancer. C-MET is generally not considered a primary or frequent activator of KRAS compared to EGFR [44]. Furthermore, studies have shown that c-MET expression is reduced within tumor regions but relatively elevated in peri-tumoral areas in prostate cancer, implying a limited contribution of c-MET to KRAS pathway activation in prostate cancer cells [45]. HER2, meanwhile, has often been reported as a potent activator of KRAS; however, the primary downstream signaling pathway of HER2 is generally thought to be independent of KRAS, involving the PI3K/AKT axis [46].

We then tested a KRAS inhibitor. Recently, the development of KRAS inhibitors, once considered difficult to achieve, has made significant progress, and they are likely to be used for various types of cancers in the future. However, most of these inhibitors target specific genetic mutations. For example, sotorasib is effective against cancers with the KRAS^G12C^ mutation [47]. In this study, we suggest that, in addition to KRAS gene mutations, the activation of wild-type-KRAS signaling through stimulation by FGFs or EGF contributes to the progression of AR-independent prostate cancer. We used BI-3406, a pan-KRAS signaling inhibitor that is effective regardless of KRAS gene abnormalities. BI-3406 is an oral SOS1 inhibitor that prevents KRAS from becoming active (GTP-bound) [21]. SOS1 is a key RAS guanine nucleotide exchange factor that activates KRAS in response to growth factor receptor signals. BI-3406 has been shown to inhibit the progression of KRAS mutant cancers, regardless of the specific mutation. In our study, BI-3406 inhibited growth at lower concentrations in AR-independent LNCaP-SF and DU145 cells compared to LNCaP. It also suppressed MEK-ERK and p38 signaling activated by FGF8b and EGF in LNCaP-SF and DU145 cells. In LNCaP cells, BI-3406 suppressed FGF8b- and EGF-activated KRAS signaling but had little effect on cancer progression, similar to KRAS knockdown. In vivo, BI-3406 treatment reduced pro-survival signaling, including downregulation of BCL-xL expression, and increased apoptotic signaling as evidenced by elevated levels of cleaved caspase-3 in DU145 xenograft tumors, supporting the functional relevance of KRAS inhibition in AR-independent prostate cancer.

KRAS activation in wild-type KRAS requires upstream stimulation by factors like FGF and EGF. ELISA assays showed that prostate cancer cells secrete low levels of FGF8b and EGF. Since DNPC often exhibits local progression, such as prostate enlargement and perineural invasion, we hypothesized that factors in the prostate microenvironment contribute to DNPC progression. To investigate, we focused on stromal cells in the prostate microenvironment, using PrSC, a prostate stromal cell line, and prostate cancer-associated stromal cell lines from prostate cancer patients. Compared to PrSC, PCaSCv2-9 secreted more FGF8b, while neither FGF8b nor EGF was secreted by the prostate cancer cells.

We previously reported that the secretion of chemokines like CCL2 and CCL20 significantly increases in prostate cancer cells after they become AR-independent or taxane-resistant [24–26, 48, 49]. Importantly, in the present study, CCL2 expression was increased in AR-independent LNCaP-SF cells compared with parental LNCaP cells. Moreover, AR knockdown in LNCaP cells resulted in a marked upregulation of CCL2 expression, indicating that suppression of AR signaling directly promotes CCL2 production in prostate cancer cells. We previously demonstrated that CCL2 secretion is markedly upregulated when prostate cancer cells are co-cultured with monocyte/macrophage cell lines such as THP-1 or U937 [24, 50, 51]. While earlier studies have shown that CCL2 acts directly on prostate cancer cells, our findings newly reveal that CCL2 also exerts functional effects on prostate cancer-associated stromal cells.

To our knowledge, no studies to date have directly linked CCL2 and FGF8b in stromal cells. However, it is conceivable that a literature-based analysis could suggest an indirect connection between CCL2 and FGF18. Wnt/β-catenin signaling has been reported to enhance FGF18 expression in several cancer types [52–56]. Conversely, CCL2 has been shown to activate Wnt/β-catenin signaling. For example, exogenous CCL2 activated hepatic stellate cells within the hepatic premetastatic niche via the Wnt/β-catenin pathway [57]. and promoted the expression of Wnt/β-catenin pathway-related proteins in HUVECs [58]. Consistent with these observations, our data indicate that in prostate cancer–associated stromal cells, CCL2 binds to CCR2 and promotes FGF8b secretion via activation of β-catenin signaling, thereby establishing a functional link between CCL2–CCR2 signaling and stromal FGF8b production. Although our data strongly suggest that CCL2 acts on CCR2-high stromal cells to enhance FGF8b secretion, this conclusion is currently inferred from CCR2 expression and ligand-induced responses, as functional blockade of CCR2 (e.g., genetic knockout or neutralizing antibodies) was not performed and should be addressed in future studies.

**Fig. 8:**
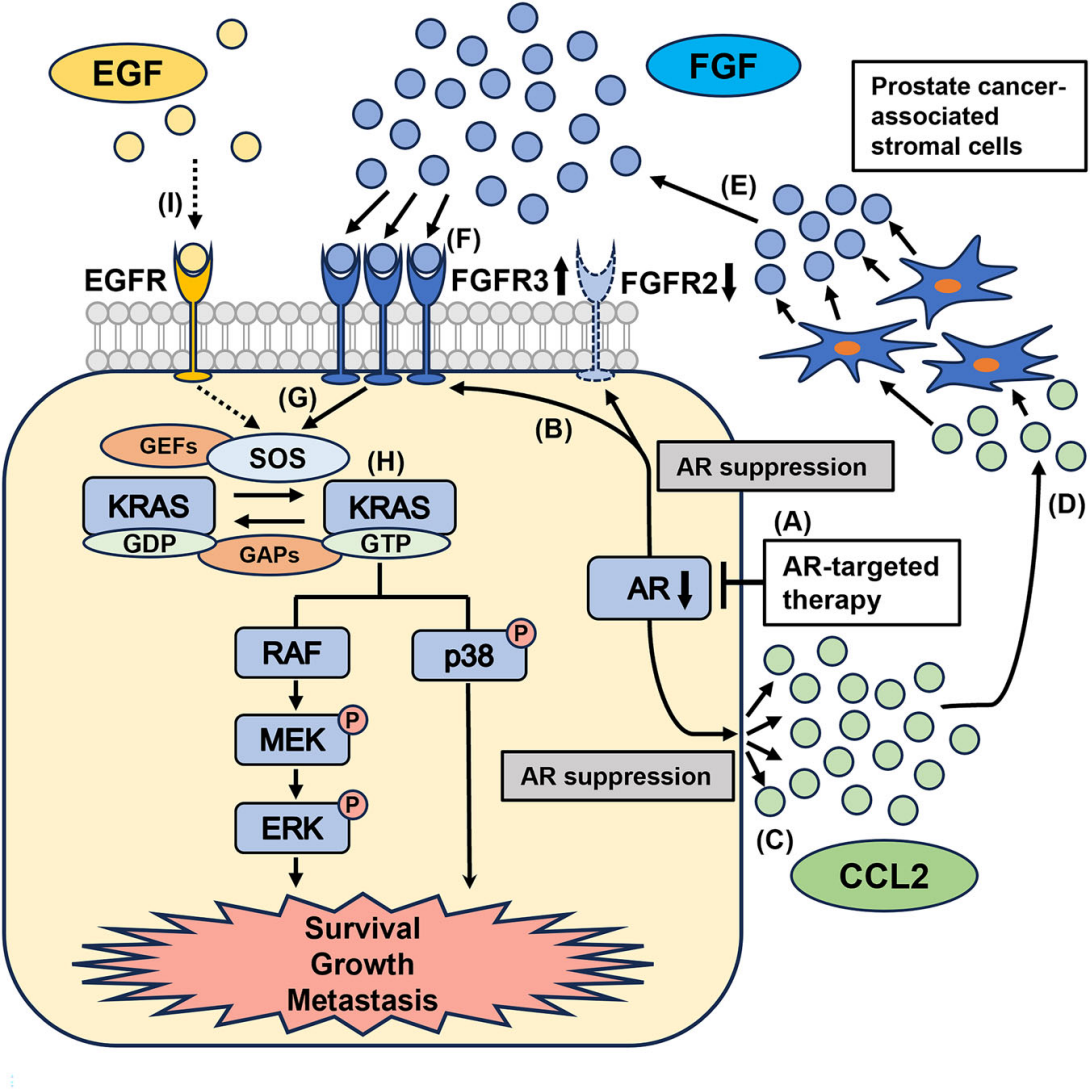
Schematic representation of enhanced KRAS signaling through the interaction between AR-independent prostate cancer cells and AR-independent prostate cancer-associated stromal cells. **A** AR signaling is suppressed by androgen deprivation therapy. **B** AR suppression leads to increased FGFR expression in prostate cancer cells. **C** AR suppression also raises CCL2 secretion from prostate cancer cells. **D** CCL2 influences AR-independent prostate cancer-associated stromal cells. **E** CCL2 stimulates FGF secretion from AR-independent prostate cancer-associated cells. **F** The released FGF binds to the upregulated FGFR in prostate cancer cells. **G** FGFR signaling activates KRAS through SOS. **H** Activated KRAS enhances downstream signaling and promotes prostate cancer progression. **I** EGF is scarce in the prostate cancer microenvironment, and KRAS is less activated by EGFR signaling compared to FGFR. GEFs guanine nucleotide exchange factors, GAPs GTPase-activating proteins.

Additionally, CCR2 expression was strong in prostate cancer-associated stromal cells from CRPC patients. These results suggest a malignant cascade driving DNPC: First, ARSI reduces AR dependency in prostate cancer cells and triggers CCL2 secretion. Second, CCL2 stimulates prostate cancer-associated stromal cells with upregulated CCR2 in a CRPC microenvironment, leading to FGF8b secretion. FGF8b binds to upregulated FGFR3 in CRPC cells, leading to the activation of wild-type-KRAS signaling and its downstream pathways, which results in increased migration, invasion, and proliferation (Fig. 8).

KRAS signaling is activated by FGF8b in AR knockdown LNCaP cells, AR-independent LNCaP-SF, and DU145, which has a KRAS mutation. Furthermore, CCL2 in the prostate microenvironment further enhances FGF8b-mediated KRAS signaling. CRPC, which shows DNPC characteristics, often relapses in the prostate [10]. which aligns with the CCL2- and FGF8b-mediated activation of KRAS signaling in the prostate microenvironment, contributing to DNPC cells metastasizing to other organs. In clinical practice, even though complete DNPC may not emerge during ARSI treatment, a low PSA with negative NED markers could indicate a KRAS-activated condition, referred to as “semi-DNPC.”

Our data suggest several potential treatment targets, including CCL2–CCR2 axsis, FGF–FGFR signaling, and KRAS itself. KRAS signaling can be suppressed by pan-KRAS inhibitors, which target both KRAS mutations and activated wild-type KRAS, potentially playing a key role in DNPC development [59–62]. Although not validated in this study, combining a pan-KRAS inhibitor with a MEK inhibitor [21]. a FGFR inhibitor [27]. a CCL2 inhibitor [63]. or a CCR2 inhibitor [64]. may enhance the anti-tumor effects on AR-independent prostate cancer. The combination of ENZ and FGFR inhibitors has been shown to be effective in prostate cancer [65]. and combining pan-KRAS inhibitors with ARSIs is also expected to be beneficial. Interestingly, AKT signaling remained largely unaffected in both DU145 and LNCaP-SF cells, indicating that AKT is not a key driver in DNPC, despite its reported role in cancer progression in AR-suppressed CRPC [66]. While AKT inhibitors have been shown to be effective in PTEN-loss CRPC and their clinical use is expected to grow [67]. They may be less effective in DNPC. The current study does not include transcriptomic or GSEA-based validation against DNPC datasets, which would be essential to strengthen the translational relevance of our findings. Moreover, while we employed FGFR3-specific inhibition and FGF8b neutralization to probe the stromal FGF8b–KRAS pathway, additional mechanistic interventions—such as CCR2 blockade, FGF8b knockdown, and KRAS/MEK rescue experiments—are needed to fully establish causality and cell-type specificity. Future studies incorporating patient-derived DNPC transcriptomes and more refined stromal models will be critical to validate and extend the proposed pathway. While our in vivo experiments using the DU145 xenograft model provided valuable insights into KRAS-related signaling, we acknowledge the limitation of relying on a single cell line with a modest sample size. Expanding in vivo validation to additional models would strengthen the generalizability of our findings. However, due to time constraints and ethical considerations regarding animal use, further experiments were not feasible within the scope of this study. To complement the existing data, we performed additional analyses of apoptotic signaling in xenograft tissues, which support the mechanistic relevance of our observations.

In conclusion, our study is the first to suggest that CCL2–FGF–KRAS signaling could be a potential therapeutic target for treating AR-independent prostate cancer in late-stage DNPC.

## Supplementary information

Supplemental Material

Full blot of WB

## Data Availability

Gene expression profiling analysis was performed using publicly available datasets from the NCBI Gene Expression Omnibus (GEO; https://www.ncbi.nlm.nih.gov/geo/) database in human prostate cancer tissues. The datasets GDS1390 (Clin Cancer Res 2005 Oct 1;11(19 Pt 1):6823-34. PMID: 16203770) and GDS1439 (Cancer Cell 2005 Nov;8 [5]. 393-406. PMID: 16286247) were used. Raw data generated in this study were obtained at the Department of Integrative Cancer Therapy and Urology, Kanazawa University Graduate School of Medical Science. The derived datasets supporting the findings of this study are available from the corresponding author upon reasonable request.
