# Supplementary material for: Fibroblast-mediated KRAS activation in double-negative prostate cancer
Source: Cell Death Dis. 2026 May 2;17(1):403. doi: 10.1038/s41419-026-08800-3 (PMC13139504; doi:10.1038/s41419-026-08800-3)
Supplement: Supplementary file 1 — Supplemental Material [file 41419_2026_8800_MOESM1_ESM.docx]

**SUPPLEMENTAL INFORMATION**


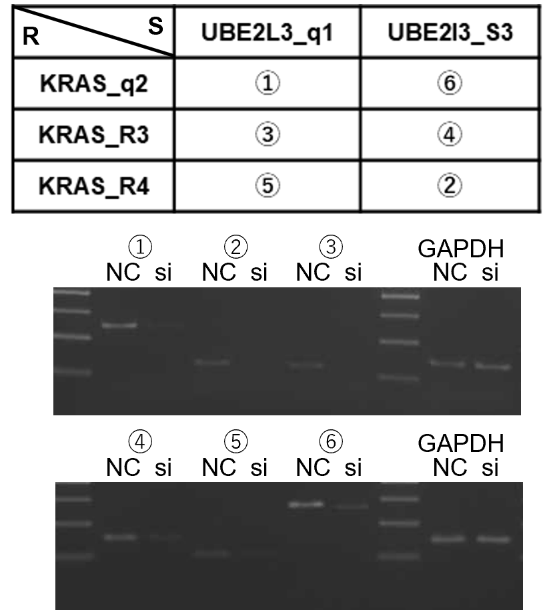


**Figure S1. Selection of primers for detecting UBE2L3-KRAS.**

We identified the most effective primers for detecting UBE2L3-KRAS. Six primers were designed based on the UBE2L3-KRAS sequence combinations from a reference study (*20*). The primer combination of KRAS_q2 and UBE2L3_q1 was used for this analysis.


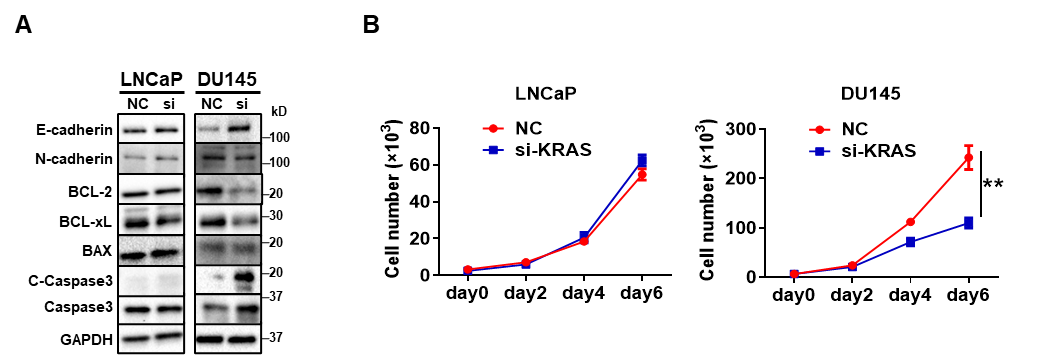


**Figure S2. KRAS knockdown promotes apoptosis and suppresses proliferation in AR-independent CRPC.**

**(A)** WB analysis of EMT and apoptosis signaling in prostate cancer cells following KRAS knockdown using si-KRAS#1. **(B)** Proliferation assay of LNCaP and DU145 cells after KRAS knockdown using si-KRAS#1 (n = 3/group). ***p* < 0.01


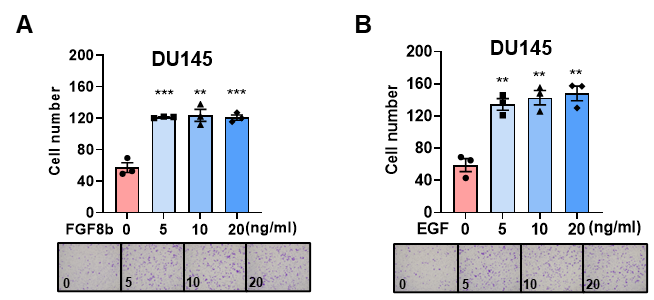


**Figure S3. FGF8b and EGF promote cancer progression in AR-independent CRPC by activating KRAS signaling.**

**(A)** Invasion assay of DU145 cells treated with various concentrations of FGF8b (n = 3/group). **(B)** Invasion assay of DU145 cells treated with various concentrations of EGF (n = 3/group). ***p* < 0.01; ****p* < 0.001


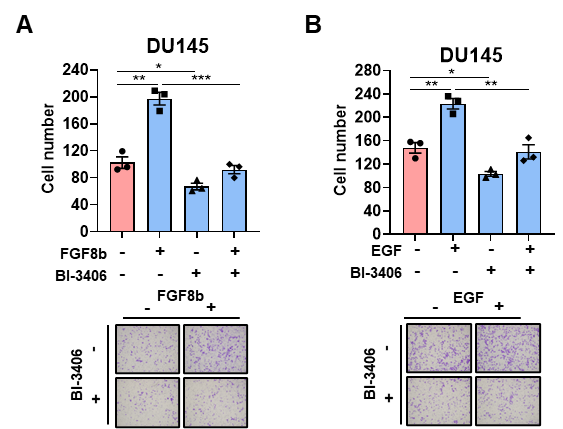


**Figure S4. BI-3406 inhibits CRPC progression induced by FGFR and EGFR activation**

**(A)** Invasion assay of DU145 cells treated with FGF8b (10 ng/mL) and BI-3406 (0.1 μM) (n = 3/group). **(B)** Invasion assay of DU145 cells treated with EGF (10 ng/mL) and BI-3406 (0.1 μM) (n = 3/group). **p* < 0.05; ***p* < 0.01; ****p* < 0.001


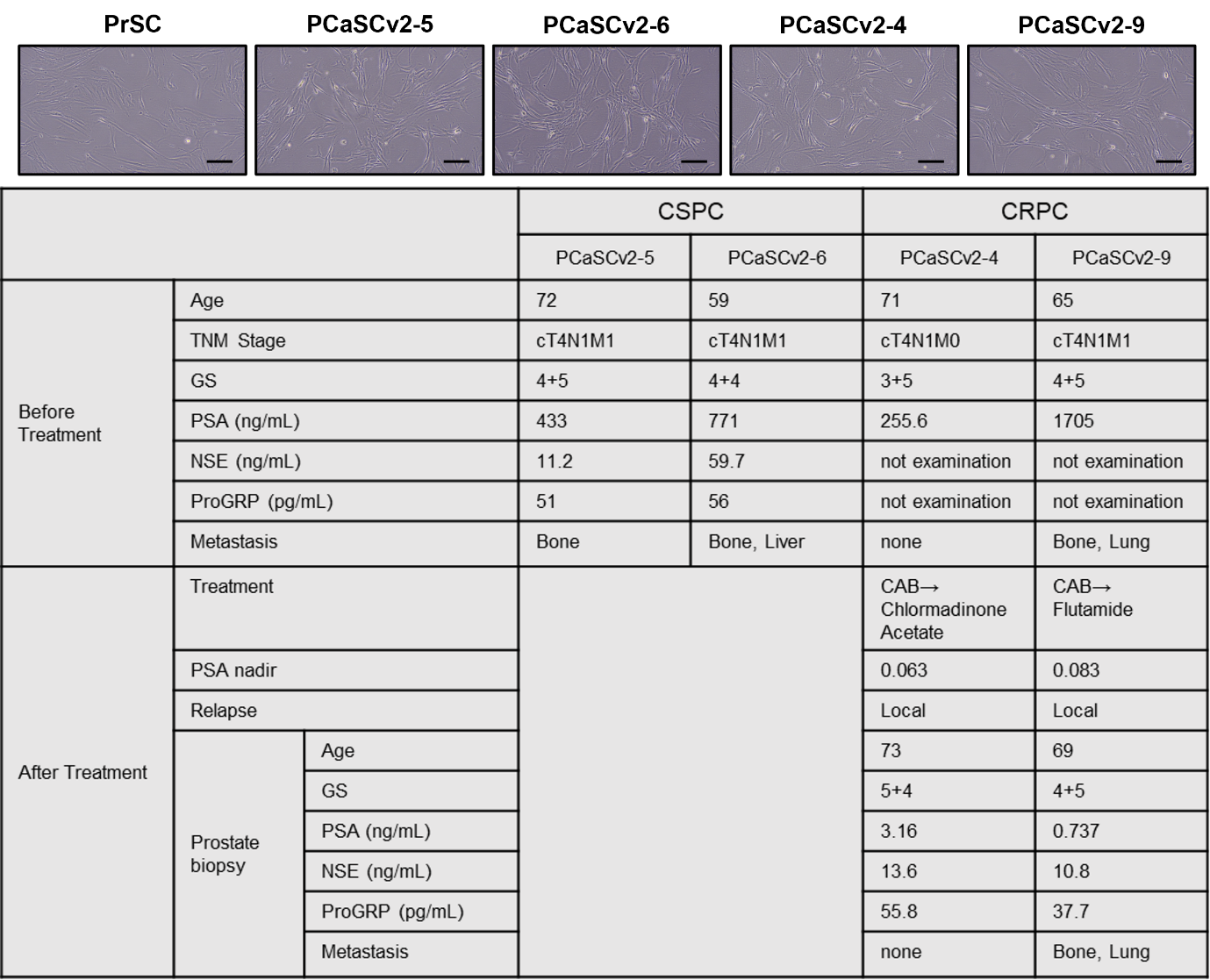
**Figure S5. Characteristics of prostate cancer-associated stromal cells derived from prostate cancer patients**

All prostate cancer-associated stromal cells exhibited a morphology similar to PrSC cells. PCaSCv2-5 and PCaSCv2-6 were derived from CSPC patients with metastases, who had both elevated PSA and negative NED markers. PCaSCv2-4 was established from a patient who relapsed with elevated PSA following combined androgen blockade treatment. PCaSCv2-9 was established from a patient with local prostate recurrence after combined androgen blockade treatment, who had low PSA and negative NED markers at the time of PCaSCv2-9 establishment. Scale bars = 200 µm.


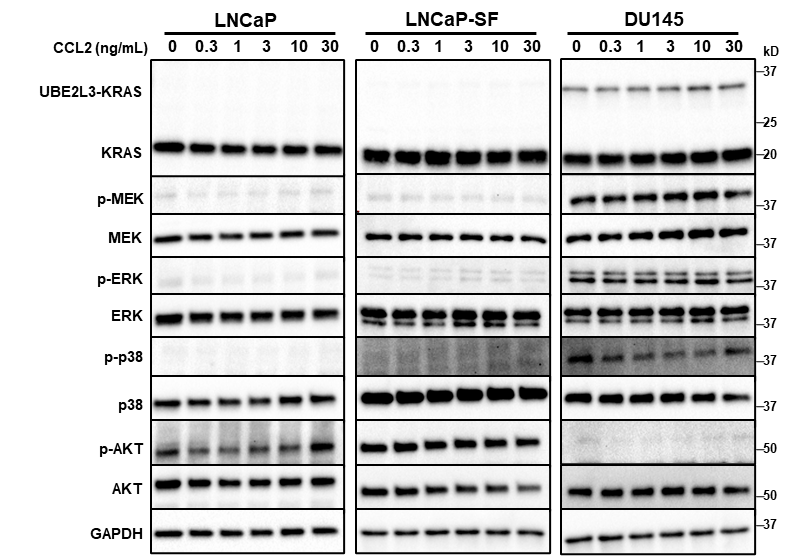


**Figure S6. Stimulation of prostate cancer cells with CCL2 did not alter KRAS downstream signaling.**

WB analysis of KRAS downstream signaling in LNCaP, LNCaP-SF, and DU145 cells treated with various concentrations of CCL2.

**
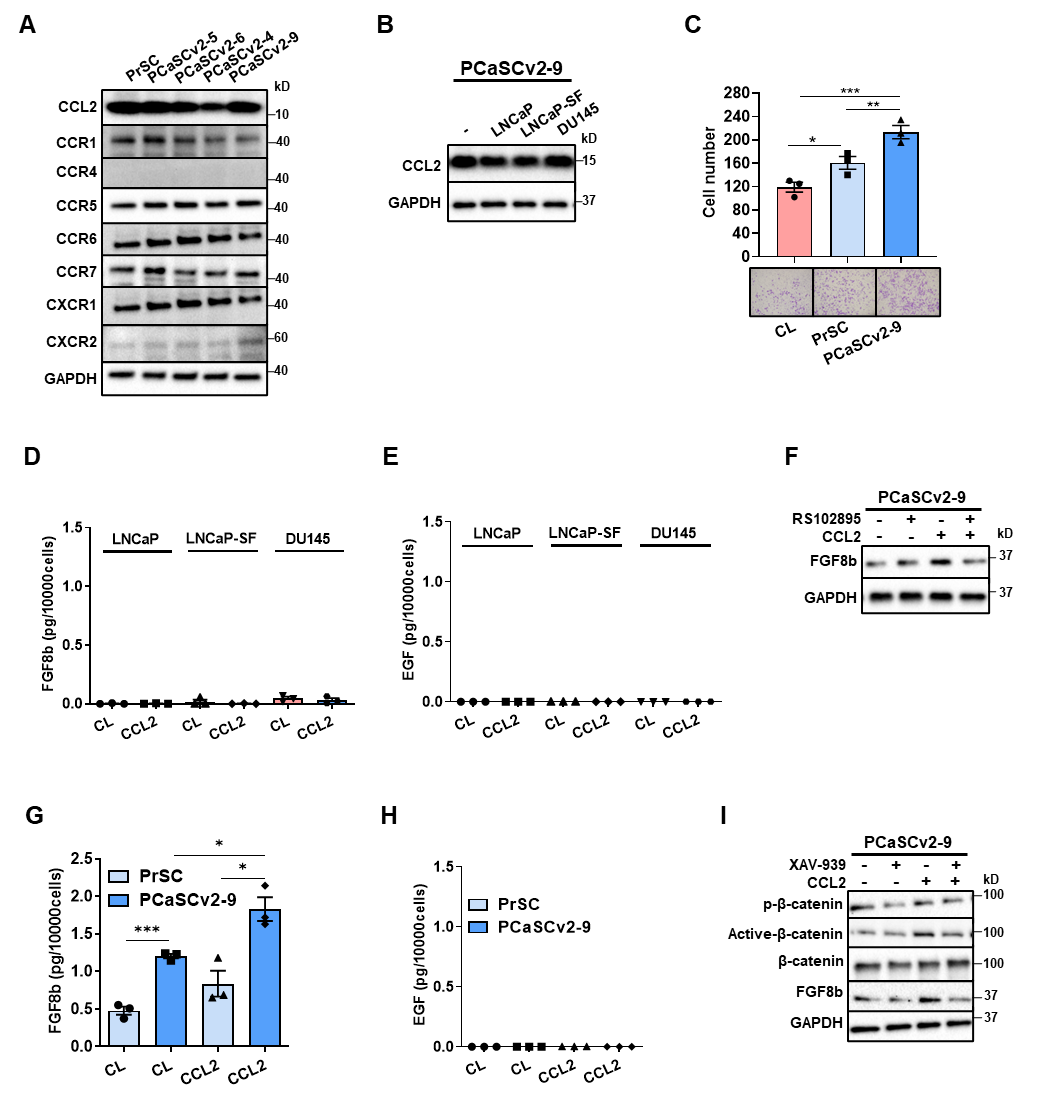
**

**Figure S7. FGF8b and EGF secretions from prostate cancer cells and cancer-associated stromal cells.**

**(A)** WB analysis of chemokine receptors in prostate cancer–associated stromal cells.

**(B)** WB analysis of CCL2 expression in PCaSCv2-9 cells co-cultured with prostate cancer cells. PCaSCv2-9 cells were co-cultured with prostate cancer cells for 24 h.

**(C)** Invasion assay of DU145 cells co-cultured with PrSC or PCaSCv2-9 cells (n = 3/group). **(D and E)** Measurement of FGF8b (D) and EGF (E) secretion from LNCaP, LNCaP-SF, and DU145 cells treated with CCL2 (10 ng/mL) from three different experiments. Cells were cultured for 96 h, and the media were collected and analyzed by ELISA. **(F)** WB analysis of FGF8b in PCaSCv2-9 cells treated with CCR2 antagonist (RS102895, 10 μM) and CCL2 (10 ng/mL) for 24 h. **(G)** Measurement of FGF8b secretion from PrSC and PCaSCv2-9 cells from three different experiments. Cells were cultured in media with 5% CCS and CCL2 (10 ng/mL) for 96 h, and the media were collected and analyzed by ELISA. **(H)** Measurement of EGF secretion from PrSC and PCaSCv2-9 cells from three different experiments. Cells were cultured in media with 5% CCS and CCL2 (10 ng/mL) for 96 h, and the media were collected and analyzed by ELISA. **(I)** WB analysis of β-catenin signaling in PCaSCv2-9 cells treated with XAV-939 (10 μM) and CCL2 (10 ng/mL) for 24 h. **p* < 0.05; ***p* < 0.01; ****p* < 0.001


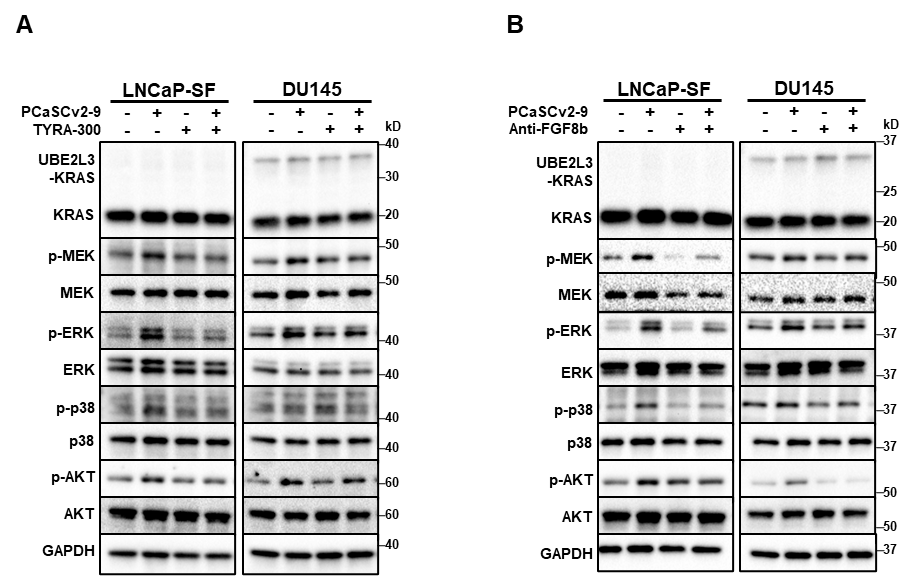


**Figure S8. The FGFR3 inhibitor (TYRA-300) and FGF8b-neutralizing antibody inhibit CRPC progression induced by co-culture with PCaSCv2-9 cells.**

**(A) (B)** WB analysis of KRAS downstream signaling in LNCaP-SF and DU145 cells co-cultured with PCaSCv2-9 cells and treated with TYRA-300 (50 nM) (A) or FGF8b neutralizing antibody (1 ng/mL) (B).

**
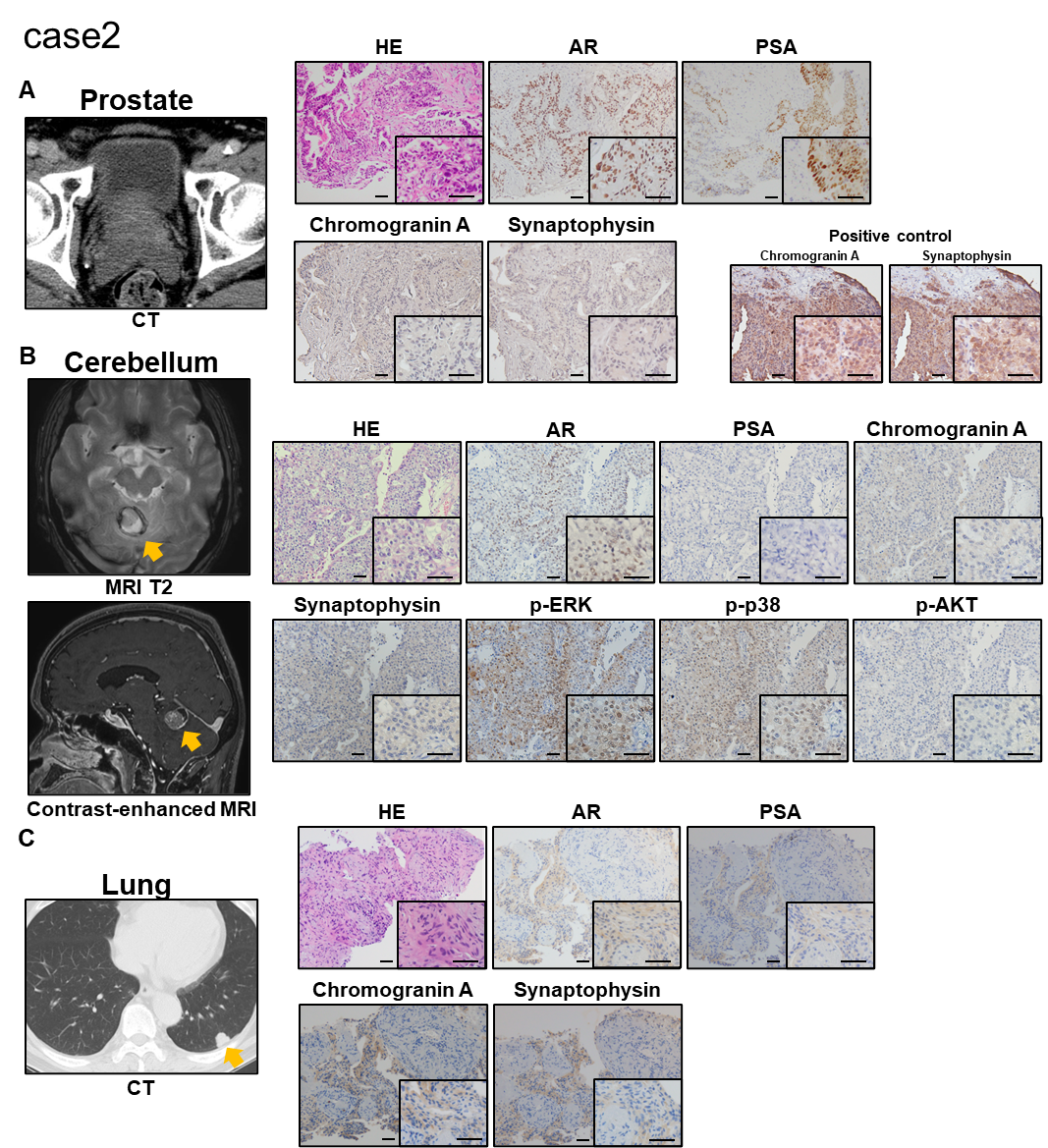
**

**Figure S9. Clinical findings of DNPC Case 2 with KRAS alteration.**

IHC analysis of Case 2 shows that pretreatment prostate cancer tissue is positive for AR and PSA, while cerebellar and lung metastases are negative for PSA, chromogranin A, and synaptophysin, indicating DNPC features. **(A)** CT scan and IHC staining of the prostate before treatment showing positive PSA and negative chromogranin A and synaptophysin. **(B)** Magnetic resonance imaging and IHC staining of cerebellar metastasis during combined androgen blockade treatment, with negative PSA, chromogranin A, and synaptophysin but positive p-ERK and p-p38, downstream markers of KRAS activation. **(C)** CT scan and IHC staining of lung metastasis during abiraterone treatment, showing negative PSA, chromogranin A, and synaptophysin. Scale bars = 50 µm.


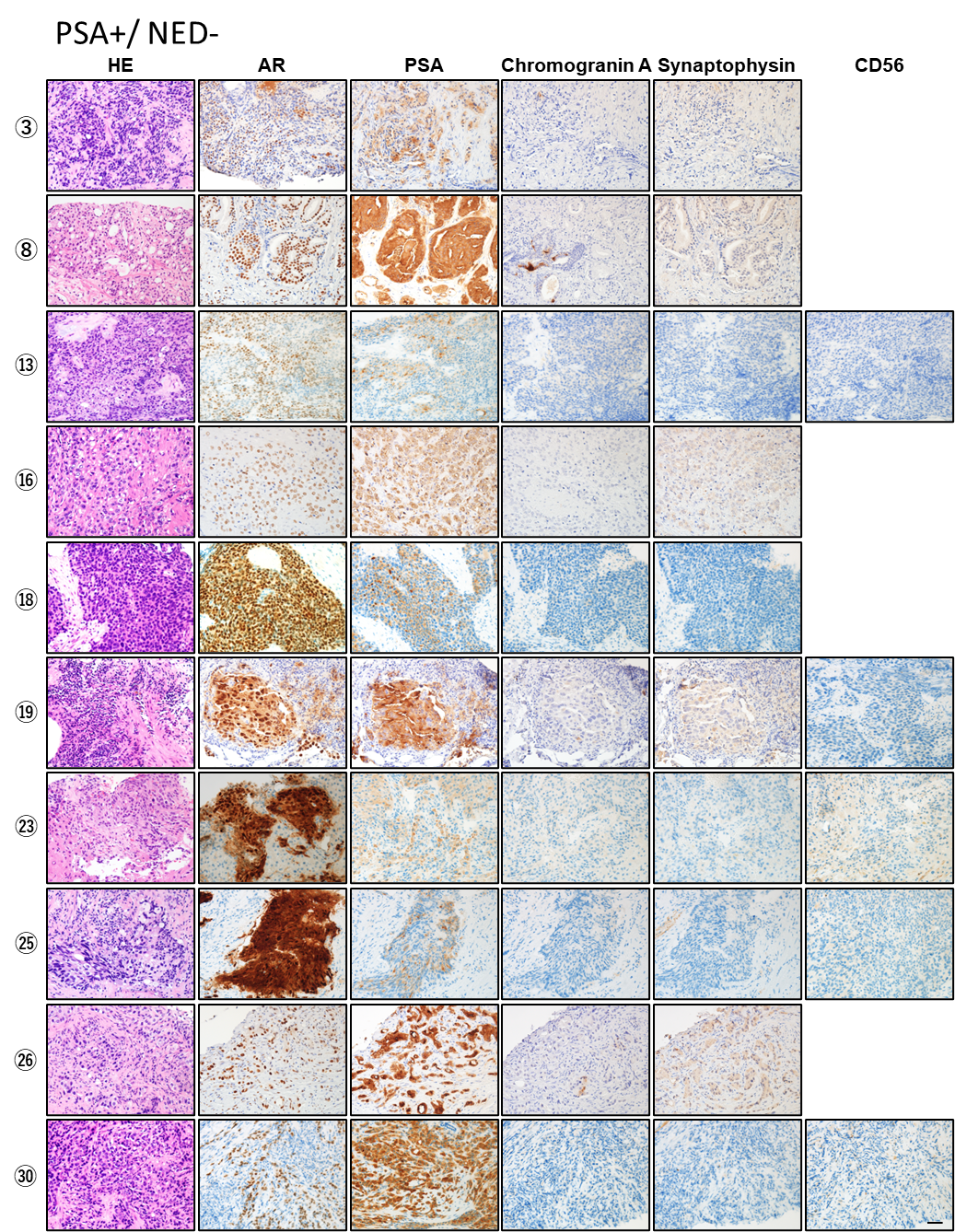


**Figure S10. IHC (PSA⁺/NED⁻) of patients who underwent cancer genome profiling.**

Scale bar = 50 µm.


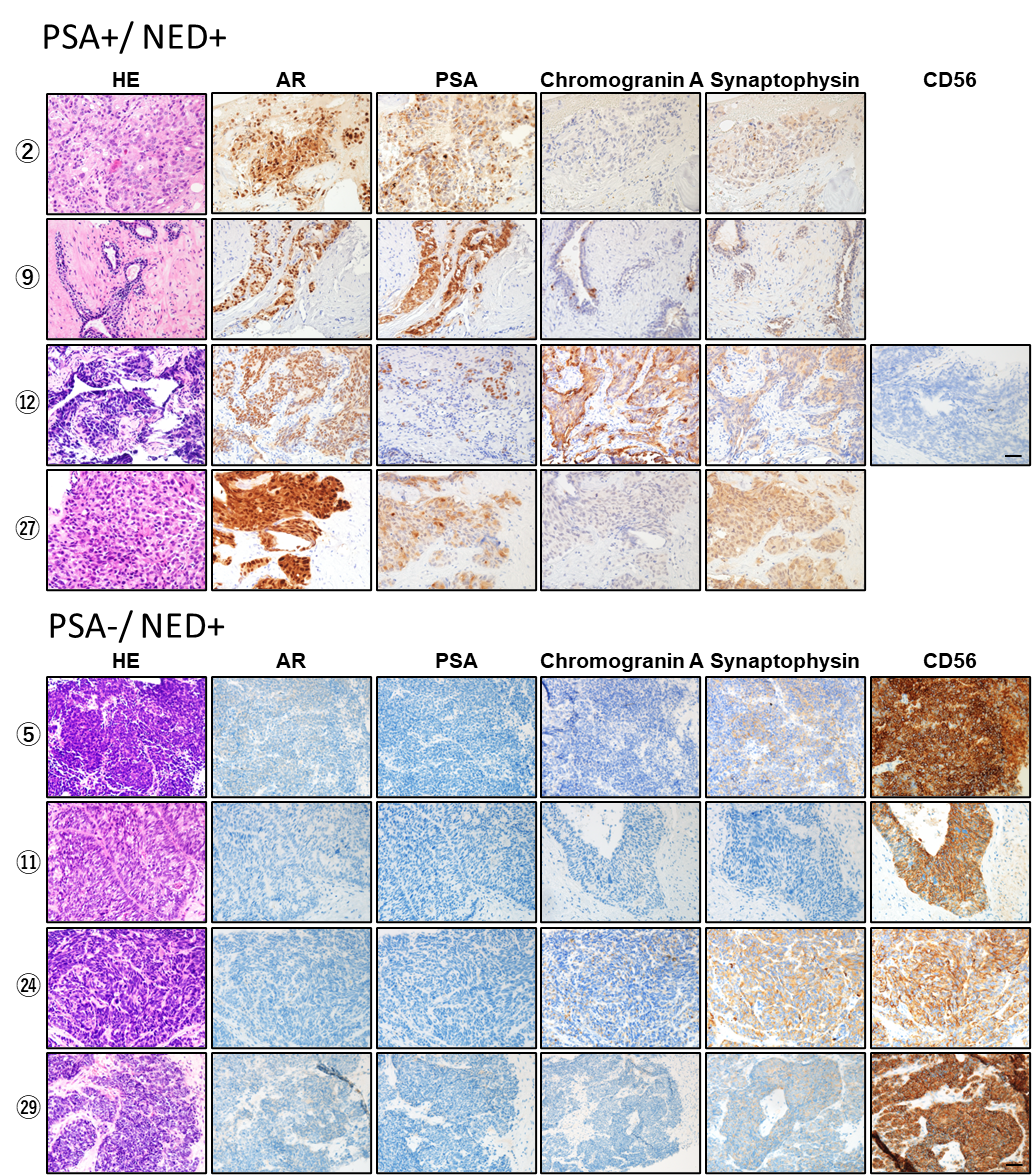


**Figure S11. IHC (PSA⁺ or PSA⁻/NED⁻) of patients who underwent cancer genome profiling.**

Scale bars = 50 µm.

| **Table S1. Summary of antibodies, proteins, and chemicals used** | | | | |
| --- | --- | --- | --- | --- |
| REAGENT or RESOURCE | | SOURCE | | IDENTIFIER |
| Antibodies | |  | |  |
| Rabbit Polyclonal Anti-KRAS | | Proteintech | | 12063-1-AP; RRID: AB_878040 |
| Rabbit Polyclonal Anti-p-AKT | | Cell Signaling | | 9271; RRID: AB_329825 |
| Rabbit Polyclonal Anti-AKT | | Cell Signaling | | 9272; RRID: AB_329827 |
| Rabbit Polyclonal Anti-p-p44/42 MAPK (p-Erk1/2) | | Cell Signaling | | 9101; RRID: AB_331646 |
| Rabbit Polyclonal Anti-p44/42 MAPK (Erk1/2) | | Cell Signaling | | 9102; RRID: AB_330744 |
| Rabbit Monoclonal Anti-p-MEK | | Cell Signaling | | 9154; RRID: AB_2138017 |
| Mouse Monoclonal Anti-MEK | | Cell Signaling | | 4694; RRID: AB_10695868 |
| Rabbit Monoclonal Anti-p-p38 MAPK | | Cell Signaling | | 4511; RRID: AB_2139682 |
| Rabbit Monoclonal Anti-p38 MAPK | | Cell Signaling | | 8690; RRID: AB_10999090 |
| Rabbit Monoclonal Anti-AR | | abcam | | ab133273; RRID: AB_11156085 |
| Rabbit Monoclonal l Anti-PSA/KLK3 | | Cell Signaling | | 2475; RRID: AB_2797601 |
| Rabbit Polyclonal Anti-p-FGFR | | Cell Signaling | | 3471; RRID: AB_331072 |
| Rabbit Monoclonal Anti-FGFR1 | | Cell Signaling | | 9740; RRID: AB_11178519 |
| Rabbit Monoclonal Anti-FGFR2 | | Cell Signaling | | 23328; RRID: AB_2798862 |
| Rabbit Monoclonal Anti-FGFR3 | | Cell Signaling | | 4574; RRID: AB_2246903 |
| Rabbit Monoclonal Anti-FGFR4 | | Cell Signaling | | 8562; RRID: AB_10891199 |
| Rabbit Monoclonal Anti-p-EGFR | | Cell Signaling | | 3777; RRID: AB_2096270 |
| Rabbit Monoclonal Anti-EGFR | | Cell Signaling | | 4267; RRID: AB_2246311 |
| Mouse Monoclonal Anti-FGF8 | | R&D Systems | | MAB323; RRID: AB_2102956 |
| Rabbit Polyclonal Anti-EGF | | GeneTex | | GTX111176; RRID: AB_10723108 |
| Rabbit Polyclonal Anti-CCR1 | | abcam | | ab86668; PRID: AB_10860331 |
| Rabbit Monoclonal Anti-CCR2 | | Cell Signaling | | 12199; RRID: AB_2797844 |
| Rabbit Monoclonal Anti-CCR4 | | Cell Signaling | | 59945; PRID: N/A |
| Mouse Monoclonal Anti-CCR5 | | abcam | | ab110103; PRID: AB_10865804 |
| Rabbit Polyclonal Anti-CCR6 | | abcam | | ab227036; PRID: AB_2892731 |
| Rabbit Polyclonal Anti-CCR7 | | abcam | | ab103404; PRID: AB_10712246 |
| Rabbit Polyclonal Anti-CXCR1 | | abcam | | ab124344; PRID: AB_10974083 |
| Rabbit Polyclonal Anti-CXCR2 | | abcam | | ab14935; PRID: AB_301526 |
| Rabbit Monoclonal Anti-E-Cadherin | | Cell Signaling | | 3195; PRID: AB_2291471 |
| Rabbit Monoclonal Anti-N-Cadherin | | abcam | | ab76011; PRID: AB_1310479 |
| Rabbit Polyclonal Anti-Cleaved Caspase 3 | | Cell Signaling | | 9661; PRID: AB_2341188 |
| Rabbit Monoclonal Anti-Caspase 3 | | abcam | | ab32351; PRID: AB_725946 |
| Mouse Monoclonal Anti-BCL-2 | | Cell Signaling | | 15071; PR137369 |
| Rabbit Monoclonal Anti-BCL-xL | | Cell Signaling | | 2764; PRID: AB_2228008 |
| Rabbit Polyclonal Anti-BAX | | Cell Signaling | | 2772; PRID: AB_10695870 |
| Rabbit Monoclonal Anti-MCP1(CCL2) | | abcam | | ab214819; PRID: AB_3083804 |
| Rabbit Polyclonal Anti-Synaptophysin | | Proteintech | | 17785-1-AP; RRID: AB_2271365 |
| Rabbit Polyclonal Anti-Chromogranin A | | Proteintech | | 10529-1-AP; RRID: AB_2081122 |
| Mouse Monoclonal Anti-Ki-67 | | Agilent Technologies | | M7248; RRID: AB_2142378 |
| Rabbit Monoclonal Anti-CD56 | | Nichirei Biosciences | | 418191; RRID: N/A |
| Rabbit Polyclonal Anti-p-β-catenin (Thr41/Ser45) | | Cell Signaling | | 9565; RRID: AB_331731 |
| Rabbit Monoclonal Anti-β-catenin | | Cell Signaling | | 8480; RRID: AB_11127855 |
| Rabbit Monoclonal Anti-Active-β-catenin (Ser33/37/Thr41) | | Cell Signaling | | 8814; RRID: AB_11127203 |
| Rabbit Polyclonal Anti-p-GSK3β (Ser9) | | Cell Signaling | | 9322; RRID: AB_2115196 |
| Rabbit Monoclonal Anti-GSK3β | | Cell Signaling | | 12456; RRID: AB_2636978 |
| Mouse Monoclonal Anti-GAPDH | | Proteintech | | 60004-1-Ig; RRID: AB_2107436 |
| Goat Monoclonal Anti-rabbit IgG-HRP-linked | | Cell Signaling | | 7074; RRID: AB_2099233 |
| Goat Monoclonal Anti-mouse IgG-HRP Conjugate | | nacalai tesque | | 01803-4401803-44; RRID: N/A |
| Chemicals and recombinant proteins | |  | |  |
| Recombinant FGF8b | | R&D Systems | | 423-F8-025 |
| Recombinant Human EGF | | PeproTech | | AF-100-15 |
| Recombinant Human CCL2 | | BioLegend | | 571402 |
| BI-3406 | | MedChemExpress | | HY-125817 |
| Futibatinib | | MedChemExpress | | HY-100818 |
| XAV-939 | | Selleck Chemicals | | S1180 |
| TYRA-300 | | MedChemExpress | | HY-159642 |
| RS102895 | | abcam | | Ab 120812 |
|  |  | |  | |

| **Table S2. Primer list** | | | | | | | |
| --- | --- | --- | --- | --- | --- | --- | --- |
| Gene | Refseq. | Primer | Type | Bases | Exon | Sequence (5' to 3') | application |
| KRAS | NM_001369786.1 | KRAS_F | Sense | 398 | Exon 3 | CTGGGGAGGGCTTTCTTTGT | qPCR |
| KRAS | NM_001369786.1 | KRAS_R | Reverse | 579 | Exon 4 | TGCTAAGTCCTGAGCCTGTT | qPCR |
| KRAS | NM_004985 | KRAS_q2 | Reverse | 349 | Exon 3 | CTCCTCTTGACCTGCTGTGTCG | RT-PCR, qPCR |
| KRAS | NM_004985 | KRAS_R3 | Reverse | 228 | Exon 2 | AGCTGTATCGTCAAGGCACTCT | RT-PCR |
| KRAS | NM_004985 | KRAS_R4 | Reverse | 204 | Exon 2 | CCTACGCCACCAGCTCCAACTA | RT-PCR |
| UBE2L3 | NM_003347 | UBE2L3_q1 | Sense | 345 | Exon 3 | ATTAGTGCCGAAAACTGGAAGC | RT-PCR, qPCR |
| UBE2L3 | NM_003347 | UBE2L3_S3 | Sense | 316 | Exon 3 | ACGAAAAGGGGCAGGTCTGTCT | RT-PCR |
| GAPDH | NM_002046.7 | GAPDH_F | Sense | 254 | Exon 4 | GTCAAGGCTGAGAACGGGAA | RT-PCR, qPCR |
| GAPDH | NM_002046.7 | GAPDH_R | Reverse | 380 | Exon 5 | GCCTTCTCCATGGTGGTGAA | RT-PCR, qPCR |

| **Table S3.**  **Clinicopathological characteristics of the patients** | | | | | | | | | | |
| --- | --- | --- | --- | --- | --- | --- | --- | --- | --- | --- |
| No. | Age*/† | CGP Test | Specimen for CGP | Sample for IHC | TNM classification* | GS* | PSA*,  ng/mL | PSA†,  ng/mL | NSE†, ng/mL | ProGRP†, pg/mL |
| 1 | 68/70 | F1 | Bladder | Bladder | cT4N1M1b | 5+4 | 3.079 | 0.007 | 14.1 | 65 |
| 2 | 68/75 | F1 | Bone marrow | Bone marrow | cT2aN0M0 | 4+4 | 6.733 | 92 | 6.8 | 76 |
| 3 | 75/79 | F1 | Prostate | Prostate | cT4N1M1c | 5+5 | 93.9 | 386 | 27.4 | 57 |
| 4 | 67/71 | F1 | Cerebellum | Cerebellum | cT2cN0M0 | 4+5 | 2.33 | 0.0005 | 8.3 | 57 |
| 5 | 71/73 | F1 | Liver | Liver | cT4N1M1b | 4+5 | 110.46 | 0.376 | 36.2 | 71 |
| 6 | 72/74 | NCCOP | Prostate |  | cT4N1M1b | 4+5 | 2947 | 86.9 | 12.7 | 100 |
| 7 | 66/70 | F1 liquid | Blood |  | cT2aN0M0 | No bipsy | 6.07 | 171 | 20 | 93 |
| 8 | 59/74 | F1 liquid | Blood | Prostate | cT3bN1M0 | 4+3 | 98.1 | 6.7 | 9 | 48 |
| 9 | 69/71 | F1 | Prostate | Prostate | cT2bN0M1b | No bipsy | 808 | 96.8 | 6.3 | 134 |
| 10 | 66/75 | F1 liquid | Blood |  | cT3aN1M1b | 4+5 | 417 | 46.8 | 10.2 | 49.6 |
| 11 | 72/73 | F1 | Prostate | Prostate | cT4N0M1b | 4+4 | 53.2 | 0.022 | 19.6 | 89.4 |
| 12 | 61/70 | F1 liquid | Blood | Prostate | cT3bN1M1c | 5+5 | 41.844 | 0.546 | 14.2 | 41.5 |
| 13 | 66/67 | F1 | Bone | Bone | cT4N1M0 | 5+5 | 10 | 2.53 | 11 | 58.1 |
| 14 | 59/64 | F1 | Prostate |  | cT1cN0M1b | 4+4 | 9.91 | 37.4 | 13 | 40.3 |
| 15 | 70/77 | NCCOP | Prostate |  | cT2bN1M1b | 5+4 | 2920 | 0.494 | 16.4 | 45.4 |
| 16 | 67/69 | F1 | Prostate | Prostate | cT2cN0M0 | 5+4 | 5.235 | 20.6 | 14 | 31.9 |
| 17 | 72/77 | F1 | Prostate |  | cT4N1M1b | 4+5 | 115 | 173 | 6.5 | 65.5 |
| 18 | 64/65 | F1 | Prostate | Prostate | cT3aN1M1b | 5+4 | 2522 | 410 | 30.4 | 89.7 |
| 19 | 66/72 | F1 liquid | Blood | Lymph node | cT2bN1M0 | 4+4 | 10.08 | 69.2 | 10.4 | 50.9 |
| 20 | 66/73 | F1 | Prostate |  | cT4N1M1b | 4+5 | 1188 | 32.6 | 10.3 | 76.3 |
| 21 | 75/76 | F1 | Prostate |  | cT3bN1M1b | 5+4 | 4947 | 389 | 6.6 | 117 |
| 22 | 54/62 | F1 liquid | Blood |  | cT2bN0M1b | 4+4 | 3.5 | 0.373 | 9.5 | 47.5 |
| 23 | 71/75 | F1 | Liver | Liver | cT2aN0M0 | 4+5 | 6.2 | 206 | 13.4 | 55.7 |
| 24 | 75/79 | NCCOP | Prostate | Prostate | cT3aN0M0 | 4+5 | 12.885 | 0.0005 | 19.1 | 4885 |
| 25 | 63/67 | F1 | Prostate | Prostate | cT3bN1M1b | 4+5 | 245 | 10.7 | 7.1 | 56.4 |
| 26 | 69/76 | F1 liquid | Blood | Prostate | cT4N0M0 | 4+5 | 42.67 | 23.1 | 11.2 | 89.5 |
| 27 | 59/61 | NCCOP | Liver | Liver | cT3bN1M1c | 4+4 | 433 | 34.6 | 32.6 | 58.2 |
| 28 | 59/61 | NCCOP | Lymph node |  | cT4N1M1a | 5+5 | 38.1 | 8.06 | 6.7 | 31.7 |
| 29 | 73/74 | F1 | Bone | Bone | cT4N1M1b | 5+5 | 8.66 | 0.026 | 269 | 117 |
| 30 | 74/75 | F1 | Prostate | Prostate | cT3bN1M1b | 5+4 | 70.8 | 11.1 | 8.6 | 47.1 |
| *At first diagnosis, †at CGP, No.1 and No.4 indicates Case 1 and 2, respectively.  CGP, Cancer Genomic Profiling; IHC, Immunohistochemistry; TNM, Tumor-Node-Metastasis; GS, Gleason score; PSA, prostate-specific antigen; NSE, neuron-specific enolase; proGRP, pro-gastrin-releasing peptide; F1, FoundationOne®CDx Cancer Genomic Profile; NCCOP, OncoGuide NCC Oncopanel System | | | | | | | | | | |

| **Table S4. Comprehensive cancer genomic profiling results of the patients** | |
| --- | --- |
| No | Genetic abnormality |
| 1 | KRAS(amplification), ATM Y2470D(74%), APC R499*(40%), APC T1220fs*5(7%), APC Q1303*(9%),  APC N145fs*2(9%), TET2 R1452(32%), AR amplification(CN=37), NKX2-1 amplification(CN=18) |
| 2 | PTEN loss exson1-4, PIK3R1 R590W(2%), TP53 Y236*(18%), TP53 P60*(20%) |
| 3 | BRCA2 S1341fs*33(64%), CASP8 splice site 305+1_305+12delGTGGGTGGAAAC(8%) |
| 4 | KRAS Q61L(59%), PTEN loss, BARD1 R150*(96%), MYCN amplification(CN=6)-equivocal, TET2 detection exson11 |
| 5 | BRCA2 L1908fs*2(50%), TMB-high(10/Mb), PTEN D58fs*7(79%), MYC amplification(CN=8) |
| 6 | TP53 N263_L264insGN(26%), TP53 Y220C |
| 7 | FGF19 amplification(CN=6), FGF3 amplification(CN=6), FGF4 amplification(CN=6),TMB high(13/Mb),  CDK12 S343fs*8(26.5%),BARF, CCND amplification(CN=6), MUTYH splice site892-2A>G(48%) |
| 8 | None |
| 9 | TP53 H115fs*8(12%) |
| 10 | TP53 H115fs*28(0.13%), R156H(49.1%) |
| 11 | APC R1858*(67.66%), TP53 C277(71.34%), AMAD4 D493H(69.11%), RB1 D111fs*14(7.89%), FAS loss, PTEN loss |
| 12 | TP53 L111Q(0.23%), TP53 R110fs*14(1.97%), TMRPSS2-ERG fusion |
| 13 | BRCA2 T3033fs*11, ATM K2811fs*46, CHeK1 T226fs*14, TMB-high( 45/Mb), MSI-high, AR W742(84.5%),  ARID1A D1850fs*33, PTEN T319fs*1, PIK3CA G122D, R93Q, D350G, R173H, V290fs*1, FENCA E1255fs*12(41.4%), TET2 K1439fs*9(41.6%) |
| 14 | None |
| 15 | TMB high(403.9/Mb) |
| 16 | FGF14 A236V(62.4%), TMPRS2-ERG fusion, TP53 rearrangement exson7 |
| 17 | KMDA6A loss exons 5-29, TP53 G244D(33.7%) |
| 18 | CDK12 Y295fs*43(39.5%),CCND1 amplification-equivocal(CN=7), PIK3CB amplification,  BRAF amplification-equivocal(CN=8), MDM4 amplification(CN=8), PIK3C2B amplification(CN=8),  PRKCL amplification(CN=10), RETC amplification, TP53 R333fs*12(68.5%) |
| 19 | ATM S712*(0.26%), STOP F133L(26.3%), TP53 V157F(0.20%), H214R(35.9%) |
| 20 | PIK3CA apmlification-equivocla(CN=6), PIK3CB apmlification-equivocla(CN=7), PRKCl apmlification-equivocla(CN=6), SPOT F133L(30%), TERC PIK3CB apmlification-equivocla(CN=6) |
| 21 | PTEN loss, TMPR-ERG fusion, TP53 24S(34.7%), |
| 22 | PTEN E235fs*8(0.26%) |
| 23 | CDK12 P69fs*20(15.4%), MCL1 amplification-equivocal(CN=6) |
| 24 | None |
| 25 | SPOP F125V(22.6%), TP53 Q192_R196del(22.0%) |
| 26 | ATM Y2954C(0.2%), TNFAIP3 L157fs*59(0.34%), DDR1 R522H(0.34%), RAD21 S189fs*2(0.25%),  TP53 R110L(0.63%) |
| 27 | PBRM1 R1052*(18.0%), PTEN loss, TP53 loss, |
| 28 | BACA2 I1874fs*34(59.9) |
| 29 | CIC S1117fs*34(85.4%), MED12 G44D(92.4%) |
| 30 | PIK3R1 rearrangement intron 15, STED2 rearrangement exon 16, TP53 C238Y(35.9%) |
