# Supplementary material for: Fibroblast-mediated KRAS activation in double-negative prostate cancer
Source: Cell Death Dis. 2026 May 2;17(1):403. doi: 10.1038/s41419-026-08800-3 (PMC13139504; doi:10.1038/s41419-026-08800-3)

Fig. 1D. LNCaP / LNCaP-SF / C4-2B / PC-3 / DU145

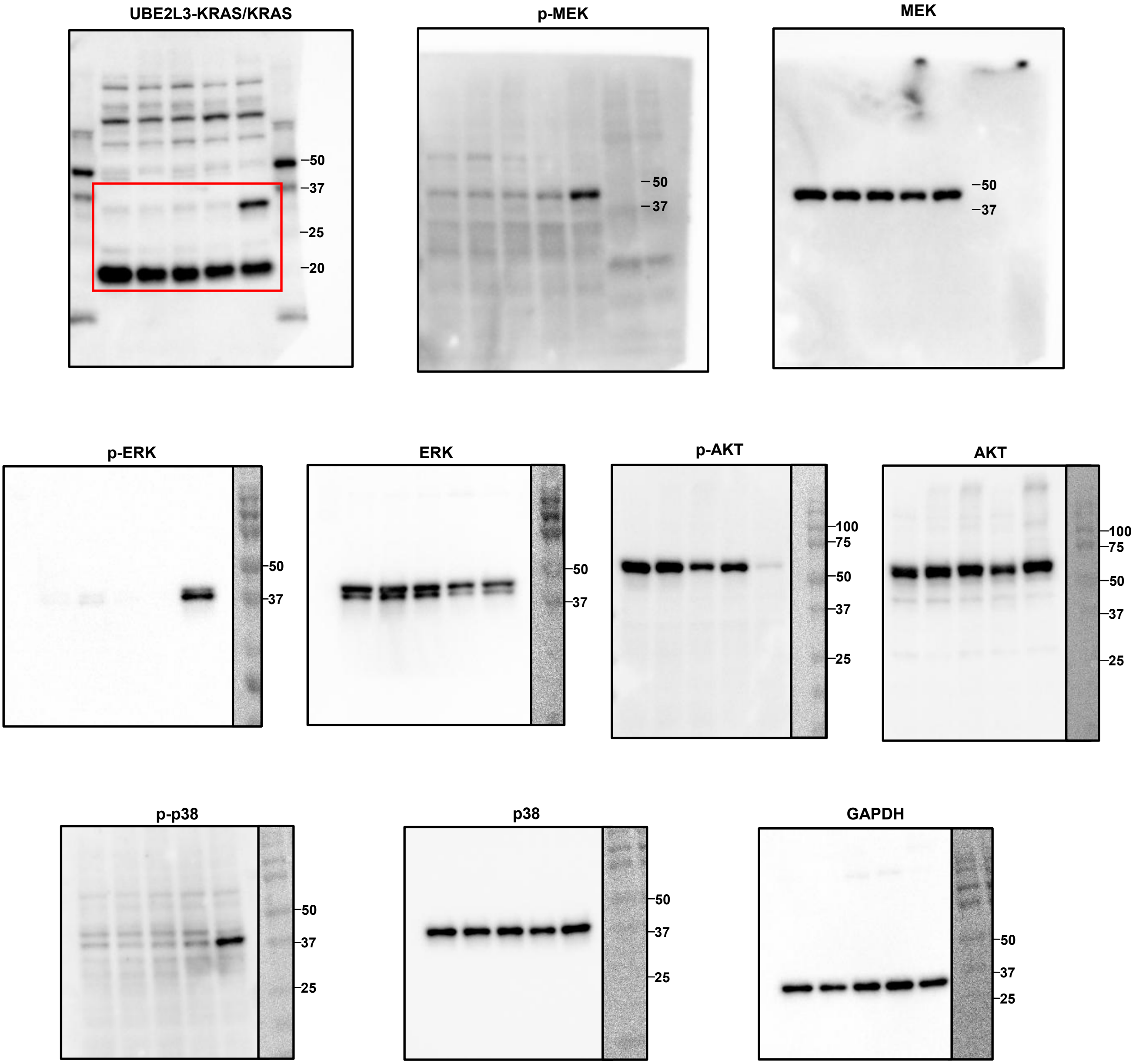

Fig. 1F. LNCaP: NC / si-KRAS #1 / si-KRAS #2 / si-KRAS #3

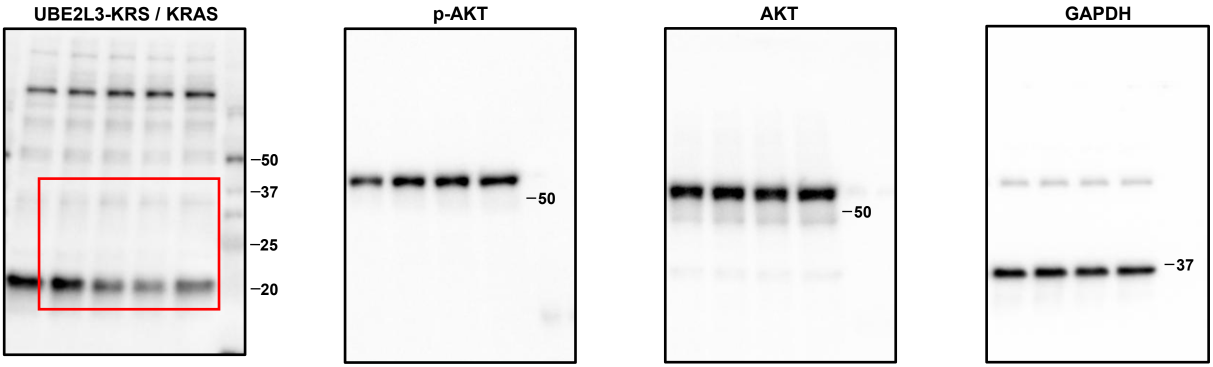

Fig. 1F. DU145: NC / si-KRAS #1 / si-KRAS #2 / si-KRAS #3

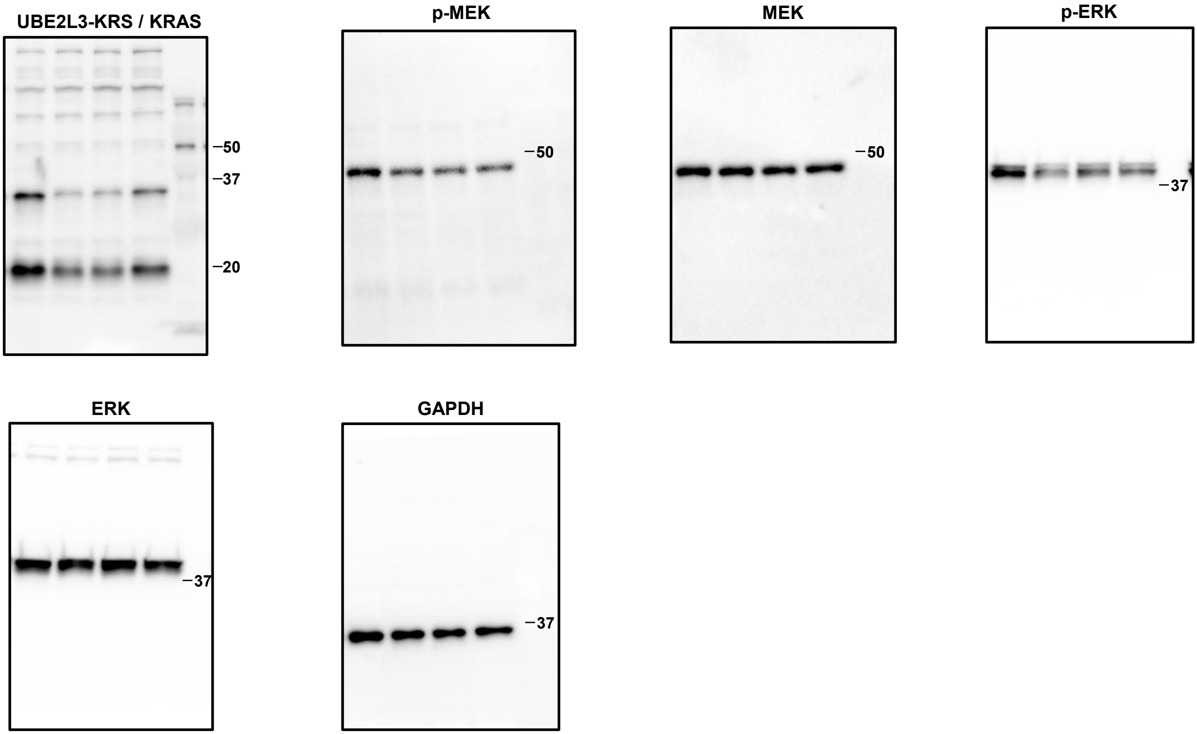

Fig. 2A. LNCaP: FGF8b 0 / 1 / 2 / 5 / 10 / 20 / 40 mg/mL

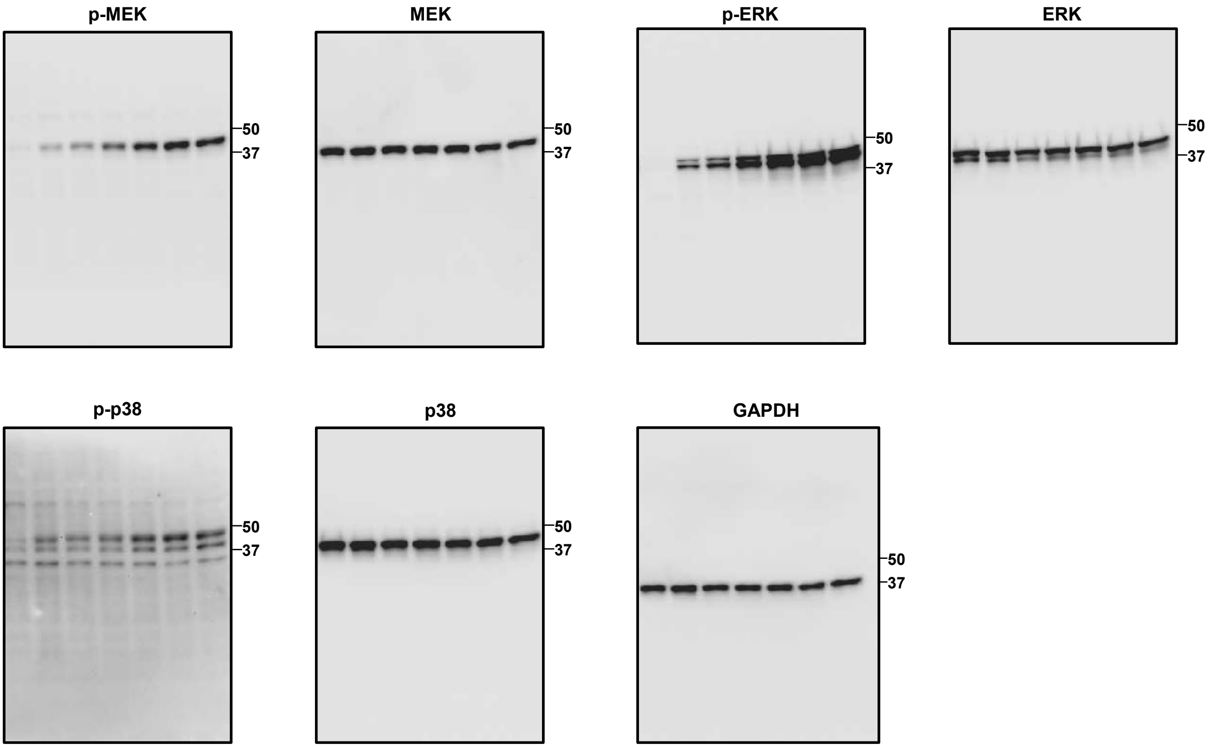

Fig. 2B. LNCaP: EGF 0 / 1 / 2 / 5 / 10 / 20 / 40 mg/mL

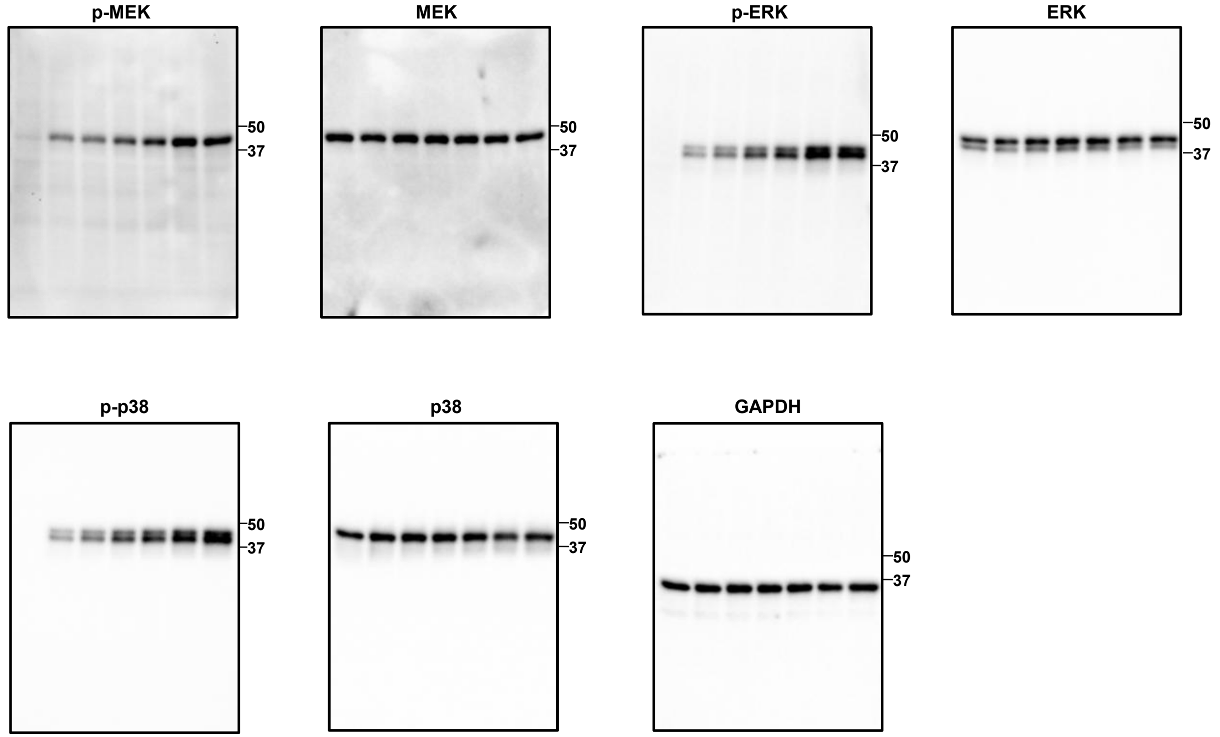

Fig. 2C. FGF8b: LNCaP(-/+) / LNCaP-SF(-/+) / C4-2B(-/+) / PC-3(-/+) / DU145(-/+)

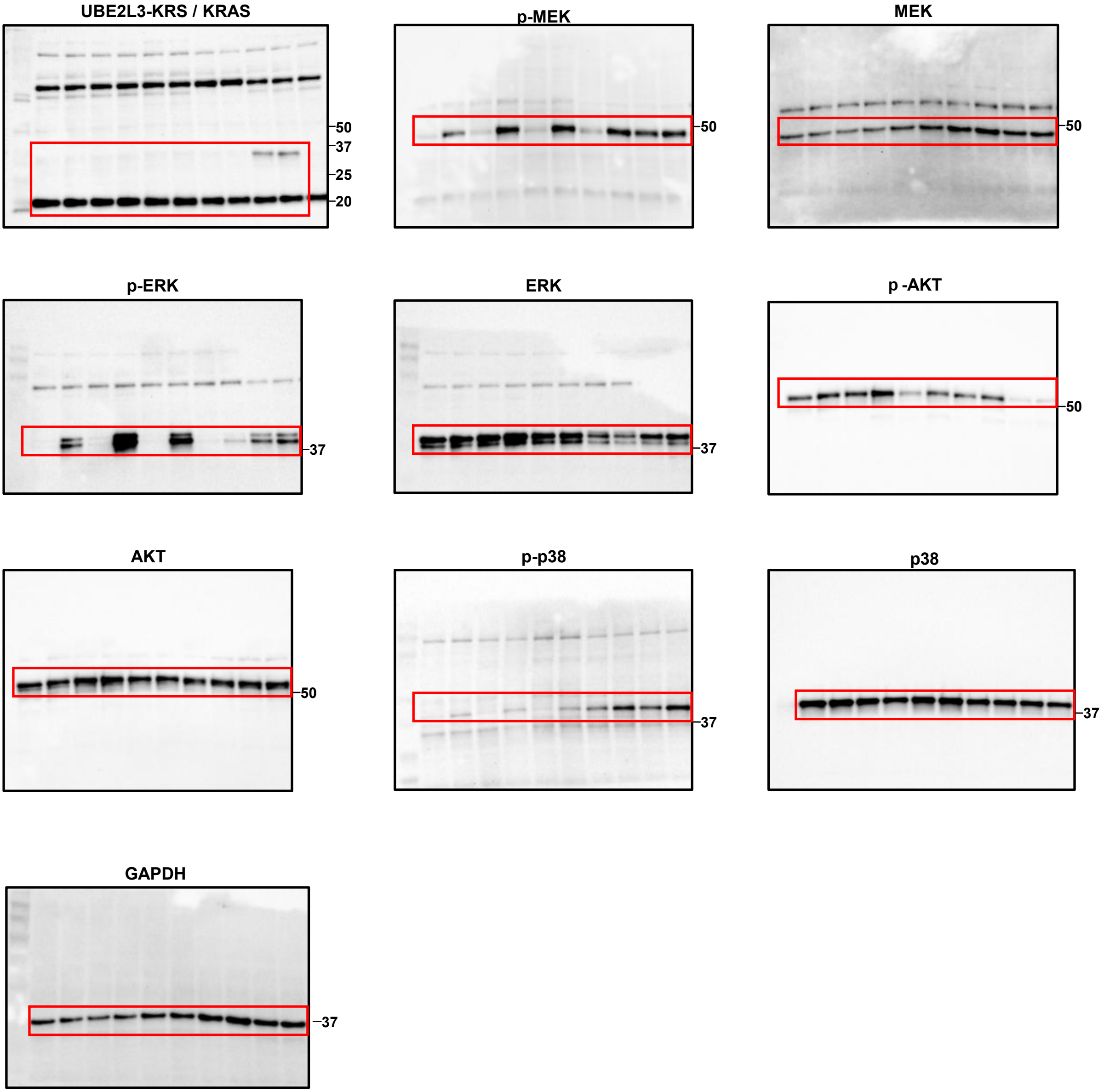

Fig. 2D. EGF: LNCaP(-/+) / LNCaP-SF(-/+) / C4-2B(-/+) / PC-3(-/+) / DU145(-/+)

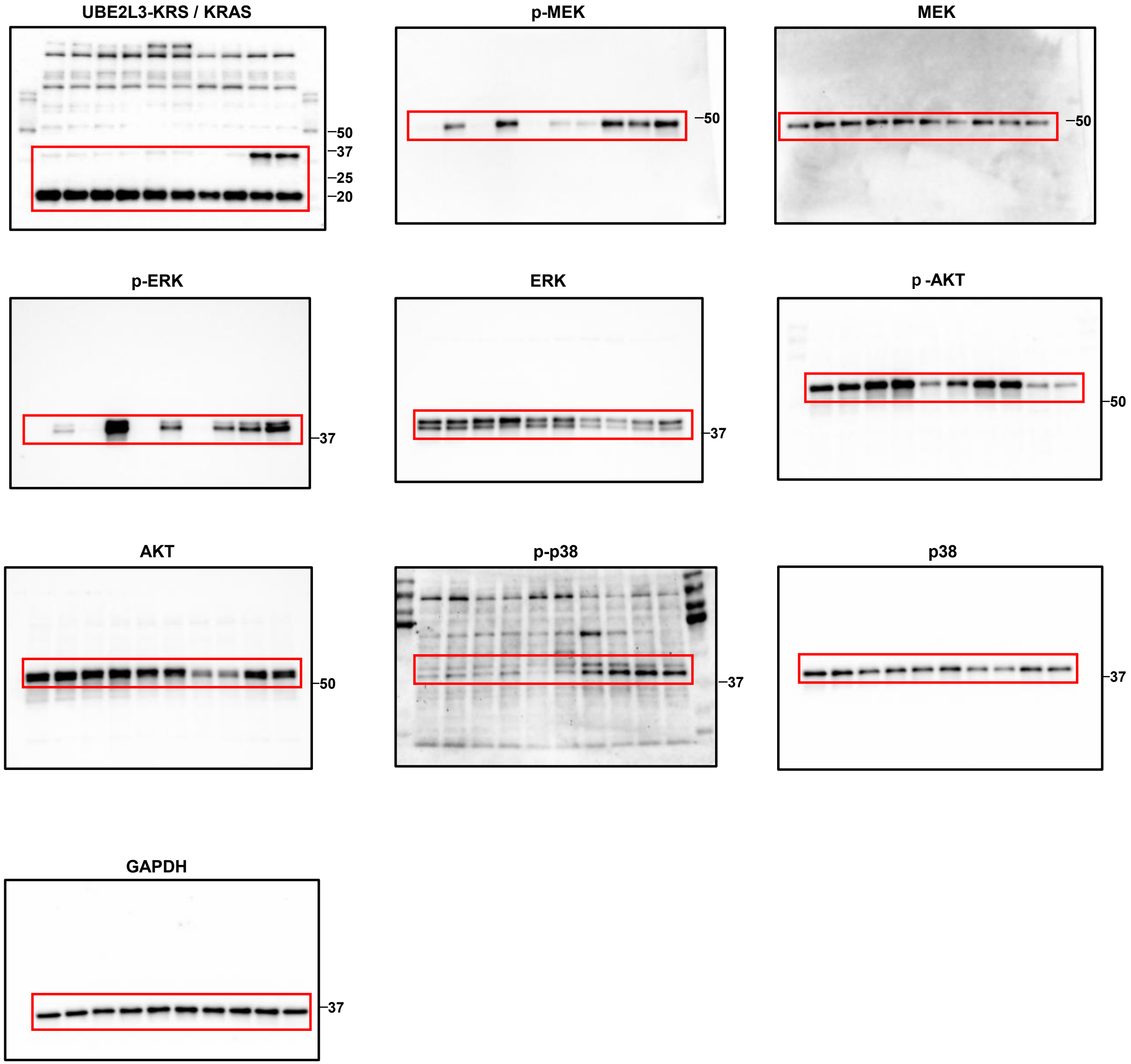

Fig. 2I. LNCaP: - / FGF8b / FGF8b+NC / FGF8b+si-KRAS #1 / FGF8b+si-KRAS #2

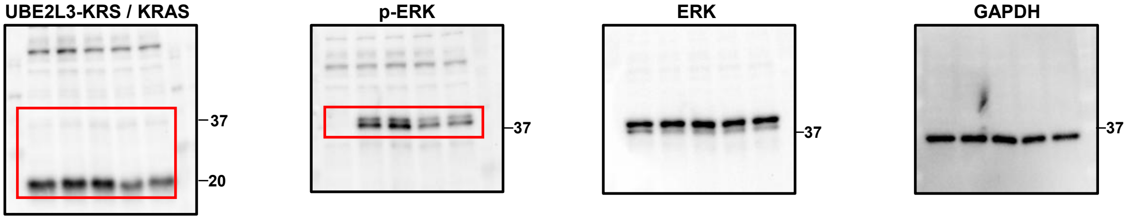

Fig. 2I. DU145: - / FGF8b / FGF8b+NC / FGF8b+si-KRAS #1 / FGF8b+si-KRAS #2

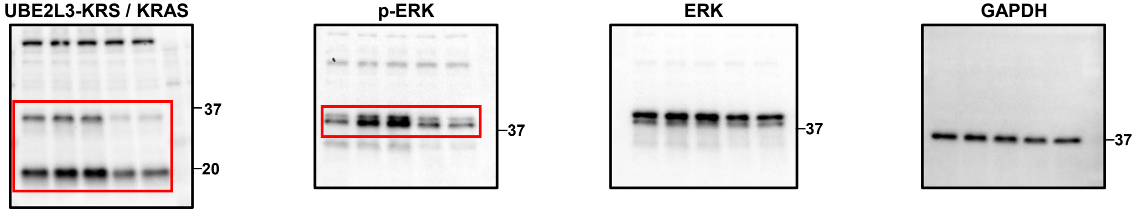

Fig. 2J. LNCaP: - / EGF / EGF+NC / EGF+si-KRAS #1 / EGF+si-KRAS #2

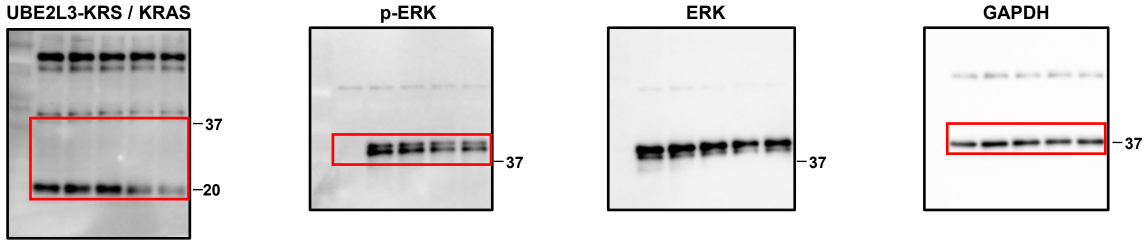

Fig. 2J. DU145: - / EGF / EGF+NC / EGF+si-KRAS #1 / EGF+si-KRAS #2

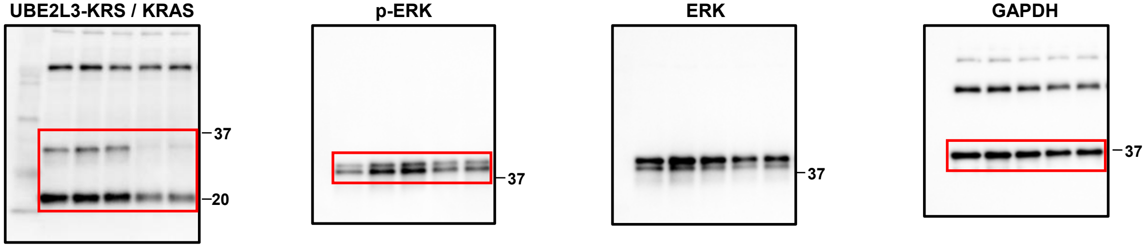

Fig. 3B. DU145, B-I3406: - / DMSO / 5min / 10min / 0.5h / 1h / 2h / 3h / 6h / 12h / 24h

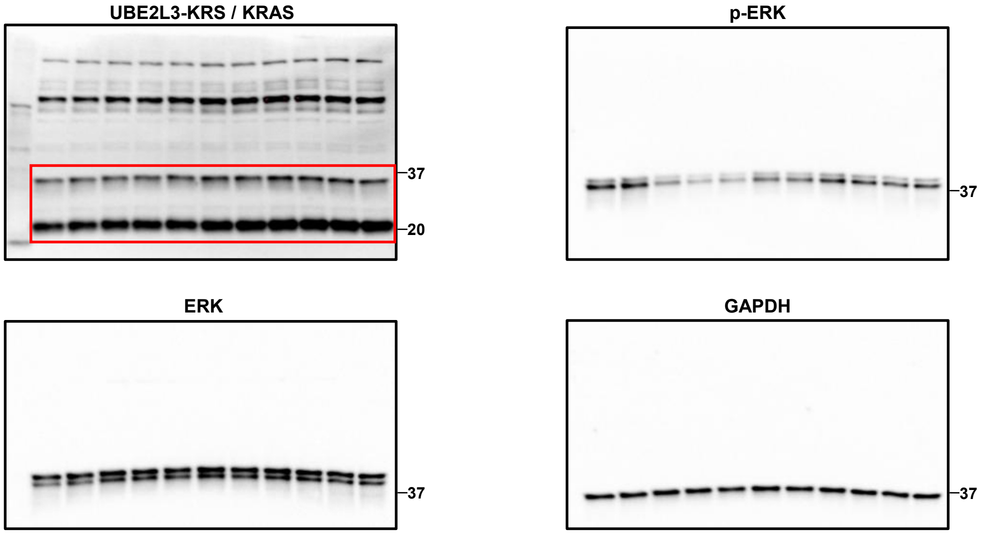

Fig. 3C. DU145, B-I3406(μM): DMSO / 0.01 / 0.1 / 1 / 10

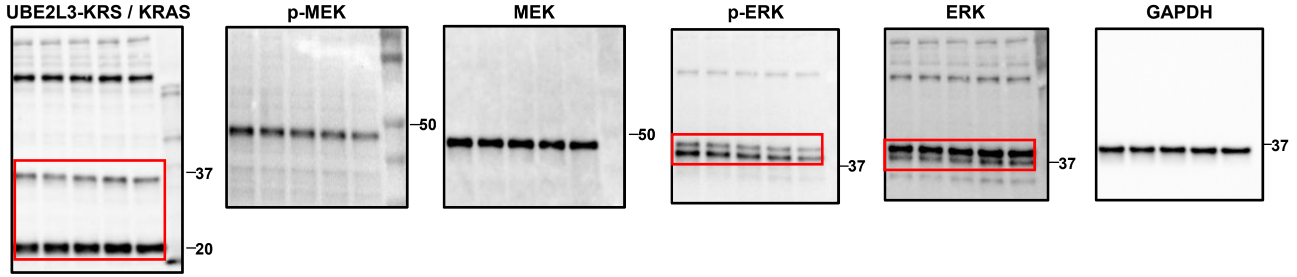

Fig. 3D. DU145: - / FGF8b / BI-3406 0.1μM / FGF8b+BI-3406 0.1μM / BI-3406 1μM / FGF8b+BI-3406 1μM

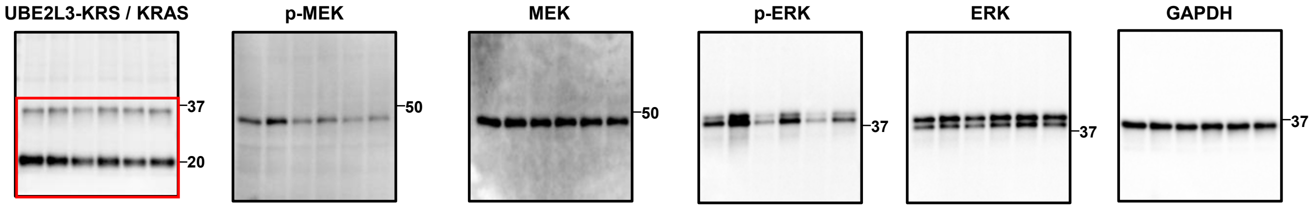

Fig. 3E. DU145: - / EGF / BI-3406 0.1μM / EGF+BI-3406 0.1μM / BI-3406 1μM / EGF+BI-3406 1μM

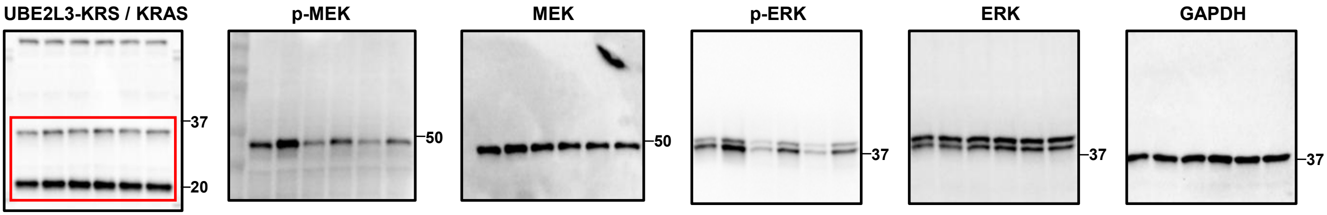

Fig. 3F. LNCaP: - / FGF8b / BI-3406 / FGF8b+BI-3406, LNCaP-SF : - / FGF8b / BI-3406 / FGF8b+BI-3406

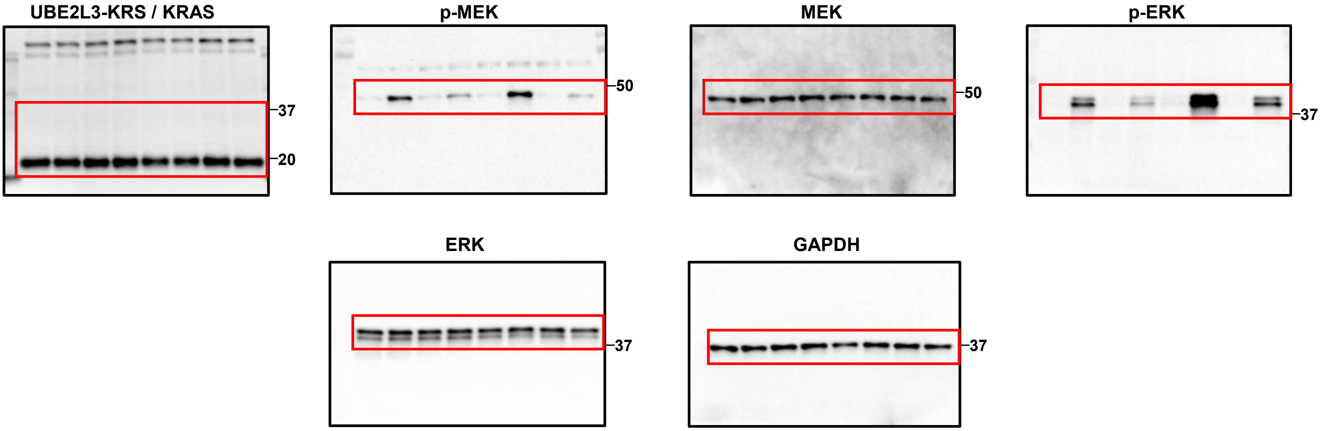

Fig. 3G. LNCaP: - / EGF / BI-3406 / EGF+BI-3406, LNCaP-SF : - / EGF / BI-3406 / EGF+BI-3406

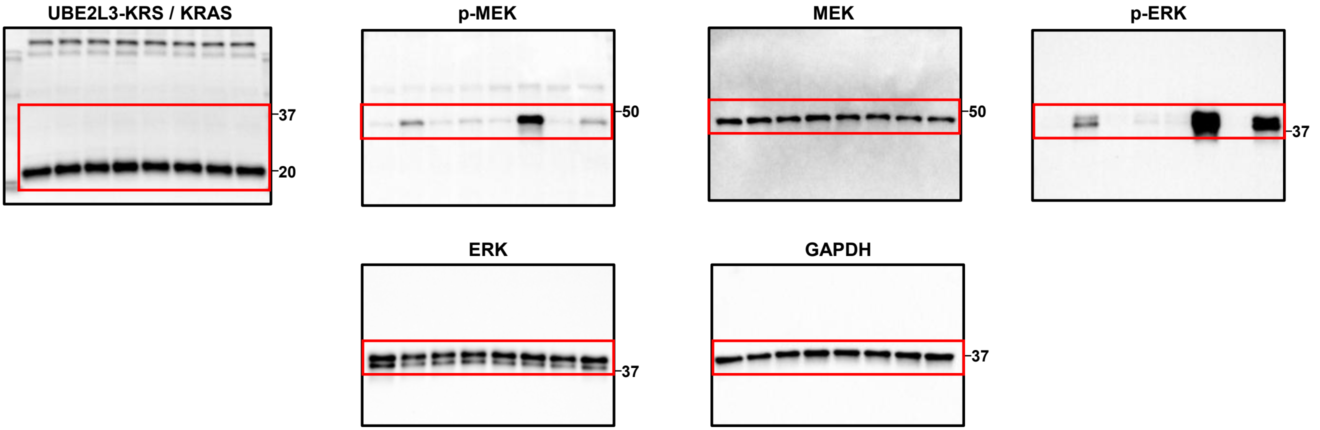

Fig. 4A. LNCaP / LNCaP-SF / C4-2B / PC-3 / DU145

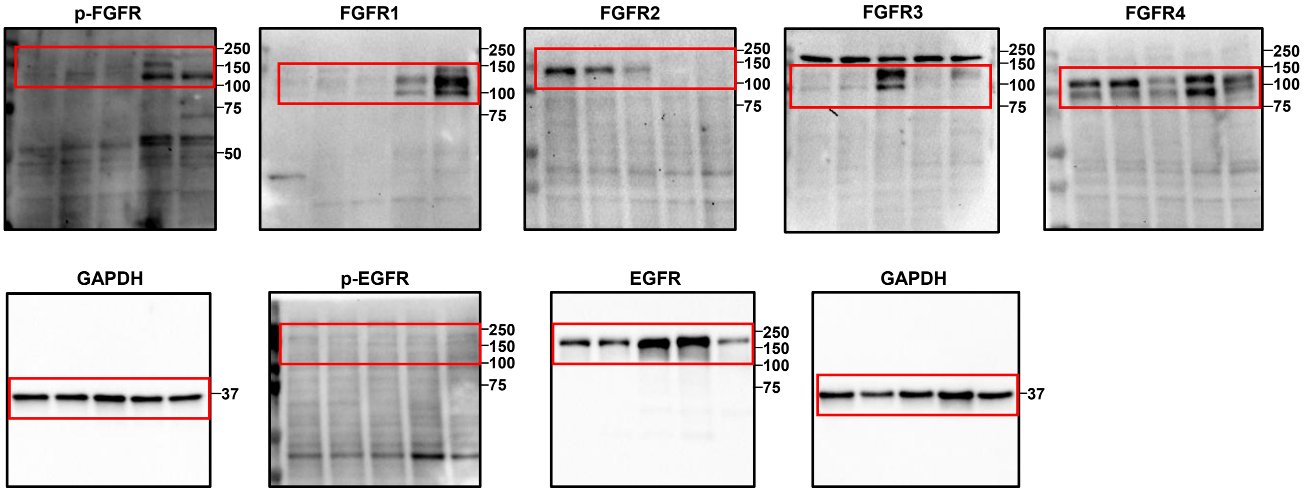

Fig. 4B. LNCaP: NC / si-AR #1 / si-AR #2 / si-AR #3

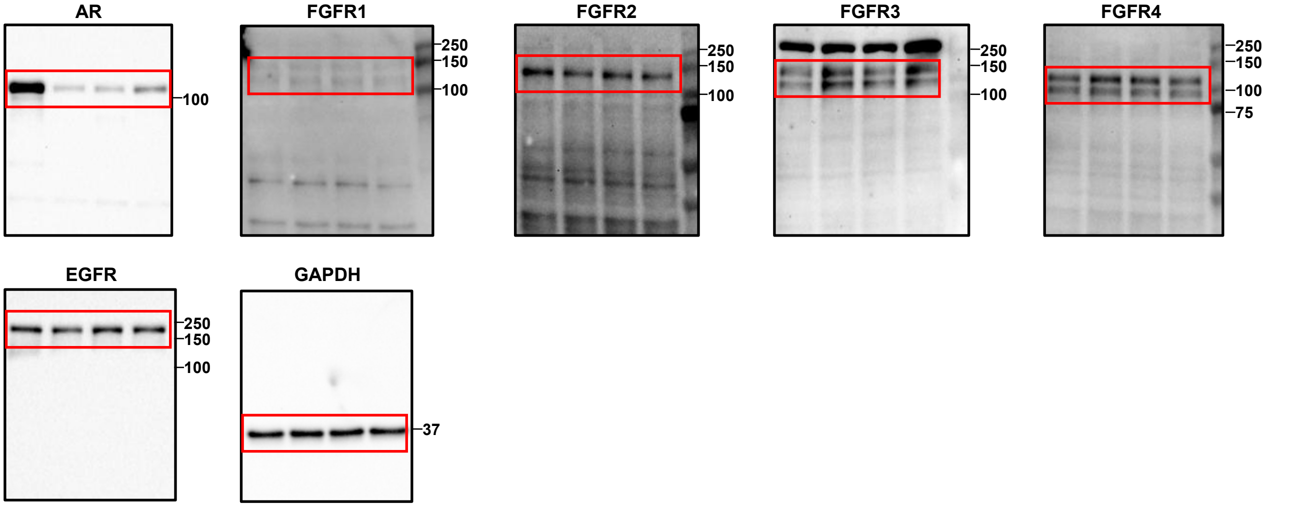

Fig. 4C. LNCaP: NC / NC+FGF8b / si-AR #1 / si-AR #1+FGF8b / si-AR #2 / si-AR #2+FGF8b

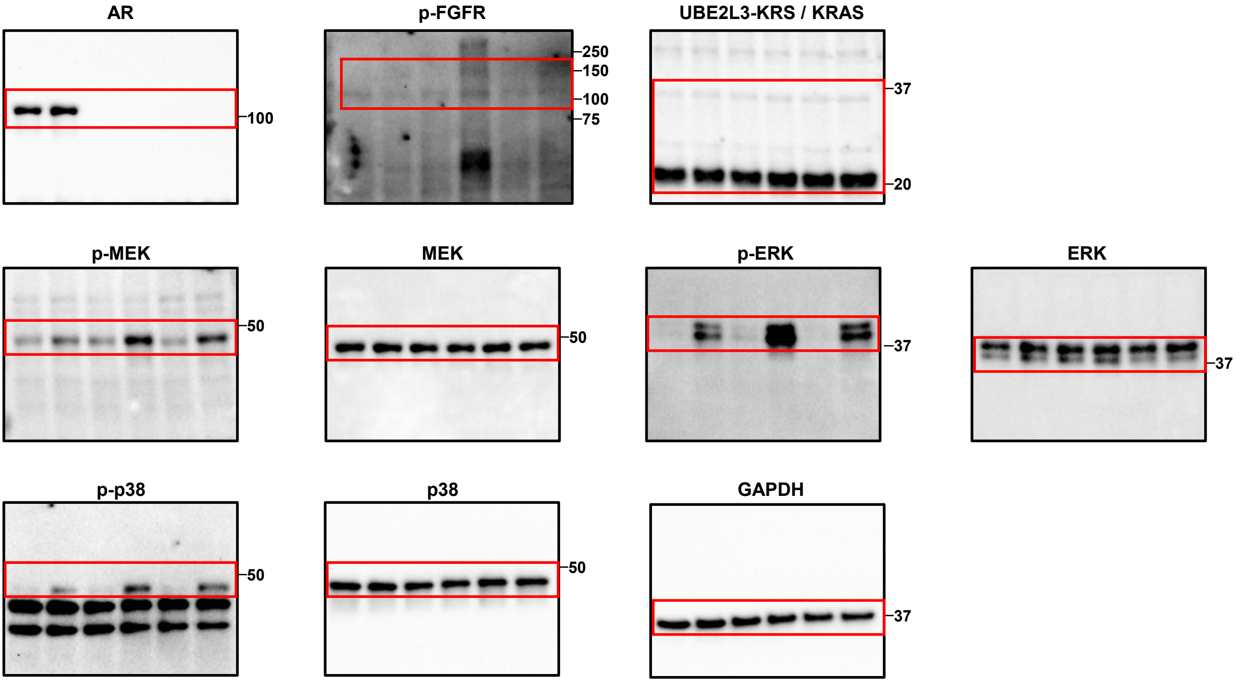

Fig. 5F. PrSC / PCaSCv2-5 / PCaSCv2-6 / PCaSCv2-4 / PCaSCv2-9

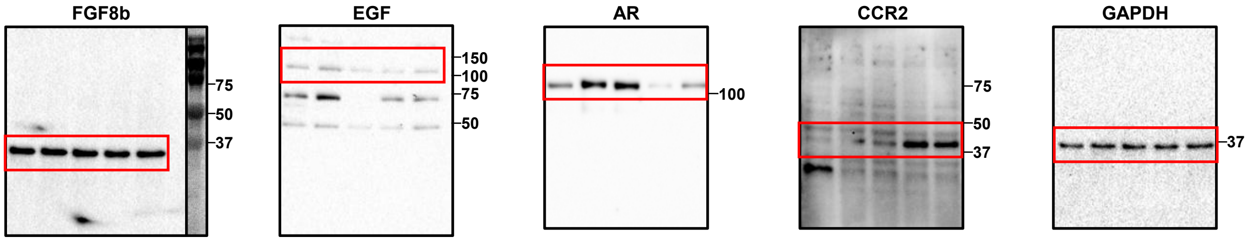

Fig. 5G. LNCaP / LNCaP-SF / DU145

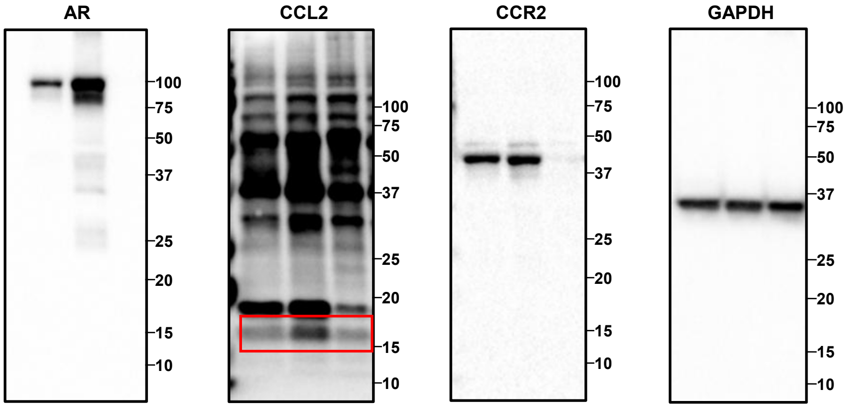

Fig. 5H. LNCaP: NC / si-AR#1 / si-AR#2

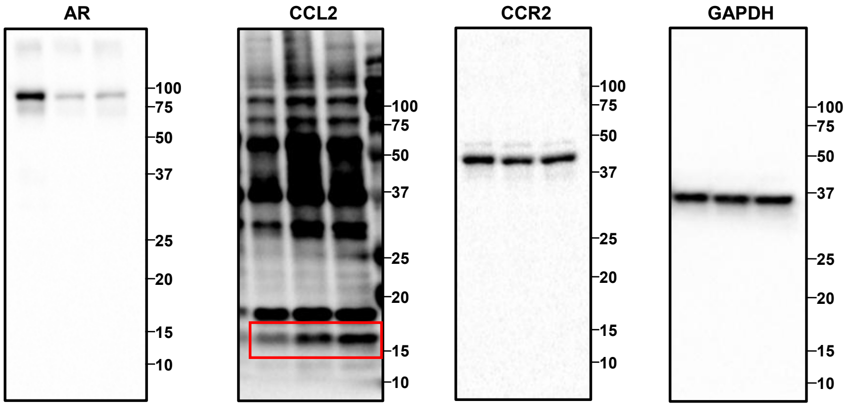

Fig. 5L. PrSC: CCL2(h), - / 0.5 / 1 / 3 / 6 / 24

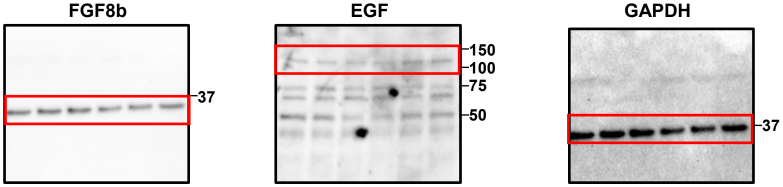

Fig. 5L. PCaSCv2-5: CCL2(h), - / 0.5 / 1 / 3 / 6 / 24

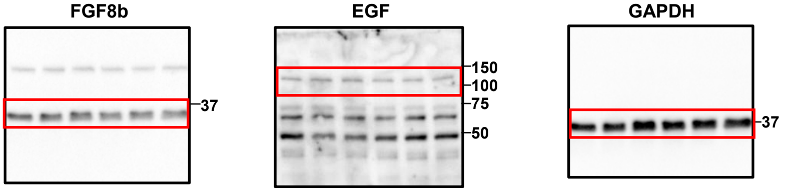

Fig. 5L. PCaSCv2-9: CCL2(h), - / 0.5 / 1 / 3 / 6 / 24

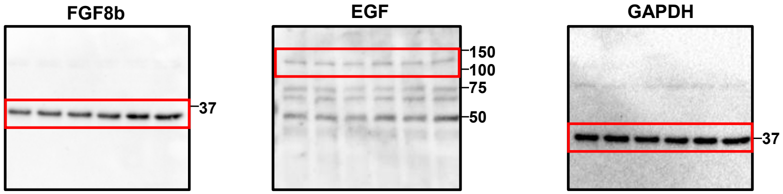

Fig. 5O. PCaSCv2-9: CCL2, - / +

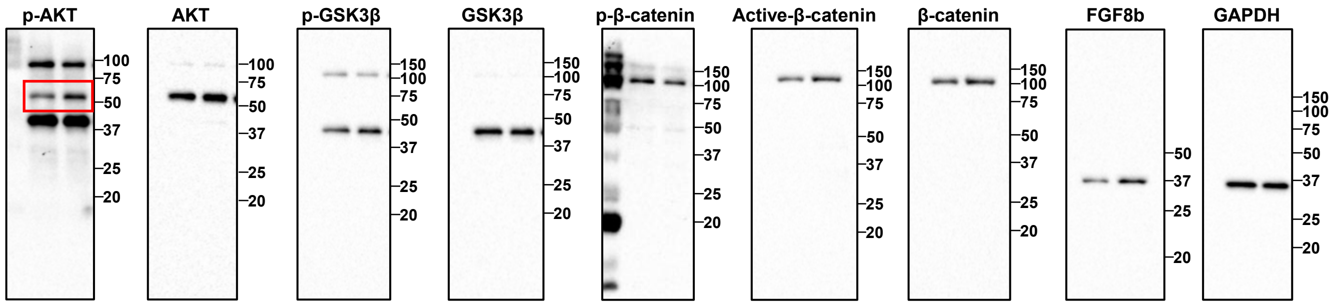

Fig. 5P. PCaSCv2-9: LNCaP(-/+) / LNCaP-SF(-/+) / C4-2B(-/+) / PC-3(-/+) / DU145(-/+)

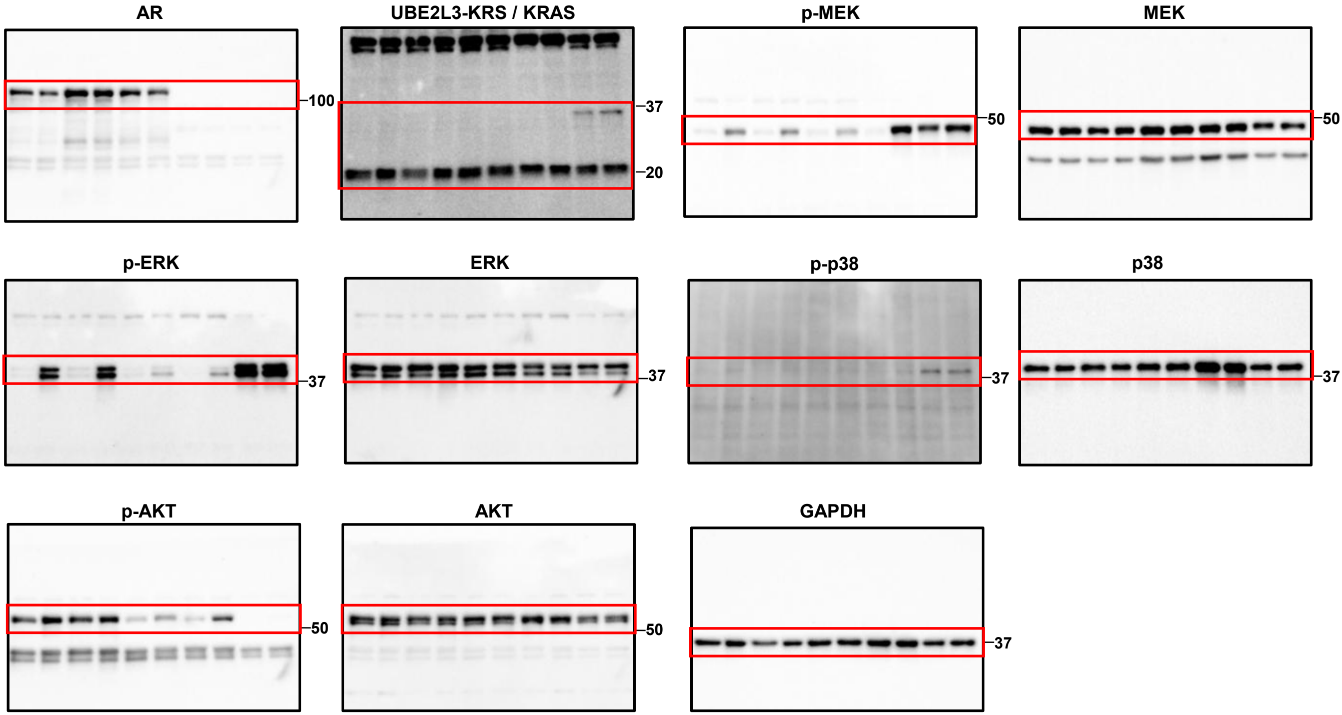

Fig. 5P. PCaSCv2-9: LNCaP(-/+) / LNCaP-SF(-/+) / C4-2B(-/+) / PC-3(-/+) / DU145(-/+)

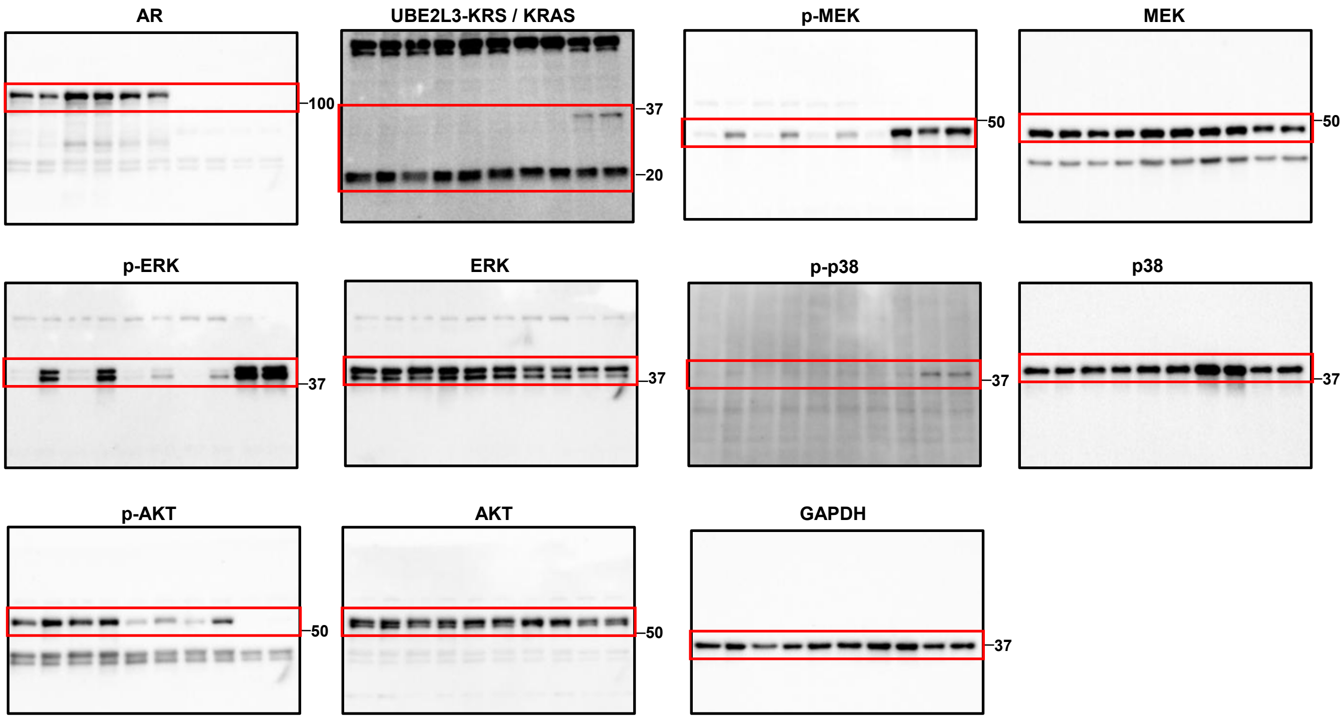

Fig. 5Q. LNCaP-SF

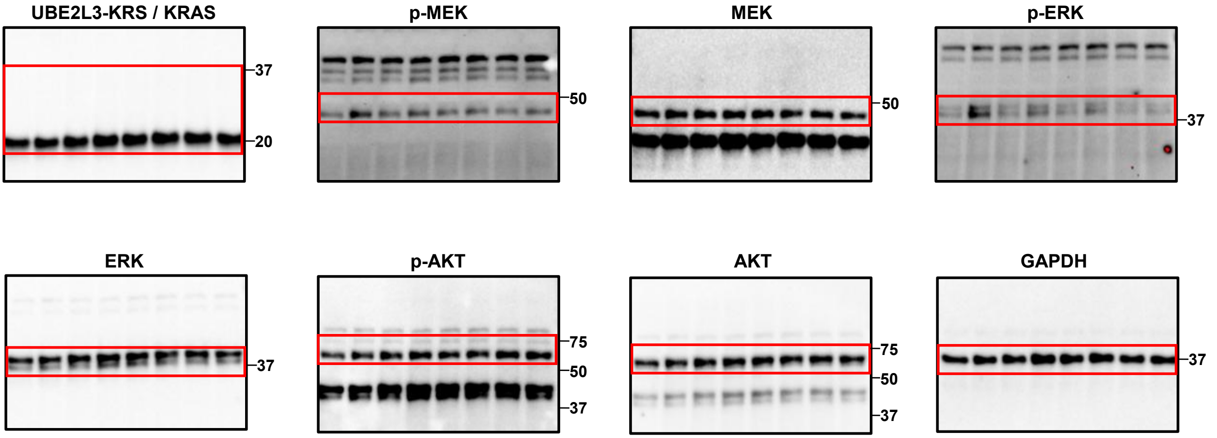

Fig. 5Q. DU145

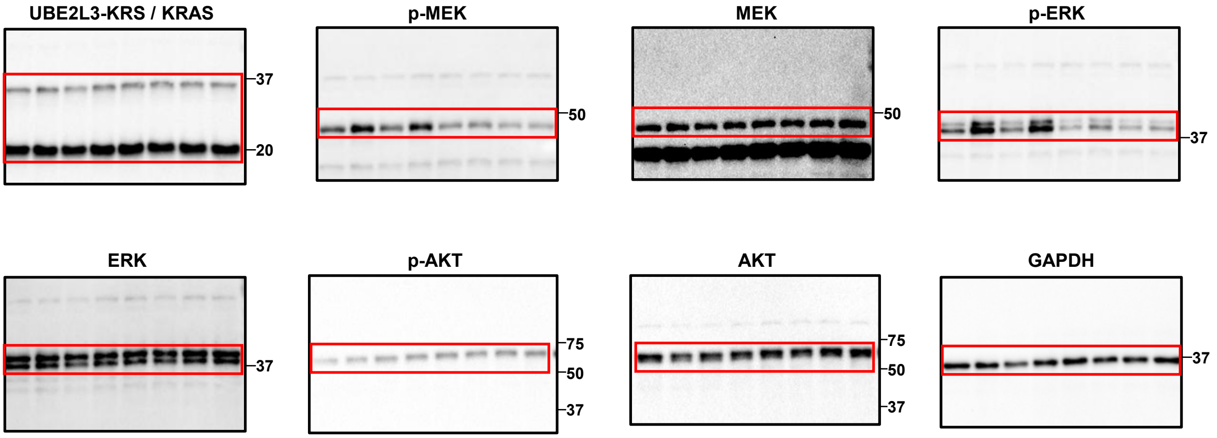

Fig. S2A. LNCaP

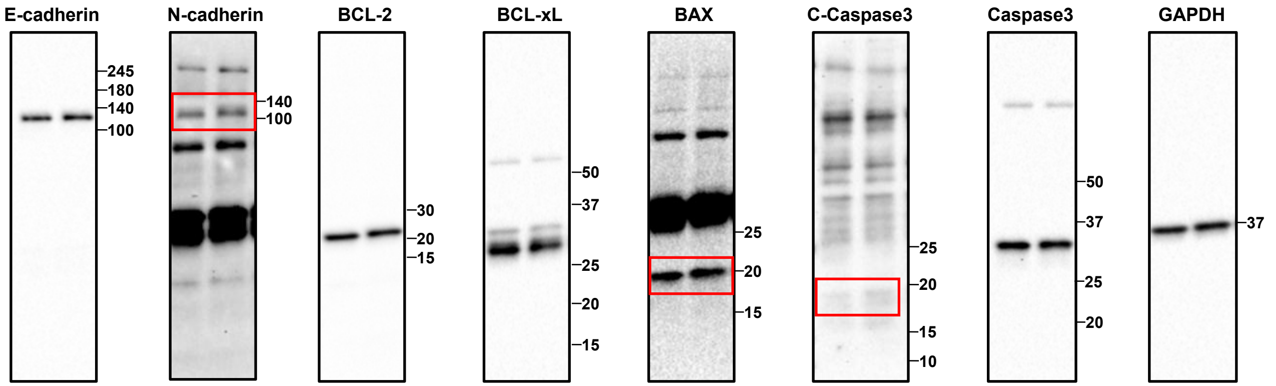

Fig. S2A. DU145

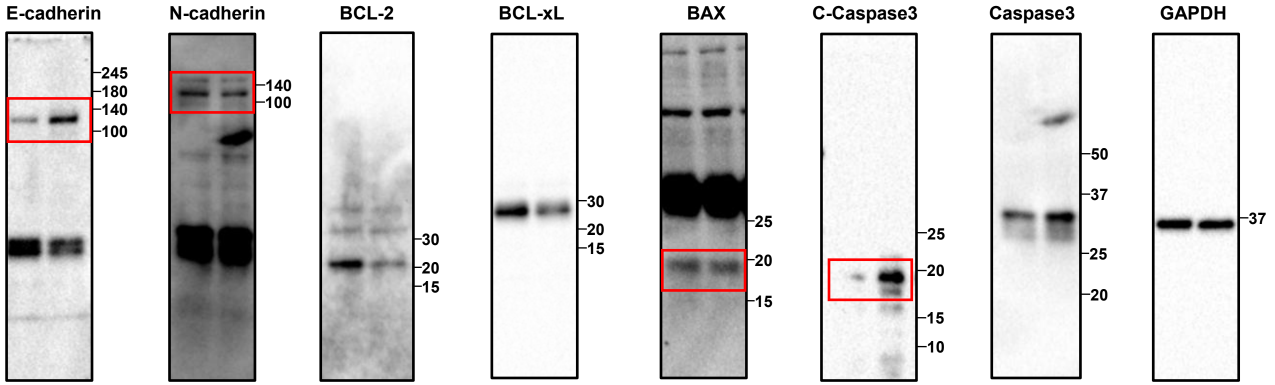

Fig. S6. LNCaP : CCL2 0 / 0.3 / 1 / 3 / 10 / 30 mg/mL

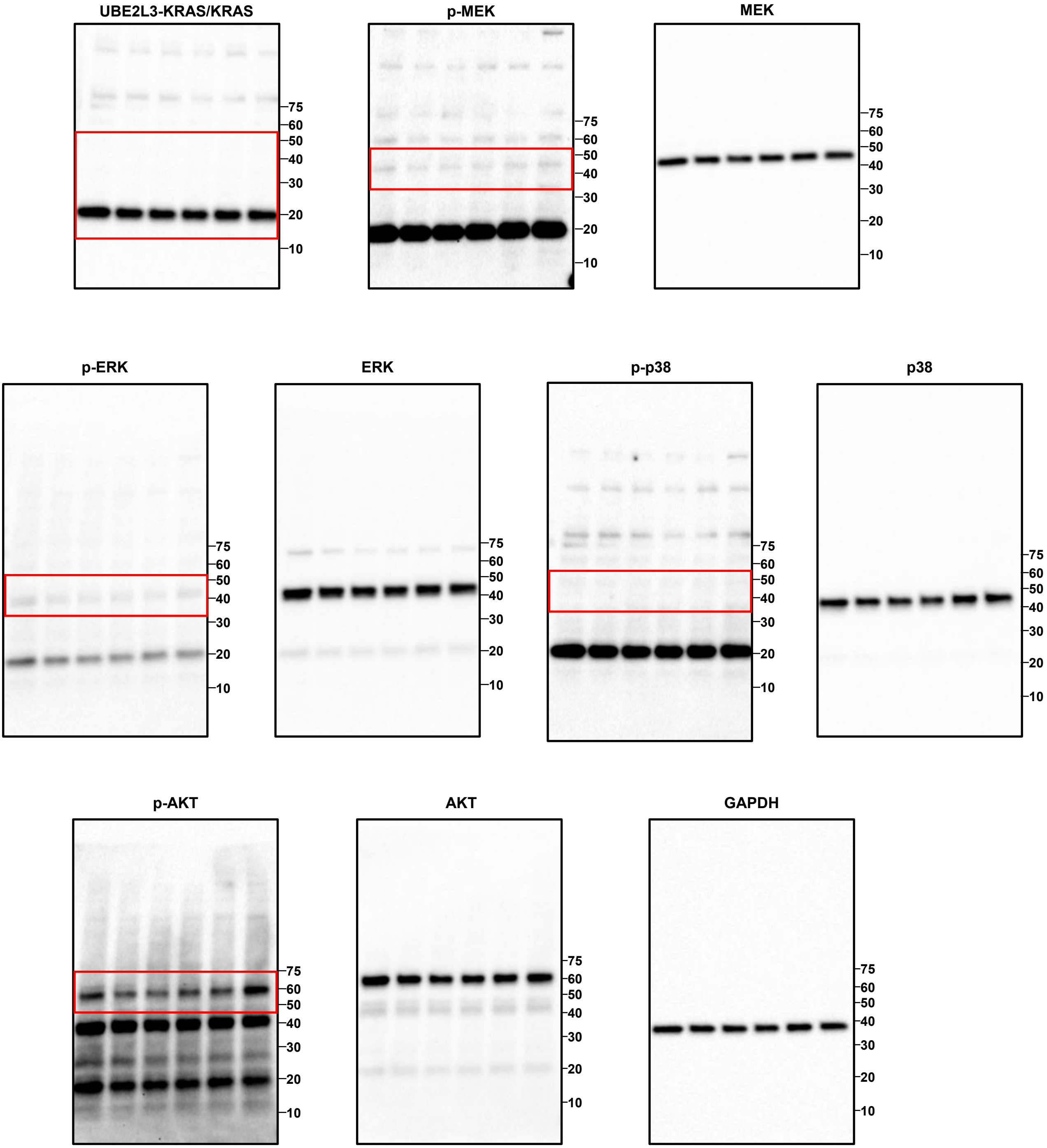

Fig. S6. LNCaP-SF : CCL2 0 / 0.3 / 1 / 3 / 10 / 30 mg/mL

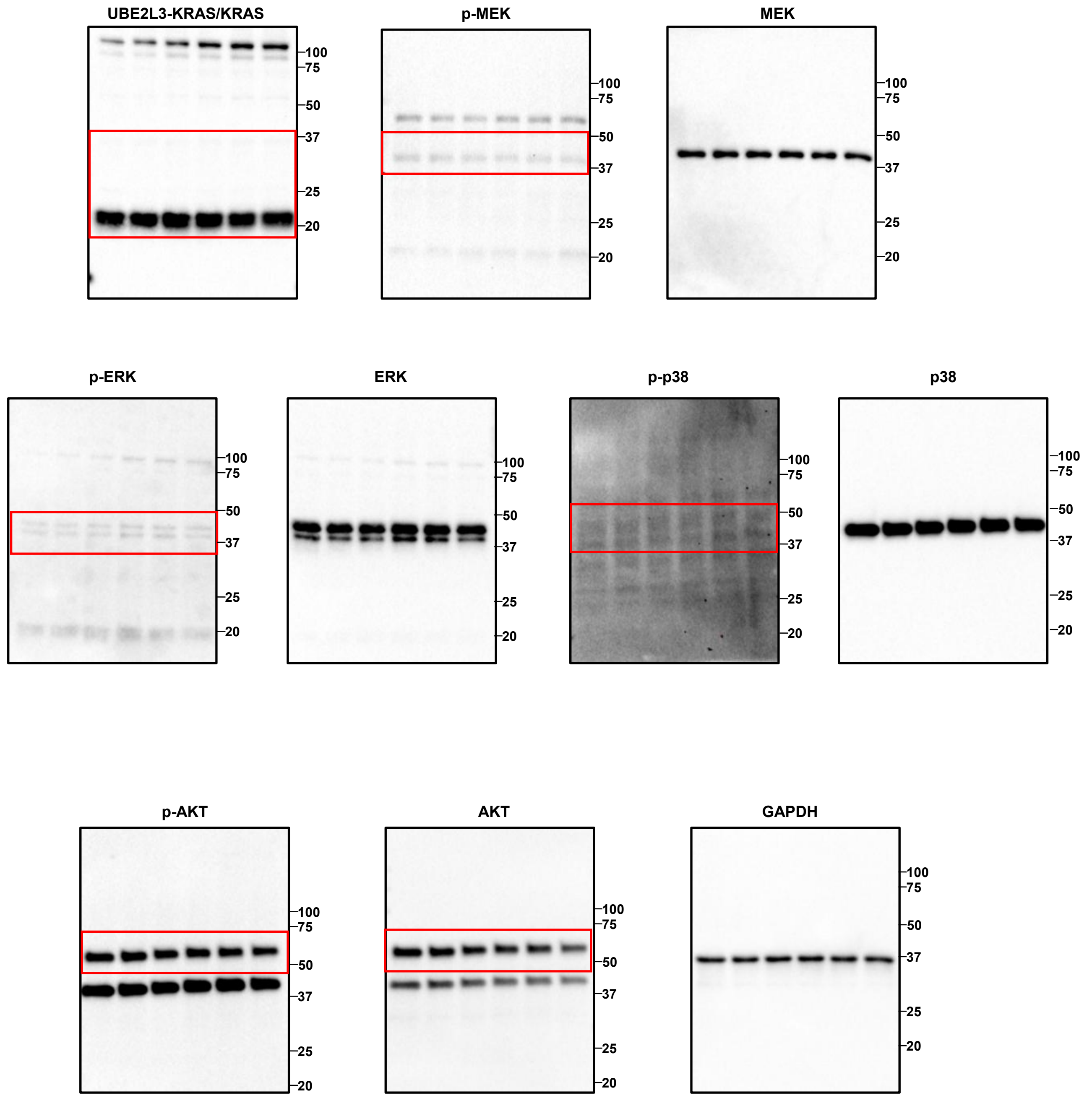

Fig. S6. DU145 : CCL2 0 / 0.3 / 1 / 3 / 10 / 30 mg/mL

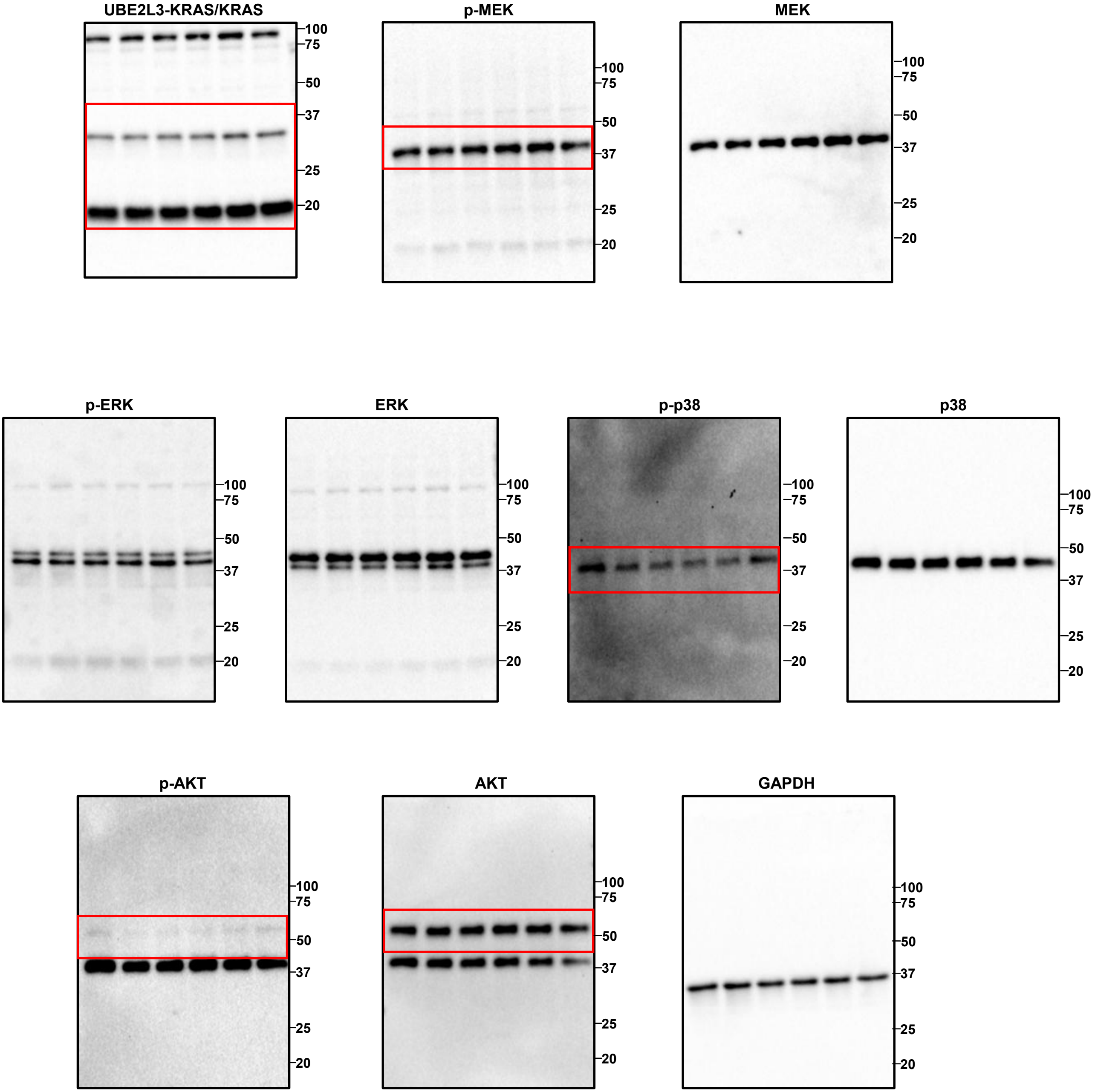

Fig. S7A. PrSC / PCaSCv2-5 / PCaSCv2-6 / PCaSCv2-4 / PCaSCv2-9

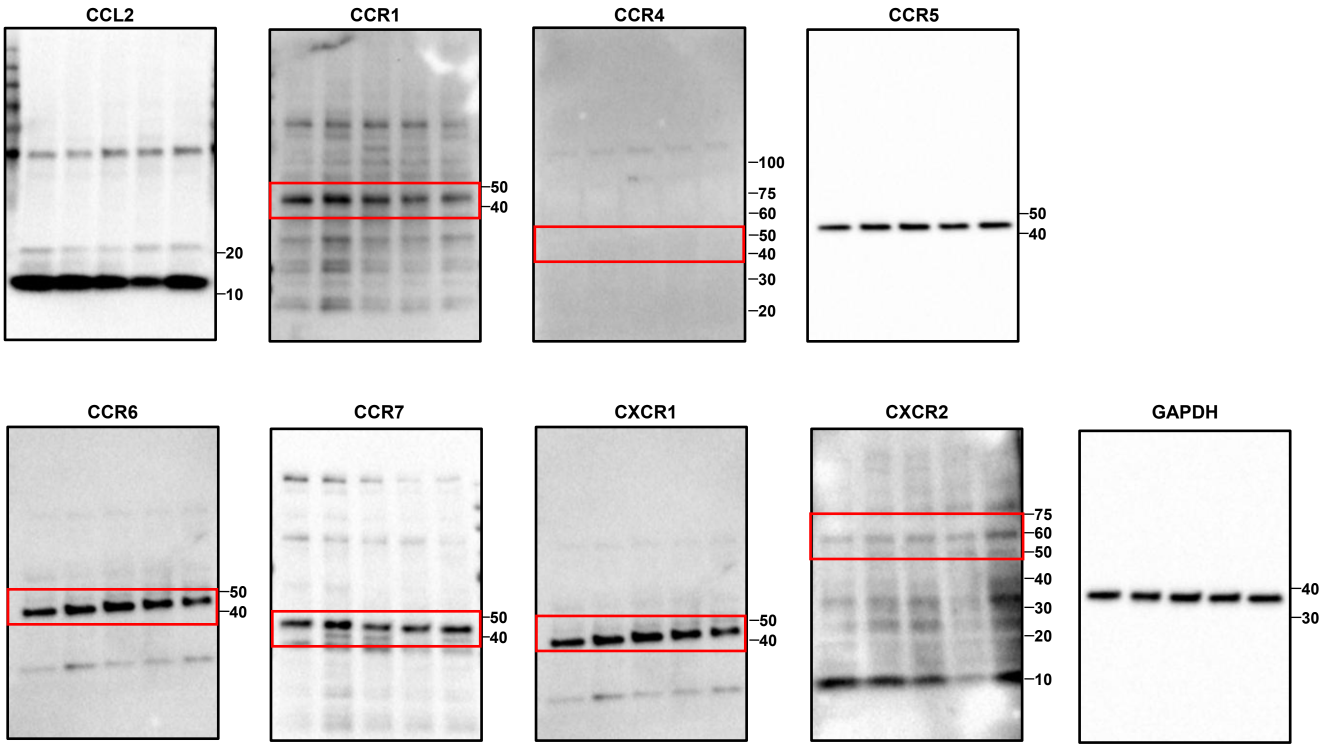

Fig. S7B. PCaSCv2-9: - / LNCaP / LNCaP-SF / DU145

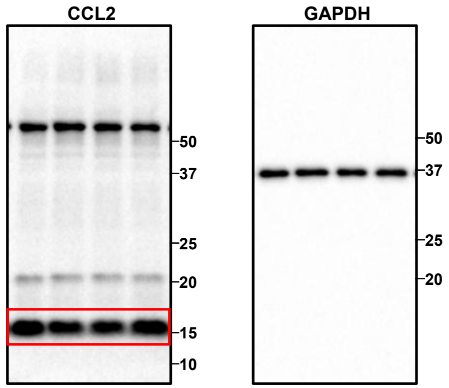

Fig. S7F. PCaSCv2-9

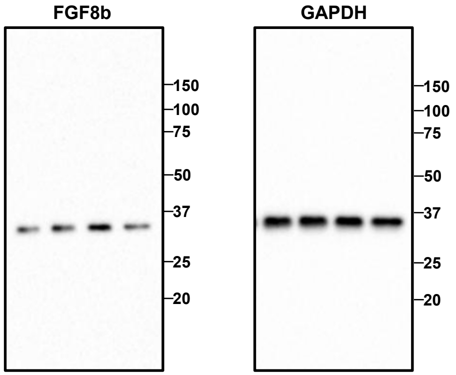

Fig. S7I. PCaSCv2-9

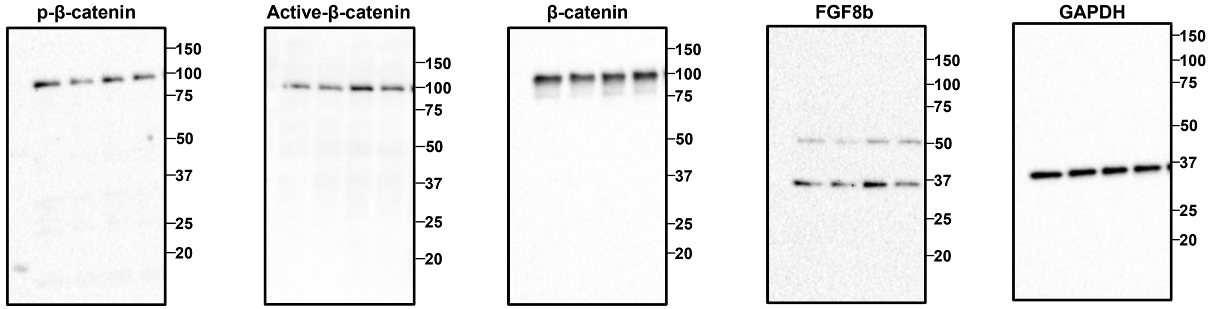

Fig. S8A. LNCaP-SF

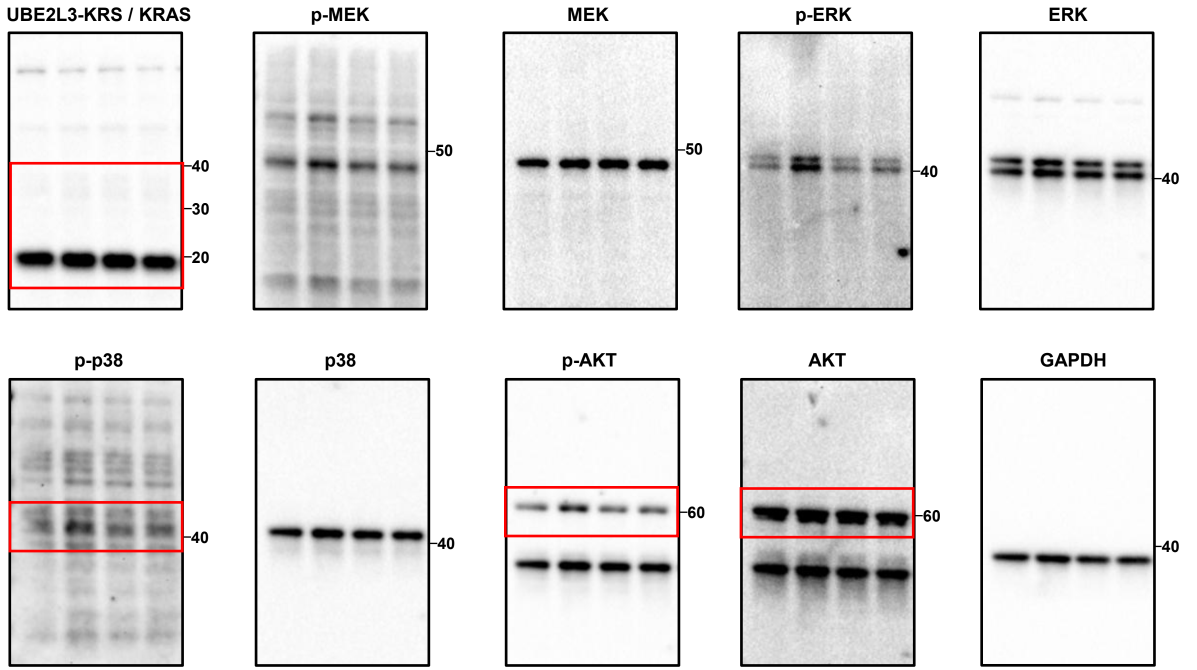

Fig. S8A. DU145

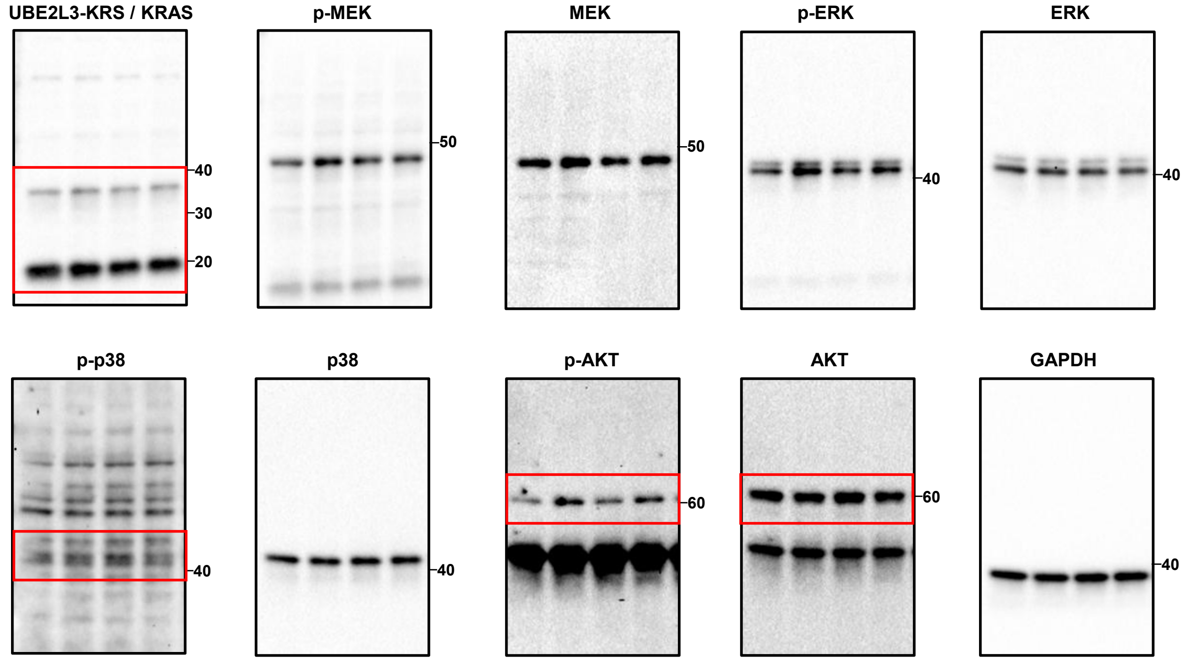

Fig. S8B. LNCaP-SF

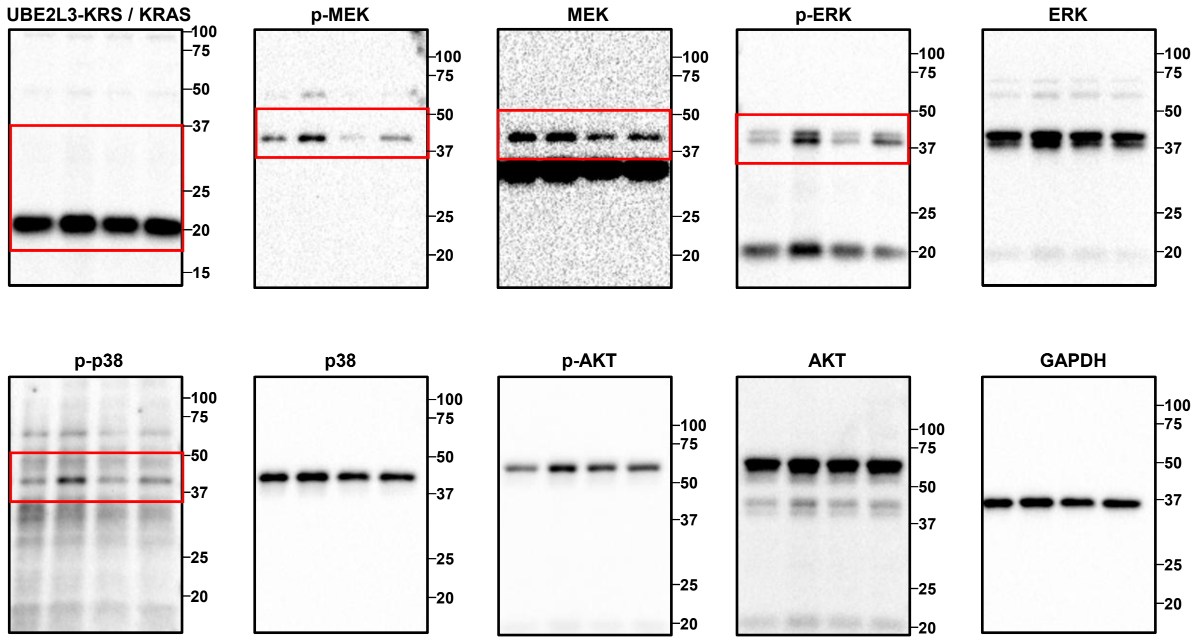

Fig. S8B. DU145

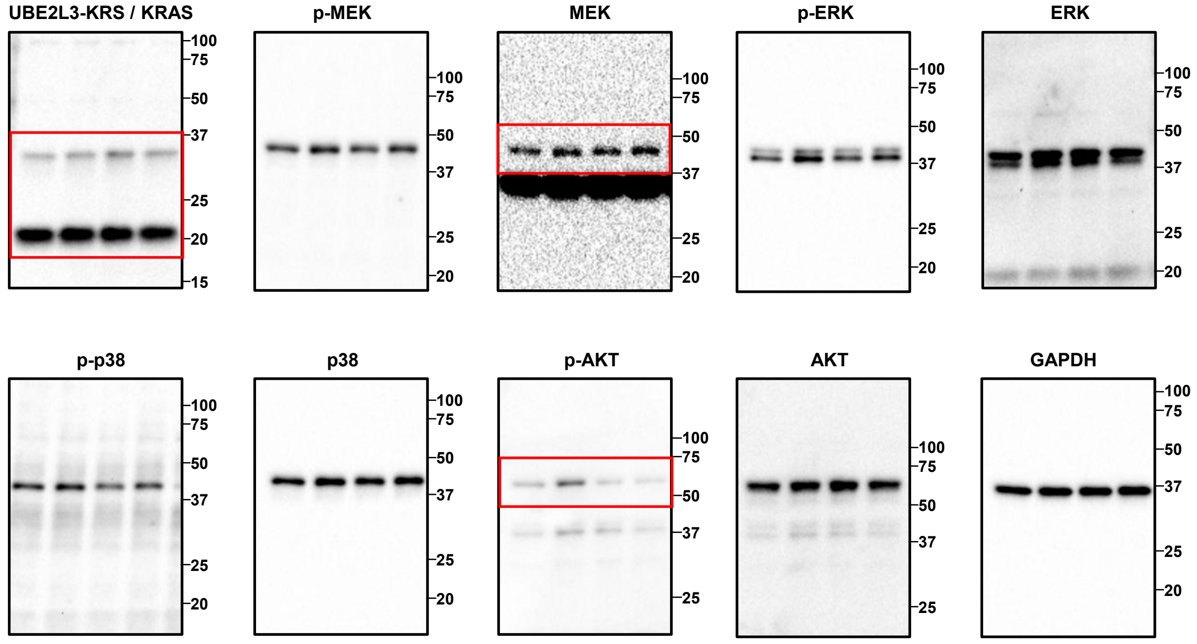

Supplement: Supplementary file 2 — Full blot of WB [file 41419_2026_8800_MOESM2_ESM.pdf]
